# Enhanced digestion and absorption kinetics of ultra–low molecular weight collagen containing di- and tripeptides: evidence from human and cell models

**DOI:** 10.7717/peerj.21398

**Published:** 2026-08-14

**Authors:** Reyhan Nergiz-Unal, Stephan Dierckx, Chiara Roye, Yingying Wu, Faye Maertens, David Pajuelo Gamez, Marian Merino, Jose Luis Mullor, Bengu Depboylu, Carlos Daniel Mandolesi

**Affiliations:** 1 Tessenderlo Innovation Center, Tessenderlo Group NV, Brussels, Belgium; 2 PB Leiner, Part of Tessenderlo Group, Brussels, Belgium; 3 Faculty of Health Sciences, Universidad Europea de Valencia, Valencia, Spain; 4 Bionos Biotech SL., LabAnalysis Life Science, Valencia, Spain; 5 Faculty of Medicine, Adnan Menderes University, Aydın, Turkiye

**Keywords:** Collagen hydrolysate, Bioavailability of di- and tripeptides, Caco-2 cell model, Human plasma, Ultra-low molecular weight collagen peptides, Food supplement

## Abstract

**Background:**

Collagen hydrolysates differ in molecular weight distribution and composition, which may influence gastrointestinal absorption and epithelial transport.

**Objective:**

The objective of this study is to compare the digestion and absorption kinetics of an ultra-low molecular weight collagen with >45% di- and tripeptides (LMWCP; 500 Da) and a standard collagen hydrolysate (CP; 2,000–3,000 Da) using a Caco-2 cell model and an exploratory human study.

**Materials and methods:**

A Caco-2 cell Transwell model was used to assess the apical-to-basolateral appearance of collagen-derived free amino acids over 240 minutes after *in vitro* digestion of the test products. Product characterization included quantification of free amino acids and selected hydroxyproline-containing di- and tripeptides. In parallel, an exploratory single-blind, parallel-group human study in 15 healthy male participants measured postprandial plasma free amino acid kinetics at 0, 15, 30, 120, and 240 minutes after ingestion of LMWCP, CP, or placebo.

**Results:**

In both models, LMWCP showed faster early-phase kinetics than CP. In the Caco-2 model, collagen-derived amino acids appeared in the basolateral compartment within the first minutes and were highest during the first 30 minutes with LMWCP. In the human study, plasma glycine and proline/hydroxyproline peaked earlier after LMWCP group (30 minutes) compared to CP group (120 minutes).

**Conclusion:**

Across both *in vitro* and *in vivo* models, ultra-low-molecular-weight collagen (LMWCP; 500 Da), with more than 45% di- and tripeptides, exhibited faster early-phase absorption kinetics, thereby enhancing early-phase bioavailability. Collagen-derived amino acids appeared across the epithelial layer within minutes and reached plasma peaks earlier, compared to a standard collagen hydrolysate. These findings suggest that LMWCP exhibits distinct, accelerated absorption kinetics compared to conventional collagen hydrolysates.

## Introduction

Collagen is defined as the most abundant extracellular protein in the human body that mostly assembles into cross-linked striated fibrils. It supports cellular growth and provides mechanical strength to the body’s connective tissues (Sorushanova et al., 2019). Collagen is present in various forms across numerous tissues. It has a unique triple-helix structure composed of repeating Gly-X-Y sequences, where glycine (Gly) is found at every third position, proline (Pro) is frequently in the X position, and hydroxyproline (Hyp) is often in the Y position (Ramshaw, Shah & Brodsky, 1998). Collagen peptides are diversely used in food, beverages, cosmetics, pharmaceuticals, and biomedical applications (Meena, Mengi & Deshpande, 1999; Musayeva, Özcan & Kaynak, 2022; Wosicka-Frackowiak et al., 2024).

Collagen exerts its potentially beneficial effects when it is optimally digested, and the smallest units, like amino acids (AA), dipeptides, and tripeptides, are absorbed through the digestive system. The digestibility of proteins may vary depending on the source and method of processing. Hydrolysis is one of the processing methods used to enhance protein digestion, absorption, and the postprandial plasma AA profile (Koopman et al., 2009).

Hydroxyproline-containing di- and tripeptides, such as Pro-Hyp and Gly-Pro-Hyp, have attracted considerable interest because these selected intact peptides have been detected after oral collagen ingestion and have been investigated in prior mechanistic studies (Hao et al., 2020; Heres, Mora & Toldrá, 2023; Iwai et al., 2005; Skov et al., 2019; Song, Tian & Li, 2020). However, comparative human data directly linking molecular-weight distribution and peptide composition to early-phase intestinal handling and systemic amino acid appearance remain limited.

The research on the bioavailability of specific collagen peptides is predominantly conducted in animal models. This is primarily attributable to the limitations on conducting detailed mechanistic studies and the costly, time-consuming nature of human-subject research (Sontakke et al., 2016; Wang et al., 2015). Interspecies differences in the digestive system, intestinal permeability, and metabolic activity preclude robust predictions of human collagen peptide absorption using animal models (Punt et al., 2017; Virgilio et al., 2024). To overcome interspecies differences, intestinal cell culture models are increasingly preferred over animal models for assessing intestinal transport of short-chain collagen peptides and amino acids (Álvarez-Olguín et al., 2022).

The human epithelial Caco-2 cell culture model is a widely used *in vitro* model for assessing intestinal AA transport over very short durations (less than 15 min) and direct cellular uptake (Feng & Betti, 2017). It is a well-characterized approach for estimating protein absorption kinetics; therefore, the results are consistent with those reported by Feng & Betti (2017) whose study demonstrated that the molecular weight profile significantly affects protein absorption. The results obtained from the Caco-2 cell monolayer model directly confirmed that the transport efficiency of free amino acids, di- and tripeptides is proportional to the fraction of compounds with a molecular weight below 2,000 Da. In particular, hydroxyproline, as a hallmark AA for collagen, is frequently used to quantify collagen tansport efficiency across Caco-2 monolayers. Accordingly, the objective of this study was to compare the digestion and absorption kinetics of an ultra-low-molecular-weight collagen preparation rich in di- and tripeptides (LMWCP; 500 Da) with those of a market-standard collagen hydrolysate (CP; 2,000–3,000 Da) using product characterization, a Caco-2 transport model, and an exploratory human postprandial plasma study.

## Materials & Methods

### Study overview

**Figure 1:**
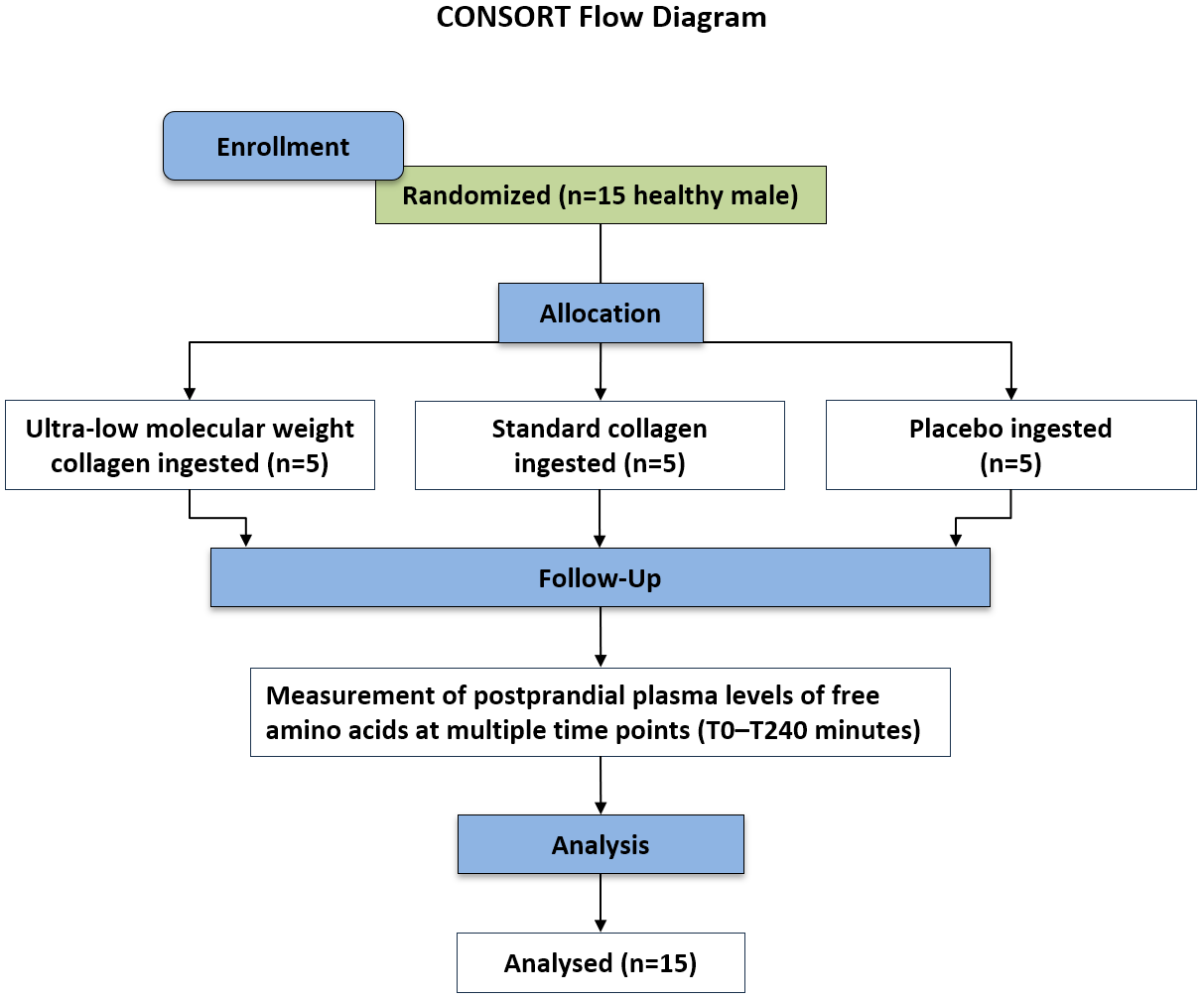
Consort diagram of the trial.

Before the transport and human experiments, both test products were characterized for free amino acid content and selected hydroxyproline-containing di- and tripeptides. An exploratory pilot, single-blind, parallel-group human study involving 15 healthy male participants (LMWCP, CP, and placebo; *n* = 5 per group) measured postprandial plasma free amino acids kinetics over 240 min. A CONSORT-style overview of the trial is provided in Fig. 1.

## Product characterization

Collagen-derived free amino acids and peptides were quantified (Waters, 2020) by ultra-performance liquid chromatography coupled with tandem mass spectrometry (UPLC-MS/MS) from a collagen product containing > 45% di- and tripeptides (LMWCP: SOLUGEL(r) 500 Da, PBLeiner, part of Tessenderlo Group, Belgium) and from market-standard collagen peptides (CP: SOLUGEL(r) 2,000–3,000 Da, PBLeiner, part of Tessenderlo Group, Belgium).

### Quantification of collagen-derived free AAs

Quantification of free AAs was performed following the Waters AccQTag™ method using the AccQTag™ Ultra derivatization reagent kit (Waters Corp., Waters, MA, USA). Chromatographic separation was performed using the CORTECS C18 chromatographic column (2.1 × 150 mm × 1.6 μm) in an Agilent UPLC-MS/MS 6460 system (Agilent Technologies, Inc., Carpinteria, CA, USA). The mobile phases used were H_2_O/formic acid (0.1%, Sigma, St. Louis, MO, USA) and ACN/formic acid (0.1%, Sigma, St. Louis, MO, USA). The flow rate was 0.5 mL/min, the column temperature was 40 °C, and the total run time was 9 min.

### Quantification of collagen-derived peptides

Collagen-specific hydroxyproline-containing di- and tripeptide sequences (Pro-Hyp, Hyp-Gly, Ala-Hyp, Gly-Pro, Gly-Pro-Hyp, Pro-Hyp-Gly, Ala-Hyp-Gly, and Ser-Hyp-Gly), which are known to be absorbed intact into the bloodstream, were selected based on reports in scientific literature. The total amount of these peptides was quantified with a protocol based on the Waters method using the AB Sciex UHPLC-MS 6500+ (Heidenreich et al., 2021). Chromatographic separation was performed on an Agilent InfinityLab Poroshell Z-HILIC column (100 × 2.1 mm, 2.7 µm; Agilent Technologies Inc., Carpinteria, CA, USA) using water (20 mM ammonium formate, pH 3) and acetonitrile (ACN; 90/10 or 10/90; Sigma, St. Louis, MO, USA) as mobile phases.

### *In vitro* digestion and Caco-2 cell model experiments

Products underwent *in vitro* digestion following the guidelines published by Minekus et al. (2014) including oral (amylase digestion), gastric (pepsin digestion), and small intestinal (intestinal enzyme digestion) phases. Post-digestion samples were used for (i) Caco-2 cell viability testing, (ii) Caco-2 transport experiments, and (iii) molecular-weight stability assessment. For cell viability, Caco-2 cells were seeded at 10,000 cells/well in 96-well plates, exposed after 24 h to digested product samples at selected concentrations of 10, 5, 2, 1, 0.5, 0.25, and 0.125 mg/mL, and evaluated after 24 h using the MTT assay according to ECVAM guideline protocol #17 (Arunachalam & Sreeja, 2025; European Commission and Joint Research Centre, 2019).

The pre- and post-digestion molecular-weight (MW) profiles of CP and LMWCP were also quantified after the indicated standardized *in vitro* digestion procedure to compare how each product’s MW distribution changed during simulated gastrointestinal digestion (Minekus et al., 2014). The stability of peptide ingredients during gastrointestinal transit is critical for maintaining their functional properties. Thus, the molecular weight (MW) stability of two peptide products, CP and LMWCP, was evaluated over a 5-hour incubation using the same standardized *in vitro* digestion model.

### Assessment of the Caco-2 cell monolayer integrity

A dye-exclusion approach was applied using membrane-impermeable tracers FITC and 7-AAD to evaluate barrier integrity and exclude paracellular permeability (Faber & McCullough, 2020; Ohkubo et al., 2003). Caco-2 cells were seeded in Transwells (50,000 cells/Transwell). FITC (10 µg/mL) or 7-AAD (10 µg/mL) was added to the apical medium on days 2, 4, 6, 8, 12, 14, 16, and 18 of differentiation. After 240 min of incubation, fluorescence in the basolateral compartment was measured. The use of FITC and 7-AAD as membrane-impermeable tracers in epithelial barrier or membrane integrity assessment has also been described previously (Ito, TD & Modiano, 2010; Moumaris, Rajoelya & Abuafa, 2015; Yao et al., 2017). Because these tracers do not cross an intact epithelial monolayer, the absence of a detectable basolateral signal indicated complete monolayer confluence and preserved barrier integrity under the experimental conditions.

### Caco-2 cell transepithelial transport model

Following *in vitro* digestion, product samples (five mg/mL) were added to the apical compartment of differentiated Caco-2 cell monolayers; placebo controls contained the digestion buffer without product. Basolateral samples were collected at 0, 5, 10, 15, 30, 120, and 240 min for quantification of permeated free amino acids using the method described above. These measurements reflect free amino acids, and do not establish sequence-specific peptide transport.

### Human study

A randomized, single-blind, parallel-group human trial evaluated orally ingested LMWCP, CP, and placebo. Randomization was performed using a computer-generated allocation sequence, with participants assigned to the three intervention groups in a 1:1:1 ratio. Fifteen healthy male volunteers were assigned to three groups (1:1:1; *n* = 5 per group) and attended a single study visit. A parallel-group design was used to avoid carry-over effects associated with repeated collagen ingestion. A dose of 400 mg/kg body weight was administered to enable detection of short-term postprandial kinetic differences. This dose was selected to ensure sufficiently high circulating protein concentrations for accurate detection and quantification, rather than to model habitual intake, consistent with other acute kinetic studies (Guo et al., 2018). After a 12-hour fast, volunteers ingested either collagen (400 mg/kg, dissolved in 200 mL water) or a placebo (200 mL water). Blood samples were collected by finger prick (VeriFine safety lancets, 21G) at 0, 15, 30, 120, and 240 min post-administration in EDTA tubes (Minicollect tube K3E EDTA) and then centrifuged at 1,000 rpm for 5 min at 4 °C. The plasma was stored at −20 °C for further analysis.

Inclusion criteria: Male, age 25–45 years, weight 60–80 kg, BMI: 18.5–29.9 kg/m^2^, signed informed consent. Exclusion criteria: allergies to gelatin, red meat (Alpha Gal Syndrome) or fish, collagen metabolism disorders (*e.g.*, Goodpasture syndrome, scleroderma, periarteritis nodosa, dermatomyositis, disseminated lupus erythematosus), chronic gastrointestinal tract diseases (*e.g.*, peptic ulcer, duodenal ulcer, chronic atrophic gastritis, helicobacter pylori, chronic peptic disorders, chronic acid reflux, Crohn’s disease, irritable bowel syndrome, lactose intolerance, diverticulitis, diverticulosis, cardiovascular diseases, chronic kidney disease, endocrinological disorder, metabolic disease, a history of alcohol or drug abuse, mental illness, a history of asthma or autoimmune disease, a history of smoking within the past year, current or previous intake of hormones, obesity drugs, absorption inhibitors, antidepressants or appetite suppressors, use of oral hormone therapies (*e.g.*, cortisone or steroids) in the six months prior to initiation of this study, having abnormal liver or renal function tests, having blood pressure >140/90 mmHg or hypertension requiring a prescribed diuretic, having participated in another clinical trial within the past six months, having donated blood within the past month, having abnormal findings in blood test results as determined by a specialist, and any condition judged by the investigator to be unsuitable for participation in the study. Volunteers were required to adhere to the product’s conditions of use, refrain from consuming other food supplements during the study period, avoid introducing new nutricosmetic products into their daily routine, maintain their usual dietary habits, and abstain from drugs or dietary supplements intended for weight reduction or modulation of gastrointestinal metabolic parameters. To minimize dietary variability, participants completed a standardized 12-hour overnight fast prior to product administration and were instructed to maintain their habitual diet while refraining from collagen-containing supplements, nutri-cosmetic products, alcohol consumption, and major dietary modifications during the study period.

### Sample size justification

The human intervention component of this study was designed as an exploratory pilot study to characterize short-term postprandial plasma amino-acid kinetics rather than to evaluate clinical outcomes. Fifteen healthy male volunteers were allocated to three parallel groups (LMWCP, CP, and placebo), with five participants per group. While a formal a priori statistical power calculation was not the primary focus at the design stage, the human component was intentionally structured as an exploratory pilot study. The emphasis was placed on characterizing short-term kinetic patterns, with the aim of generating meaningful physiological insights rather than establishing definitive estimates of clinical outcomes. Plasma amino acid concentrations were measured repeatedly at 0, 15, 30, 120, and 240 min after product ingestion, enabling within-participant kinetic profiling across the postprandial period. Given the exploratory nature of the study, the repeated-measure structure of the primary endpoint, and the intensive sampling protocol, the sample size was considered appropriate for an initial comparative assessment of amino-acid absorption kinetics under controlled conditions.

### Statistical analysis

The MTT assay was performed with eight replicates per condition, and the other quantification assays with two replicates per condition. Data outliers were identified using the ROUT method (Q = 5%) and, if present, excluded from the analysis. Data were statistically analyzed by one-way ANOVA test and Holm-Sidak’s or Dunnett’s T3 *post hoc* multiple comparisons test. Statistical significance was declared at *p* < 0.05, with a 95% confidence interval. Where applicable, exact *p*-values are reported in the Results and tables. Bars in the charts represent the mean value for each condition, and error bars indicate the standard error of the mean (SEM) for each group of values. For the human kinetic study, repeated-measures analyses were used to assess temporal changes in plasma amino acid concentrations across postprandial time points and to compare time-course patterns between groups.

### Ethical considerations

The human study protocol is based on the guidance of the Scientific Committee on Consumer Safety (SCCS). It meets all international standards for research studies involving human subjects, including the International Conference on Harmonization’s Good Clinical Practices (ICH-GCP) and the World Medical Association’s Declaration of Helsinki. It has been conducted under the Declaration of Helsinki (1964), with the amendments of Tokyo (1975), Venice (1983), Hong Kong (1989), Somerset West (1996), Edinburgh (2000), Washington (2002), Tokyo (2004), Seoul (2008), and Fortaleza (2013). The standard protocol and test conditions were submitted to and approved by the Ethical Committee of Hospital Universitario y Politécnico La Fe (Date: 2022/11/30, Code number: 2022-920-1). This study was registered at ClinicalTrials.gov (ClinicalTrials.gov Identifier: NCT05722158, February 2023) (https://clinicaltrials.gov/study/NCT05722158).

## Results

### Peptides and amino acid contents of collagen hydrolysates

### Free amino acids in collagen hydrolysates

The CP and LMWCP products showed distinct unbound free-amino acid profiles (Table 1). The quantified free amino acid composition was expressed in µg of amino acid per gram of collagen product. Thirty-three free amino acids and post-translationally modified forms were detected. The total free amino acid content in CP was 3,351.01 µg/g, and in LMWCP, it was 36,696.37 µg/g.

**Table 1:** Quantification of free amino acids and their post-translational modified forms in tested collagen hydrolysates.

	**CP**	**LMWCP**
	**Mean ± SD.**	**Mean ± SD.**
**1-Methylhistidine” or 3-Methylhistidine**	1.85 ± 1.8	0.01 ± 0.01
**Alanine**	349.08 ± 18.8	874.34 ± 61.74
**Alpha Aminobutyric acid**	4.64 ± 0.5	20.79 ± 20.79
**Aminoadipic acid**	5.9 ± 0.5	950.14 ± 794.08
**Anserine**	1.2 ± 1.2	nd
**Arginine**	102.19 ± 14.9	16,030.31 ± 1,326.89
**Asparagine**	79.93 ± 11.1	312.79 ± 45.26
**Aspartic acid**	27.5 ± 2.3	1,174.49 ± 936.01
**β-Alanine**	348.78 ± 18.7	877.68 ± 63.8
**β-Aminoisobutyric acid**	4.81 ± 0.6	14 ± 14
**Carnosine**	nd	152.18 ± 76.28
**Citrulline**	372.59 ± 31.5	122.64 ± 122.64
**Cystathionine**	15.55 ± 15.5	nd
**Cystine**	4.03 ± 4	nd
**Ethanolamine**	0.61 ± 0.3	8.09 ± 7.94
**γ-Aminobutyric acid (GABA)**	4.98 ± 0.7	20.53 ± 19.5
**Glutamic acid**	125.06 ± 4.4	2,627.86 ± 970.53
**Glycine**	632.21 ± 37.1	3,629.38 ± 3102.35
**Histidine**	97.22 ± 97.2	82.87 ± 8.78
**Homocysteine**	nd	1.91 ± 1.91
**Hydroxylysine**	17.02 ± 0.4	118.93 ± 10.83
**Hydroxyproline**	68.18 ± 5.8	12.89 ± 0.89
**Isoleucine + Leucine**	202.44 ± 12.7	5,632.65 ± 604.91
**Ornithine**	142.95 ± 11.6	13.76 ± 1.05
**Phenylalanine**	170.14 ± 14.8	2,116.08 ± 134.39
**Phosphoethanolamine**	0.09 ± 0.09	0.86 ± 0.8
**Phosphoserine**	nd	nd
**Proline**	189 ± 15.3	38.76 ± 1.83
**Sarcosine**	348.93 ± 18.7	875.23 ± 62.35
**Serine**	16.63 ± 2.4	356.43 ± 192.44
**Taurine**	1.66 ± 1.6	6.25 ± 6.25
**Threonine**	13.69 ± 1.8	513.66 ± 221.6
**Tyrosine**	1.81 ± 1.8	101.16 ± 101.16
**Total**	3,351.01	36,696.37

**Notes.**

Expressed as -μg amino acid per g product. Data are presented as mean ± SD. ND, Not detectable (absence of signal). CP, market-standard collagen hydrolysate; LMWCP, ultra-low molecular weight collagen.

In LMWCP, the most abundant free amino acids were arginine (16,030.31 ± 1,326.89 μg/g), isoleucine + leucine (5,632 ± 604.91 μg/g), glycine (3,629.38 ± 3,102.35 μg/g), and glutamic acid (2,627.86 ± 970.53 μg/g).

The amount of collagen-derived free amino acids (glycine, hydroxyproline, proline, and hydroxylysine) differed markedly between the two products (Table 1). LMWCP contained substantially higher levels of glycine and hydroxylysine than CP. In contrast, CP showed higher hydroxyproline and proline contents, showing clear compositional differences between the formulations.

### Selected hydroxyproline-containing peptides in collagen hydrolysates

LMWCP formulation was selected for peptide quantification due to its ultra-low molecular weight profile relative to CP and it controlled enzymatic hydrolysis process. By focusing on LMWCP, which is rich in absorbable amino acids and low-molecular-weight peptides, this approach allowed targeted assessment of selected functionally relevant peptides. Quantitative AA analysis revealed that the LMWCP contained the highest levels of key collagen-derived amino acids—glycine, proline, hydroxyproline, and hydroxylysine. This biochemical profile supports further evaluation of LMWCP as a source of collagen-derived peptides. The total amount of selected hydroxyproline-containing di- and tripeptides (Pro-Hyp, Hyp-Gly, Ala-Hyp, Gly-Pro, Gly-Pro-Hyp, Pro-Hyp-Gly, Ala-Hyp-Gly, Ser-Hyp-Gly) is >5,000 ppm, which is higher than that noted in the literature for low molecular weight collagen peptides (León-López et al., 2019; Oztug, 2024).

### Molecular-weight stability during *in vitro* digestion

The MW stability of CP and LMWCP was assessed over the 5-hour standardized *in vitro* digestion procedure. CP showed pronounced susceptibility to enzymatic breakdown, with a 49% reduction in MW after digestion, whereas LMWCP showed only a 4% decrease, indicating greater stability profile degradation and preserve original content of the low-molecular-weight formulation under the applied digestive conditions.

#### *In vitro* cell culture study

##### *In vitro* Caco-2 cell viability

**Figure 2:**
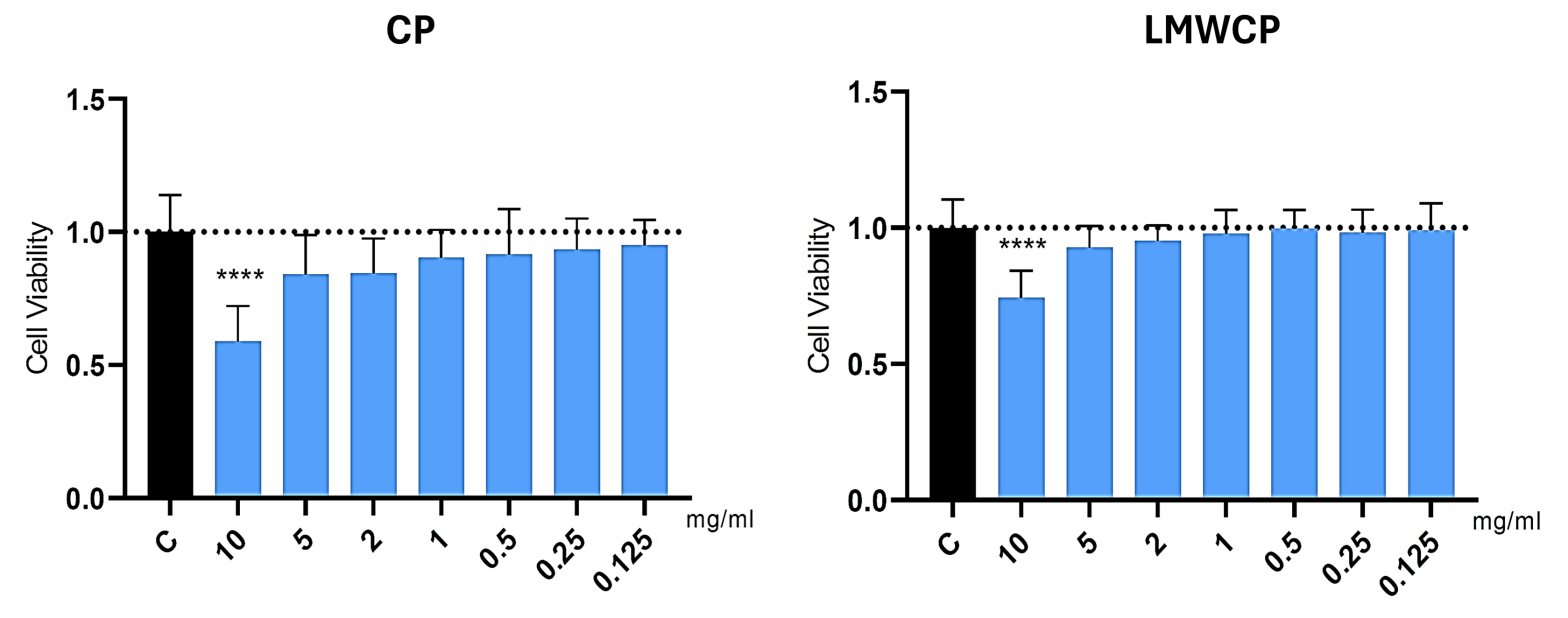
Graphical representation of the results showing the cell viability of Caco-2 cells after treatment with the digested products at the indicated concentrations for 24 h, compared with the untreated control. CP, market-standard collagen hydrolysate; LMWCP, ultra-low molecular weight collagen. Asterisks denote statistical significance as follows: *p < 0.05, **p < 0.01, and ****p < 0.0001.

To assess the biocompatibility of the collagen-based products, Caco-2 cells were exposed to increasing concentrations (0.125–10 mg/mL) of digested product samples (CP and LMWCP) for 24 hours, and cell viability was evaluated (Fig. 2). A clear dose-dependent pattern was revealed at the highest concentrations. Both CP and LMWCP produced a statistically significant reduction in cell viability at 10 mg/mL (****p < 0.0001) compared with untreated control cells. However, at all lower concentrations, neither CP nor LMWCP caused significant cytotoxicity, and cell viability remained comparable to that of the control. We observed no notable differences in the viability profiles of the two products, indicating similar *in vitro* safety margins. These findings suggest that both CP and LMWCP are well tolerated by the cells at concentrations up to 5 mg/mL, with mild toxicity only emerging at supraphysiological levels (10 mg/mL).

### Assessment of Caco-2 cell monolayer integrity

To evaluate the formation and integrity of the Caco-2 epithelial monolayer over time, a fluorescence-based permeability assay was conducted using dye diffusion across Transwell inserts. The fluorescence intensity in the basolateral medium, expressed as relative fluorescence units (RFU), inversely reflected the integrity of the cell barrier—higher RFU values indicated greater permeability.

**Figure 3:**
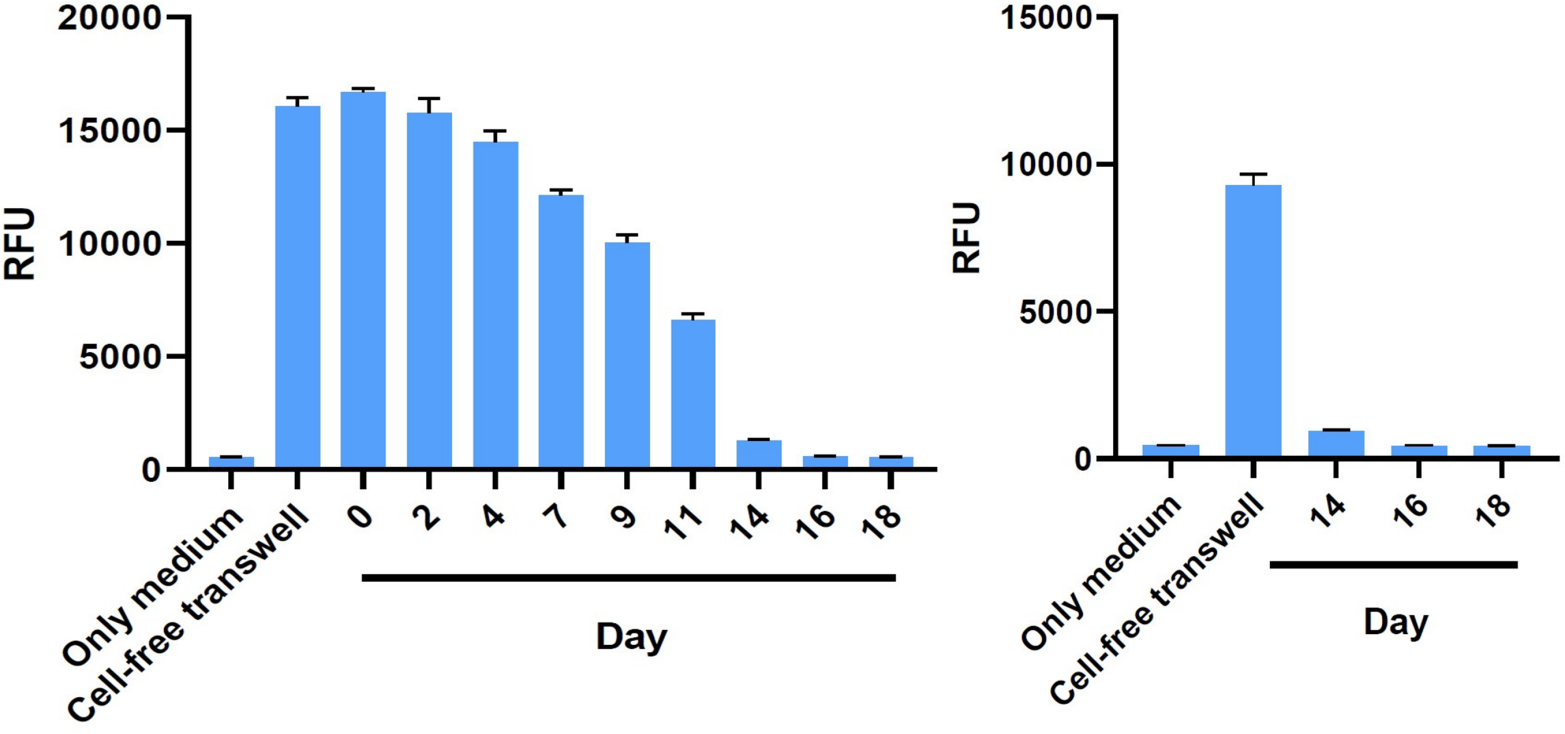
Graphical representation of dye (FITC or 7-AAD) detected in the basolateral medium as an indicator of monolayer integrity. “Only medium” refers to fluorescence measured in culture medium alone, while “Cell-free transwell” refers to fluorescence in the basolateral medium of a transwell without cells.

As illustrated in Fig. 3, at Day 0, RFU levels were similar to those in the cell-free Transwell condition (∼16,000 RFU), consistent with maximum permeability and the absence of a functional monolayer. Between Day 2 and Day 11, RFU values gradually declined, which corresponds with the formation of tight junctions. By Day 14, the RFU measurements decreased to near-baseline levels (∼1,500 RFU), comparable to the “only medium” background control. This low level of dye diffusion persisted through Day 16 and Day 18, confirming the establishment of a mature and stable epithelial barrier.

Together, these findings indicate that the Caco-2 cell monolayer reached a stable barrier phenotype by Day 14, and maintained integrity through Day 18, supporting the use of this model for comparative transport experiments under the study conditions.

### Transepithelial amino acid appearance

The trans-epithelial appearance of amino acids derived from digested LMWCP and CP was evaluated over 240 min using the Caco-2 model. Quantification of free amino acids in the basolateral compartment showed earlier appearance of several collagen-derived amino acids with LMWCP than with CP (Tables 2 and 3).

**Table 2:** Time-dependent free amino acid levels in the basolateral compartment of the Caco-2 cell Transwells for ultra-low molecular weight collagen (LMWCP).

**Amino acid (mg/L)**	Ultra-low molecular weight collagen (LMWCP)						
	**T0 min**	**T5 min**	**T10 min**	**T15 min**	**T30 min**	**T120 min**	**T240 min**
	**mean ± SEM**	**mean ± SEM**	**mean ± SEM**	**mean ± SEM**	**mean ± SEM**	**mean ± SEM**	**mean ± SEM**
**Aspartic Acid**	14.5 ± 1.8	14.6 ± 0.5	13.4 ± 0.9	13.1 ± 3.1	14.5 ± 0.1	13.4 ± 0.3	11.2 ± 0.4
**Glutamic Acid**	43 ± 1.2	43.7 ± 0.7	41.6 ± 2	43.3 ± 6.7	45.3 ± 0.9	42.1 ± 0.8	37.6 ± 0.7
**Alanine**	20.9 ± 0.4	21 ± 0	21.6 ± 0.3	24.8 ± 1^s^	23.1 ± 0.7 ^ ns^	24.1 ± 1.8^s^	24.6 ± 1^s^
**Arginine**	92.8 ± 1.9	96.5 ± 0.4	93.3 ± 2.2	101.4 ± 6.2	94.3 ± 11.2	99.9 ± 0.8	96.5 ± 7.6
**Asparagine**	10.2 ± 0	10.4 ± 0.6	10.3 ± 0.9	11.4 ± 0.1^ns^	10.1 ± 0.8	10.8 ± 0	9.8 ± 0.2
**Cystine or Cysteine**	16 ± 1	16.6 ± 0.3	14.8 ± 0.6	15.9 ± 1.3	16.6 ± 0.3	15.9 ± 0.3	15.2 ± 0.4
**Phenylalanine**	35.9 ± 1.6	38.1 ± 0	34.4 ± 1.9	39.5 ± 1.4^ns^	39.5 ± 0.3^ns^	39.1 ± 1.6	38 ± 0.4
**Glycine**	16.1 ± 1.7	14.9 ± 0	14 ± 3.6	12.5 ± 0.6	17.4 ± 3.2	13.7 ± 0	15.4 ± 0.9
**Glutamine**	611.2 ± 36.1	643.5 ± 18.7	579.2 ± 15.2	658 ± 24.2	595.6 ± 63.1	662 ± 37.4	616.2 ± 10.7
**Isoleucine**	56.6 ± 0.8	57.3 ± 1	54.1 ± 0.6	60.6 ± 2.2^ns^	60.3 ± 0.7^ns^	58.3 ± 1.7	58 ± 1.7
**Histidine**	33.3 ± 0.3	34.7 ± 0.2	32.6 ± 0	36.6 ± 1.9^ns^	35.8 ± 0.3	35.7 ± 1.5	34.1 ± 0.4
**Leucine**	56.3 ± 1.6	56.9 ± 0	52.8 ± 1.4	60 ± 3	59.3 ± 0.6	57.3 ± 2.8	56.5 ± 1.5
**Lysine + Hydroxylysine**	49.1 ± 1.9	50.5 ± 2.2	48 ± 0.4	54.6 ± 1.7^ns^	53.2 ± 0.1	52 ± 2.4	51.8 ± 1.4
**Methionine**	13.9 ± 0.9	13.5 ± 0.4	13.7 ± 0.7	16.4 ± 0.4^ns^	16.1 ± 0.5^ns^	14 ± 0.7	14.4 ± 0.5
**Proline + Hydroxyproline**	15.4 ± 0.1	16.2 ± 0.4	14.1 ± 1.5	15.4 ± 0.6	14.7 ± 0	14.1 ± 0.4	16.7 ± 0.5
**Serine**	18.9 ± 0.5	18.7 ± 0.6	17.9 ± 1.5	19.2 ± 0.2	19.4 ± 0.3	18.7 ± 0.5	18 ± 1
**Tyrosine**	39.9 ± 2.8	42 ± 0.2	40.2 ± 2	48.8 ± 0.5^s^	47.6 ± 0.4^s^	44.6 ± 1^ns^	45.7 ± 0.2^s^
**Threonine**	45.6 ± 1.3	45.9 ± 0.7	45.1 ± 1	51.2 ± 1.3^s^	49.4 ± 0	48 ± 2	48.3 ± 1.4
**Valine**	54.4 ± 0.7	55.1 ± 1.2	51.9 ± 0.4	59 ± 2.7^ns^	58.8 ± 1.3^ns^	56.6 ± 1.1	55.1 ± 0.8
**Ornithine**	8.5 ± 1.8	7.1 ± 0.5	14.4 ± 0.4^s^	14.2 ± 0.9^s^	14.9 ± 0.1^s^	12.3 ± 2.7	10.2 ± 0.7
**Total**	1,245.0	1,291	1,194	1,342.9	1,271.9	1,321.4	1,264.1

**Notes.**

One-way ANOVA followed by Dunnett’s multiple comparisons *post hoc* test was performed

Data are represented in mg/L (mean ± SEM). Superscript ‘s’ denotes a statistically significant difference (*p* < 0.05) compared with the corresponding basal level (T0). Superscript ‘as’ denotes an approximate significance (0.05 <*p* < 0.1) compared with the corresponding basal level (T0). ND: not detected.

Data are expressed as mean ± SEM.

**Table 3:** Time-dependent free amino acid levels in the basolateral compartment of the Caco-2 cell Transwells for market-standard collagen hydrolysate (CP).

**Amino acid (mg/L)**	Market-standard collagen hydrolysate (CP)						
	**T0 min**	**T5 min**	**T10 min**	**T15 min**	**T30 min**	**T120 min**	**T240 min**
	**mean ± SEM**	**mean ± SEM**	**mean ± SEM**	**mean ± SEM**	**mean ± SEM**	**mean ± SEM**	**mean ± SEM**
**Aspartic Acid**	12.2 ± 2.2	16.8 ± 1.7^ns^	15.2 ± 1.1	14.3 ± 0.2	14.6 ± 1.8	13.9 ± 0.2	13.5 ± 1.1
**Glutamic Acid**	37.7 ± 6.9	51.1 ± 7.5^ns^	44 ± 2	46.5 ± 0.9	42.3 ± 4.3	42.5 ± 0.5	41.3 ± 2.4
**Alanine**	16.8 ± 1.9	21.3 ± 5.2	17.1 ± 0	17.2 ± 0.2	24.2 ± 0.6^ns^	24.3 ± 3.1^ns^	25 ± 0.9^s^
**Arginine**	83.7 ± 7.6	82.6 ± 10.8	84 ± 6.2	88 ± 13.7	88.6 ± 5.1	97.6 ± 4.2	84.2 ± 16.7
**Asparagine**	10.4 ± 1.2	12.6 ± 0.9	11.4 ± 1	11.7 ± 0.7	12.1 ± 0.6	12.3 ± 0.1	11.8 ± 0.8
**Cystine or Cysteine**	14.5 ± 2.4	21.2 ± 6.5	16.1 ± 1.2	16.8 ± 0.1	18.9 ± 1.4	18.7 ± 1.2	18.7 ± 1.3
**Phenylalanine**	34.5 ± 6.1	46.4 ± 11	37.7 ± 2.7	40 ± 0.5	39.7 ± 2	40.2 ± 1.4	40.6 ± 0.4
**Glycine**	16.2 ± 3.4	25.4 ± 5.1	16.1 ± 1.6	19.7 ± 0.1	23.7 ± 1.9	21 ± 3.7	19 ± 5
**Glutamine**	572.5 ± 10.9	495.9 ± 3.4	557.2 ± 37.1	574.4 ± 90.9	581.6 ± 33.5	612.1 ± 20.2	542.5 ± 104
**Isoleucine**	48 ± 8.2	63 ± 12.7	52.8 ± 2.8	54.4 ± 0.5	66 ± 1.8^ns^	66.4 ± 1.6^ns^	64.8 ± 5.1
**Histidine**	29.2 ± 5	37.8 ± 6.9	33.1 ± 2.1	34.2 ± 0.1	41.1 ± 2.3^ns^	41.1 ± 0.8^ns^	40.3 ± 1.7^ns^
**Leucine**	47 ± 8.2	62.3 ± 11.7	53 ± 4	54.8 ± 0.1	66.5 ± 2.7^ns^	65.8 ± 1^ns^	63.9 ± 5.2
**Lysine + Hydroxylysine**	45 ± 7	59.6 ± 13.3	49.1 ± 4.3	51.2 ± 0.4	65.9 ± 3.1^ns^	67.7 ± 1.2^ns^	61.6 ± 10.7
**Methionine**	12.5 ± 1.9	15.6 ± 3.5	13.2 ± 0.9	14.4 ± 0.1	14.1 ± 0.9	14.8 ± 0	15.5 ± 0.7
**Proline + Hydroxyproline**	18.5 ± 1.8	23.7 ± 7.1	18.6 ± 1.2	19.2 ± 0.1	16.3 ± 0.1	16.1 ± 0.1	17.1 ± 0.4
**Serine**	15.7 ± 2.4	20.7 ± 2.4	17.4 ± 0.9	18.4 ± 0.7	21.4 ± 1.1^s^	22.7 ± 0.3^s^	21.2 ± 1.2^ns^
**Tyrosine**	39.5 ± 3.4	50.7 ± 12.3	41.3 ± 2.3	44.4 ± 1.3	45.7 ± 3.4	46.6 ± 1.1	47.7 ± 2.1
**Threonine**	42.5 ± 6.4	53.2 ± 9.2	44.9 ± 4.6	48 ± 0.4	54.2 ± 4.1	55.6 ± 1.8	54.3 ± 5.7
**Valine**	45.1 ± 6.1	58.9 ± 10.3	50.2 ± 3.4	52.4 ± 0.9	68 ± 4.7^s^	70.5 ± 1.1^s^	66.2 ± 7.6^s^
**Ornithine**	9.9 ± 4.2	13.3 ± 6.2	10.8 ± 1.9	10.2 ± 0.5	9.9 ± 0.2	9.8 ± 0.6	10.3 ± 0.1
**Total**	1,142.5	1,219.8	1,173.3	1,220	1,305.8	1,350.9	1,250.3

**Notes.**

One-way ANOVA followed by Dunnett’s multiple comparisons *post hoc* test was performed

Data are represented in mg/L (mean ± SEM). Superscript ‘s’ denotes a statistically significant difference (*p* < 0.05) compared with the corresponding basal level (T0). Superscript ‘as’ indicates an approximate significance (0.05 <*p* < 0.1) compared with the corresponding basal level (T0). ND: not detected.

Data are expressed as mean ± SEM.

In the LMWCP group, several amino acids, including asparagine, glutamic acid, and isoleucine, increased early and remained elevated between 15 and 30 min. This pattern is consistent with faster early-phase appearance of digestion products in the basolateral compartment.

**Figure 4:**
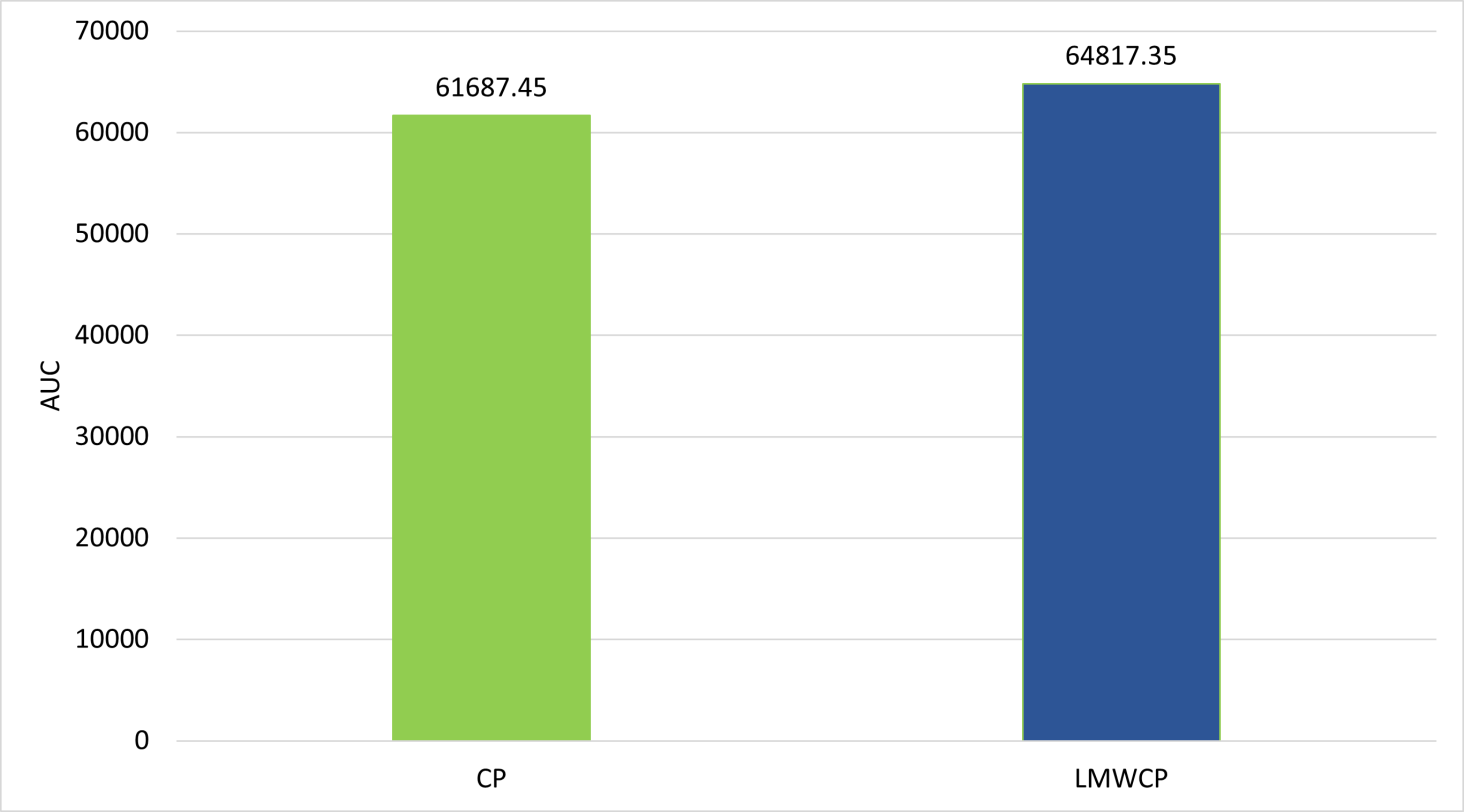
Area under the curve (AUC) for basolateral transport levels of collagen-derived amino acids during 0–30 minutes in Caco-2 monolayers exposed to LMWCP *versus* CP. CP, market-standard collagen hydrolysate; LMWCP, ultra-low molecular weight collagen.

By contrast, the CP group showed a later pattern, with several significant increases emerging mainly at 120 or 240 min (Tables 2 and 3). Analysis of the area under the curve (AUC) (Fig. 4) revealed that LMWCP showed greater absorption within the first 30 min post-administration than standard collagen, indicating a more rapid absorption kinetics of LMWCP during the early phase. Overall, these findings support that the LMWCP exhibits a faster early-phase absorption-kinetic profile compared with standard collagen hydrolysate formulations under the experimental conditions.

### Human plasma amino acid kinetics

Plasma concentrations of key collagen-derived amino acids—glycine, proline, and hydroxyproline—were monitored over 4 h after oral intake. The study was designed to characterize postprandial kinetic behavior. A total of 23 different amino acids were detected in human plasma. After ingestion of collagen peptides dissolved in water, plasma glycine, proline, and hydroxyproline increased in both collagen groups relative to baseline, but the temporal profiles differed between groups. LMWCP showed earlier peaks, whereas CP reached its highest value later.

**Figure 5:**
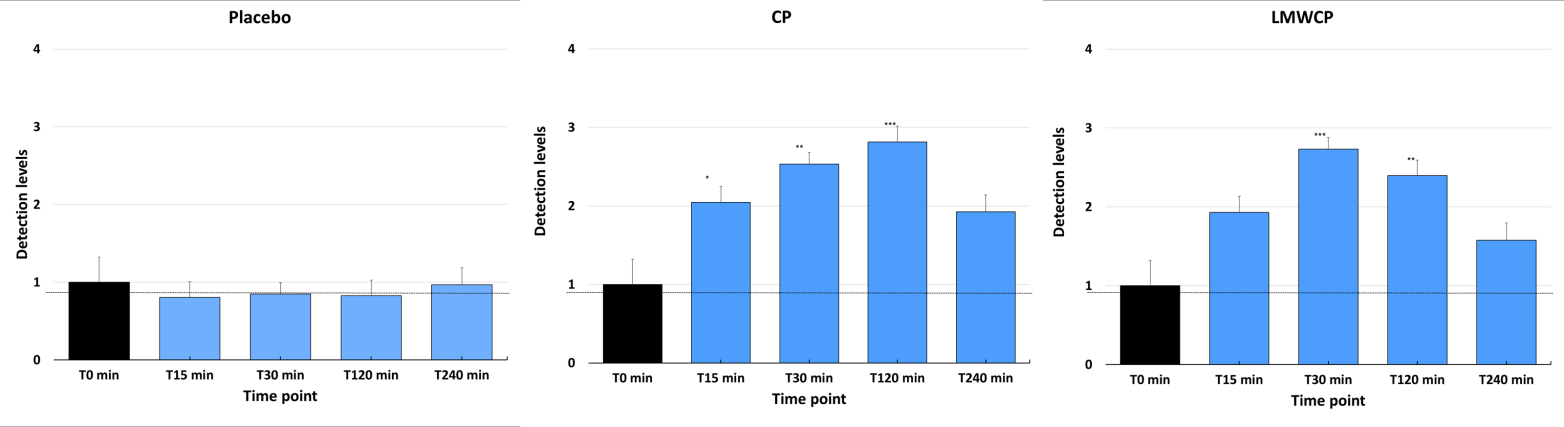
Normalized plasma levels of proline, hydroxyproline, and glycine in the human blood plasma at the assessed time points following oral intake of the products. T0 indicates amino acid levels in plasma prior to intake (*i.e.*, basal levels). Results are normalized to T0 (basal level). CP, market-standard collagen hydrolysate; LMWCP, ultra-low molecular weight collagen. Asterisks indicate significant differences compared with T0. * *p* < 0.05, ** *p* < 0.01, *** *p* < 0.001.

Taken together, the *in vitro* and *in vivo* datasets indicate faster early-phase appearance of collagen-derived amino acids with LMWCP. Under the present experimental conditions, CP showed a comparatively delayed profile (Fig. 5). These findings highlight the superior intestinal permeability of the LMWCP formulation in the first 30 min, which are likely attributable to its distinct composition.

## Discussion

This study compared the absorption kinetics of an ultra-low molecular weight collagen peptide formulation containing di- and tripeptides (LMWCP; 500 Da) with those of a market-standard collagen hydrolysate (2,000–3,000 Da) using product characterization, *in vitro* digestion followed by a Caco-2 cell model, and an exploratory human postprandial plasma study. Across these assays, LMWCP showed faster early-phase appearance of collagen-derived amino acids than CP. Accordingly, these findings provide a detailed profile of the digestion and absorption kinetics of collagen peptides with different compositions. By integrating collagen product composition with Caco-2 transport data and human plasma amino acid profiles, the study shows that collagen hydrolysate formulations can differ in early digestion/absorption behavior. However, as the study is primarily designed to characterize digestion and absorption kinetics, it does not include a comprehensive analysis of circulating peptide-specific signaling processes. Further research is needed to address these aspects in more depth.

The *in vitro* experiments revealed differences in the basolateral appearance of collagen-characteristic amino acids—glycine, hydroxyproline, and proline across Caco-2 monolayers. LMWCP showed an earlier signal than CP, which is consistent with faster generation and/or transfer of smallest units of absorbable collagen content. The time-course profiles indicate that amino acid appearance continued over time rather than occurring as a single bolus event. Because the assay quantified basolateral free amino acids, these data reflect net transepithelial nitrogen flux. Since amino acids continue to appear in the basolateral compartment (or the bloodstream *in vivo*) over time, especially beyond the initial peak, this suggests that the compound is not absorbed all at once. Thus, a gradual increase or a plateau indicates a sustained-release mechanism and a degree of hydrolysis that facilitates intestinal passage. While the Caco-2 model is valuable for mechanistic comparisons under controlled conditions, it does not fully replicate the complexity of the human intestine, including mucus layer effects, dynamic luminal digestion, and inter-individual transporter expression. Therefore, mechanistic inferences regarding specific uptake pathways should be interpreted cautiously and viewed as supportive rather than definitive.

The relatively high collagen-derived amino acid content present before the gastric and intestinal phases may also have influenced the post-digestion profile. In this context, the final profile is determined not solely by baseline free amino acids but also by the extent and kinetics of enzymatic hydrolysis during simulated gastrointestinal digestion. Thus, pre-existing free amino acids may partly contribute to the observed post-digestion pattern, while ongoing hydrolysis further shapes the pool of absorbable amino acids and small peptides (Dallas et al., 2017). Accordingly, the Caco-2 findings should be interpreted as reflecting comparative transport behavior under controlled conditions. While the results highlight kinetic differences between the formulations, they also suggest that collagen peptides with ultra low molecular weight to be absorbable may influence absorption processes.

The *in vivo* pharmacokinetic data revealed that oral administration of collagen hydrolysates led to distinct elevations in circulating levels of key collagen-derived AAs, namely glycine, proline, and hydroxyproline. Following ingestion, plasma concentrations of these AAs rose rapidly, reaching peak levels between 30 and 120 min post-administration, then gradually returned toward baseline by 240 min. This pattern indicates efficient intestinal absorption and systemic availability of collagen-derived AAs, consistent with previously reported time-concentration profiles for hydrolyzed collagen in humans. The observed increase in glycine and hydroxyproline, two hallmark AAs of collagen breakdown, reflects highly specific enzymatic hydrolysis of ingested collagen peptides in the gastrointestinal tract and subsequent uptake as free AAs. The differences in magnitude and timing of AA peaks between products likely result from variations in molecular weight distribution and peptide length. Collectively, these findings suggest that the biological effects of hydrolyzed collagen are primarily mediated by the bioavailability and systemic kinetics of its AA components, which may be explained by efficient intestinal transport of LMWCP and by the sustained presence of collagen-derived AAs (and potentially small peptide fragments) in circulation. The earlier postprandial peak observed with LMWCP may be relevant for timing-sensitive applications where rapid early-phase AA availability is desirable. Our results show significant differences in AA transport and digestion and absorption kinetics among products labeled as “collagen hydrolysates”. LMWCP exhibited higher systemic exposure and epithelial permeability, whereas CP showed fewer effects in both models. These discrepancies likely result from differences in the degree of hydrolysis, molecular weight distribution, and content of the collagen peptides, as discussed in prior comparative studies.

These findings underscore that not all collagen peptide preparations are the same. Differences in digestion behavior, absorption kinetics, and peptide length and composition lead to distinct bioavailability profiles across formulations. Given the growing use of collagen supplements, formulation-specific evaluation of digestive and absorptive behavior is therefore essential. Integrating *in vitro* transport assays with short-term human kinetic profiling provides a valuable framework for comparative characterization and product differentiation. Overall, our findings indicate that well-designed, multi-modal studies can play an important role in refining the characterization of collagen hydrolysates and distinguishing their biochemical and kinetic properties.

### Strengths and limitations

This study benefits from a translational design that integrates product characterization and cellular transport behavior in the Caco-2 model with human postprandial amino acid kinetics, providing a coherent comparative assessment of collagen peptide formulations. The human component was exploratory, involving 15 participants under an acute high-dose regimen. As in early-phase kinetic studies, the sample size and dose and inter-individual variability may influence between-group comparisons; therefore, the findings are best interpreted as controlled kinetic observations rather than reflections of habitual intake responses. Future studies incorporating advanced peptide profiling and targeted physiological readouts will further clarify underlying mechanistic pathways.

### Conclusions

Consequently, this study demonstrates that not all collagen hydrolysates are equivalent. An ultra-low molecular weight (500Da) collagen high in di- and tripeptides (>45%) demonstrated enhanced and earlier transport across the intestinal epithelium *in vitro* and an earlier peak plasma concentration of collagen-derived AAs *in vivo* compared with a standard collagen hydrolysate. These results indicate that molecular weight profile and product composition are key determinants of absorption kinetics, emphasizing that collagen hydrolysates should be assessed on their individual biochemical properties rather than treated as bioequivalent.

## Supplemental Information

10.7717/peerj.21398/supp-1 Supplemental Information 1 Blood Serum Data

10.7717/peerj.21398/supp-2 Supplemental Information 2 *In vitro* data T8 and 9

10.7717/peerj.21398/supp-3 Supplemental Information 3 *In vitro* report

10.7717/peerj.21398/supp-4 Supplemental Information 4 *In vivo* report

10.7717/peerj.21398/supp-5 Supplemental Information 5 Protocol

10.7717/peerj.21398/supp-6 Supplemental Information 6 CONSORT checklist

## Additional Information and Declarations

### Data Availability

The following information was supplied regarding data availability: The data is available in the Supplemental Files.
