# Supplementary material for: Enhanced digestion and absorption kinetics of ultra–low molecular weight collagen containing di- and tripeptides: evidence from human and cell models
Source: PeerJ. 2026 Aug 14;14:e21398. doi: 10.7717/peerj.21398 (PMC13480140; doi:10.7717/peerj.21398)
Supplement: Supplementary file 1 [file peerj-14-21398-s003.pdf]

PB Leiner, Part of Tessenderlo Group

IN VITRO EVALUATION OF THE BIOAVAILABILITY OF COLLAGEN FORMULATIONS (ULMWCP versus CP)

DATE – December 20th, 2024

PB Leiner

Marius Duchéstraat 260,

1800 Vilvoorde,

Belgium

VAT: BE 0412 101 728

GOOD CLINICALPRACTICE
CERTIFICACIÓN ISO9001:2015 Nº EC-10595/21
LGAI Technologigcal Center, S.A. (Appus+) Certifies that the Quality Management System of the organization:
BIONOS BIOTECH, S.L.
For the following activites of:
To perform efficacy studies on products from the cosmetic and food industries. To perform biological diagnostic Assays.
Is in accordance with the requirements of the standard ISO 9001:2015
INITIAL CERTIFICATION DATE: 12/02/2021 THIS CERTIFICATE IS VALID UNTIL: 11/02/2027

C´REDIT D'IMPÔT RECHERCHE (CIR)
Direction Générale de la recherse et de l'innovation
DÉCISION D'AGRÉMENT
(Dépenses de recherche)
BIONOS BIOTECH
46026 VALENCIA
Espagne Cet agrément est accordé au titre des années: 2023, 2024, 2025.

INDEX

1 Title ....

2 Introduction...

3 Collagen formulatons tested ....

4 Registration dates ....

5 Platform..

6 Material and Methods ....

7 Results ....

8 References ....

9 Registry and Regulation..

1 Title

In vitro evaluation of the bioavailability of collagen formulations in cells

2 Introduction

Collagen is a fibrous protein highly demanded in biomedical and cosmetic industries due to its benefits on the skin, biocompatibility, bioactivity, and weak immunogenicity (Avila Rodríguez et al., 2018). This biomaterial is composed of repetitive triplets of amino acids glycine (Gly), proline (Pro), and hydroxyproline (Hyp), and it is found to exert beneficial effects on several health benefits. Proteins in their native form such as collagen present low solubility in water due to its high molecular weight of approximately 300 kDa. These natural properties present difficulties in their application to cosmetics and edible products. However, the hydrolyzed collagen, which consists of segmented proteins with low molecular weight (Mw) between 0.3 kDa and 10 kDa, do not have this problem. The use of natural peptides like collagen hydrolysates has been widely utilized due to their excellent biocompatibility, easy biodegradability, and weak antigenicity (Liu et al., 2015). Recent evidence suggests that the consumption of amino-acids and/or peptides abundantly present in collagen may have the capacity to influence the synthesis of new collagen in the body (Al-Atif, 2022). Indeed, it has been found that collagen supplements improve skin moisture, elasticity, and hydration when orally administered (Al-Atif, 2022). Additionally, collagen reduces the wrinkling and roughness of the skin, and existing studies have not found any side effects of its oral supplements (Al-Atif, 2022).

Simulated digestion methods typically include the oral (amylase digestion), gastric (pepsin digestion) and small intestinal (intestinal enzymes digestion) phases (Minekus et al., 2014). These in vitro conditions try to mimic physiological conditions in vivo and take into account the presence of digestive enzymes and their concentrations, pH, digestion time, and salt concentrations, among other factors (Minekus et al., 2014).

3 Collagen formulations tested

The tested samples were received in Bionos on 18/11/2023 at room temperature, and labelled.

The products were stored at room temperature (RT) at Bionos Biotech facilities.

4 Registration dates

- Study begins: 15/02/2022
- Study ends: 20/12/2024
- Experimental phase begins: 15/02/2023
- Experimental phase ends: 16/02/2024

5 Platform

Intestinal epithelial CaCo2 cells (HTB-37 - ATCC). And in vitro digestion model

6 Material and Methods

6.1 Analytical equipment

Inverted microscope, refrigerated centrifuge, thermoblock, micropipettes, serological pipettes, rack, laminar flow hood, vortex, cell culture incubator (37°C, 5 % CO_2, 90% relative humidity (RH), vortex, Bürker chamber, freezer -80ºC, freezer -20ºC, UPLC-MS/MS 6460, CORTECS C18 column (2.1 x 150mm x 1.6µm).

6.2 Reagents

CaCo2 cells (Quimigen) and specific culture media and supplements (Quimigen), Ultrapure Water (Sigma), Trypan Blue (Bio-Rad), Trypsin (Sigma-Aldrich), PBS (Gibco), MTT reagent [3-(4,5-Dimethylthiazol-2-yl)-2,5-Diphenyltetrazolium Bromide] (Invitrogen), FITC and 7AAD (Digma), ACN (Sigma), DMSO (Sigma), ethanol (Sigma), transwell 24-well plates (Fisher Sci), methanol (Sigma), AccQ TagTM CP U derivatization reagent kit (Waters). All the enzymes and chemicals for the in vitro digestion buffer preparation were purchased from Sigma.

6.3 Procedure

6.3.1 In vitro digestion of products and cell viability assessment

The products were digested following a simulated in vitro digestion method following the guidelines published by Minekus et al. (Minekus et al., 2014). Simulated digestion methods typically include the oral (amylase digestion), gastric (pepsin digestion) and small intestinal (intestinal enzymes digestion) phases (Minekus et al., 2014). These in vitro conditions try to mimic physiological conditions in vivo and take into account the presence of digestive enzymes and their concentrations, pH, digestion time, and salt concentrations, among other factors (Minekus et al., 2014). First, for the oral phase, products were dissolved the simulated salivary fluid (SSF) buffer (0.75 mM $CaCl_2$, 15.1 mM KCl, 3.7 mM KH_2PO_4, 13.6 mM $NaHCO_3$, 0.15 mM $MgCl_2(H_2O)_6$, 0.06 (mM $NH_4)_2CO_3$) and added human salivary α-amylase (EC 3.2.1.1) to achieve 75 U/mL. Mixture was incubated 2 minutes at 37ºC. Then, for the gastric phase, porcine pepsin (EC 3.4.23.1) was added to achieve 2000 U/mL in the final digestion mixture, followed the components of the simulated gastric fluid (SGF) buffer (0.075 mM $CaCl_2$, 6.9 mM KCl, 0.9 mM KH_2PO_4, 25 mM $NaHCO_3$, 47.2 mM NaCl, 0.1 mM $MgCl_2(H_2O)_6$, 0.5 (mM $NH_4)_2CO_3$, pH 3). Mixture was incubated 2 hours at 37ºC, checking and correcting the pH

during this incubation time. Finally, for the intestinal phase, pancreatin from porcine pancreas was added to achieve 800 U/mL in the final digestion mixture, followed the components of the simulated intestinal fluid (SIF) buffer (160 mM fresh bile, 0.3 mM CaCl2, 6.8 mM KCl, 0.8 mM KH2PO4, 85 mM NaHCO3, 38.4 mM NaCl, 0.33 mM MgCl2(H2O)6, pH 7). Mixture was incubated 2 hours at 37ºC, checking and correcting the pH during this incubation time. The digested products were then tested for their effect on CaCo2 cell viability by the MTT assay.

The viability of the CaCo2 cells upon treatment with the products was assessed by the MTT assay, following the ECVAM Guidelines as established in ECVAM Database Service on Alternative Methods to Animal Experimentation (MTT assay protocol #17). Cells were cultured overnight at a 10.000 cells/well density in a 96 well plate. 24 h later, the culture media was replaced with new culture medium containing the tested samples at 7 different concentrations (10, 5, 2, 1, 0.5, 0.25 and 0.125 mg/ml). After 24 h of incubation, the medium was removed, and the MTT solution was added to each well. Plates were incubated at 37ºC for 3 h. MTT reactive solution was removed, and 100% DMSO was added to each well to solubilize formazan crystals prior to absorbance measurements at 550 nm and 620 nm as reference on a scanning multi-well spectrophotometer. Data were statistically analysed.

6.3.2 Assessment of the CaCo2 cell monolayer

The 100% confluence of the CaCo2 cell monolayer in the transwells was determined by the permeability of the cell membrane-impermeable dyes FITC and 7AAD by fluorescence spectrometry. FITC and 7AAD are cell membrane-impermeable compounds that do not cross the intact cell membrane (Yao et al., 2017) (Moumaris et al., 2015) (Ito et al., 2010). CaCo2 cells were seeded in transwells (50.000 cells per transwell). After 2, 4, 6, 8, 10, 12, 14, 16 and 18 days, FITC (10 µg/ml) or 7AAD (10 µg/ml) dyes were added to the apical media (Figure 2). After 240 min of incubation, samples from the basolateral media were taken to detect the FITC or 7AAD dyes by fluorescence spectrometry (Figure 2). Samples from a condition without FITC or 7AAD were taken and used as controls. Absence of FITC/7AAD in the basolateral media indicates 100% culture confluence.

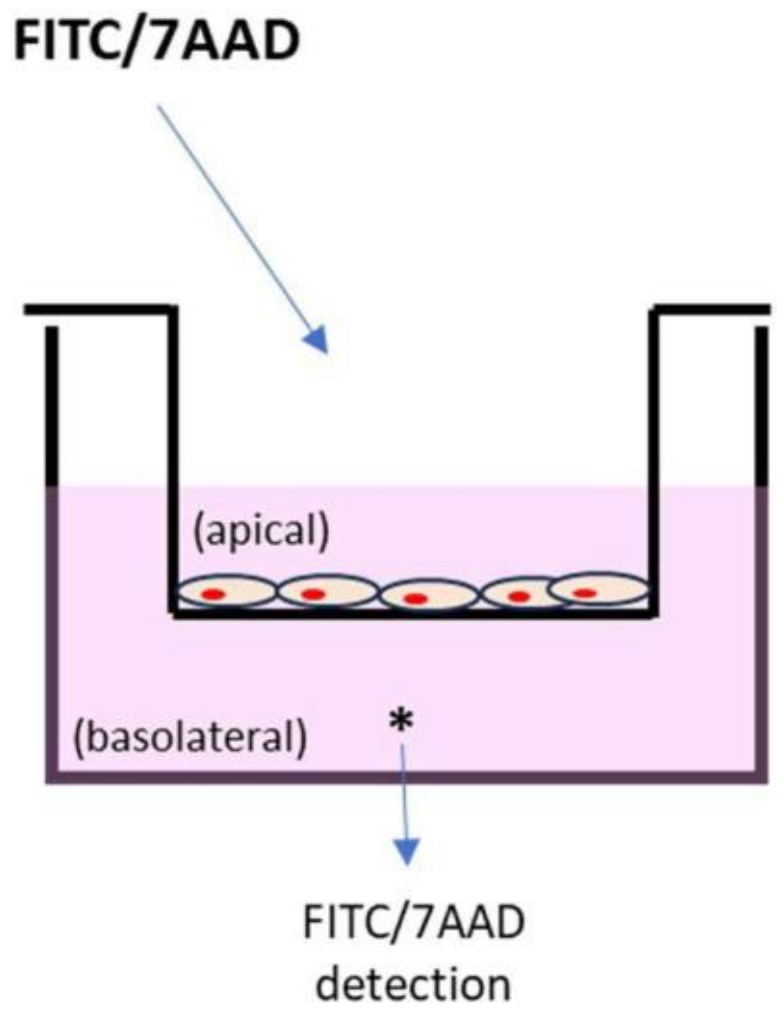

Figure 2. Scheme for the assessment of the CaCo2 cell monolayer.

6.3.3 In vitro bioavailability of collagen-derived amino-acids and peptides in the CaCo2 cell model

The digested products were added to the CaCo2 cell monolayer in the apical media at a concentration of 5 mg/ml (Figure 3). For placebo, an equal volume of the mix of in vitro digestion buffers without product was added to the cells. Then, the in vitro bioavailability of collagen-derived amino-acids and peptides (absorption and transport across the CaCo2 cell monolayer) was determined by quantifying the free amino-acids and peptides present in the basolateral media before adding the product (time 0, T0) and after 5, 10, 15, 30, 120 and 240 minutes (Figure 3).

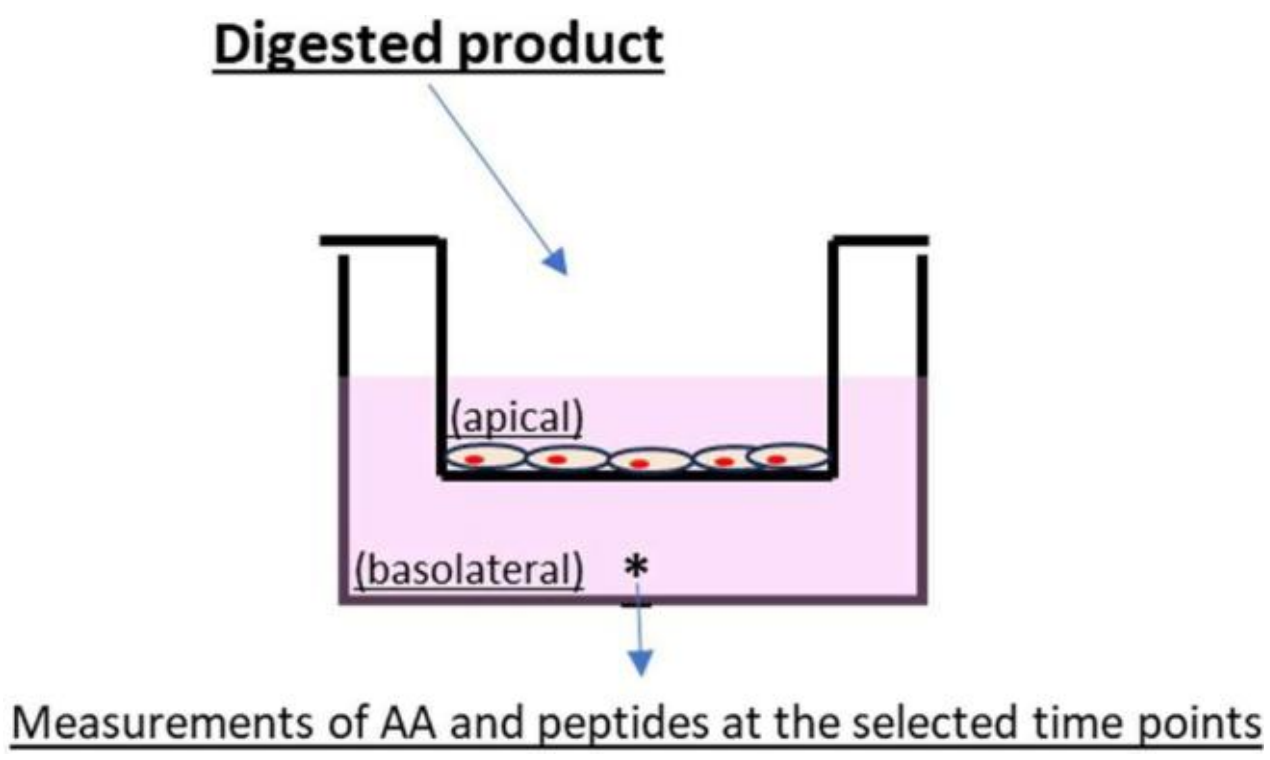

Figure 3. Scheme for the assessment of the in vitro bioavailability of collagen-derived amino-acids and peptides.

6.3.3.1 Quantification of collagen-derived amino-acids

The amino-acid quantification was performed using a methodology based on Waters' AccQ Tag method, widely used in the literature for the determination of amino-acids. Sample preparation consisted of protein precipitation by the addition of cold methanol and subsequent precolumn derivatization of the analytes, following the kit instructions. Once the sample was derivatized, it was injected into an Agilent 6460 UPLC-MS/MS. The chromatographic separation of the compounds was carried out using the CORTECS C18 column (2.1 x 150mm x 1.6µm). The mobile phases used were water and acetonitrile, both with 0.1% (V/V) formic acid. The analysis time was 9 minutes with a flow rate of 0.5 mL/min. The column temperature was 40ºC and the samples were kept in the autosampler at 4ºC during analysis. Alternatively, amino-acids were also quantified by ABSciex UHPLC-MS 6500+. For this method, chromatographic separation was performed using the SIELC Primesep 100 chromatographic column (100 x 3.2 mm, 3 µm). The used mobile phases were Water/Acetonitrile 70/30 v/v, 0.5% or 0.05% TFA v/v. The analysis time was 19 minutes with a flow rate of 0.5 mL/min. The column temperature was 30ºC.

6.3.3.2 Quantification of collagen-derived peptides (Ongoing methodology preliminary work)

The peptides quantified by UPLC-MS/MS in the tested samples.

Solvent and stock concentration of the reference peptides used for calibration (GenScript). From these stock solutions, individual standards and multicomponent standards of 10 mg/L were prepared to fine-tune the method and prepare the calibration curve. In order to quantify the samples, a multicomponent calibration curve was prepared on a matrix in the indicated ranges.

20 µL were transferred to an eppendorf containing 200 µL of water. Samples were vortex and centrifuged 10 min at 4°C and 11000 rpm. 50 µL of each sample were transferred to a chromatographic plate for analysis. The di-peptides and tri-peptides were quantified using the protocol based on the Waters method using the AccQTagTM CP U derivatization reagent kit by the Agilent UPLC-MS/MS 6460 (Heidenreich et al., 2021). The chromatographic separation was performed using the CORTECS C18 chromatographic column (2.1 x 150mm x 1.6µm). The used mobile phases were H2O/formic acid 0.1% and ACN/formic acid 0.1%. The analysis time was 9 minutes with a flow rate of 0.5 mL/min. The column temperature was 40ºC.

6.3.4 Statistical analysis

The MTT assay was set with 8 replicates per condition and the amino-acid quantification assay in the basolateral media of the CaCo2 cell transwell cultures assay was set with 2 replicates per condition. Data outliers were identified with the ROUT method (Q = 5 %) and excluded from the analysis if found. Data were statistically analyzed by one way ANOVA test and Holm Sidak´s or Dunnet's T3 post hoc multiple comparisons test. Statistical significance was declared at $p < 0.05$, 95 % of confidence. Bars in the charts represent the mean value for each condition and error bars indicate the standard error of the mean (SEM) for each group of values.

7 Results

7.1 Cell viability

Products were digested in vitro as described in Material and Methods and their effect on the viability of the CaCo2 cells was assessed by the MTT assay. As Figure 4 shows, when cells were treated with CP P at concentrations below 10 mg/ml, cell viability reached a plateu where the cell viability remained stable. This is most likely due to absorbance reading interference of some product component or an interference of the product with the cell growth, rather than a cytotoxic effect. Based on this, our conclusion is that CP P did not affect cell viability at concentrations below 10 mg/ml. On the other side, we observed that the rest of the products did not affect cell viability at concentrations below 10 mg/ml (Figure 4). Therefore, the selected working concentrations for all tested products was 5 mg/ml.

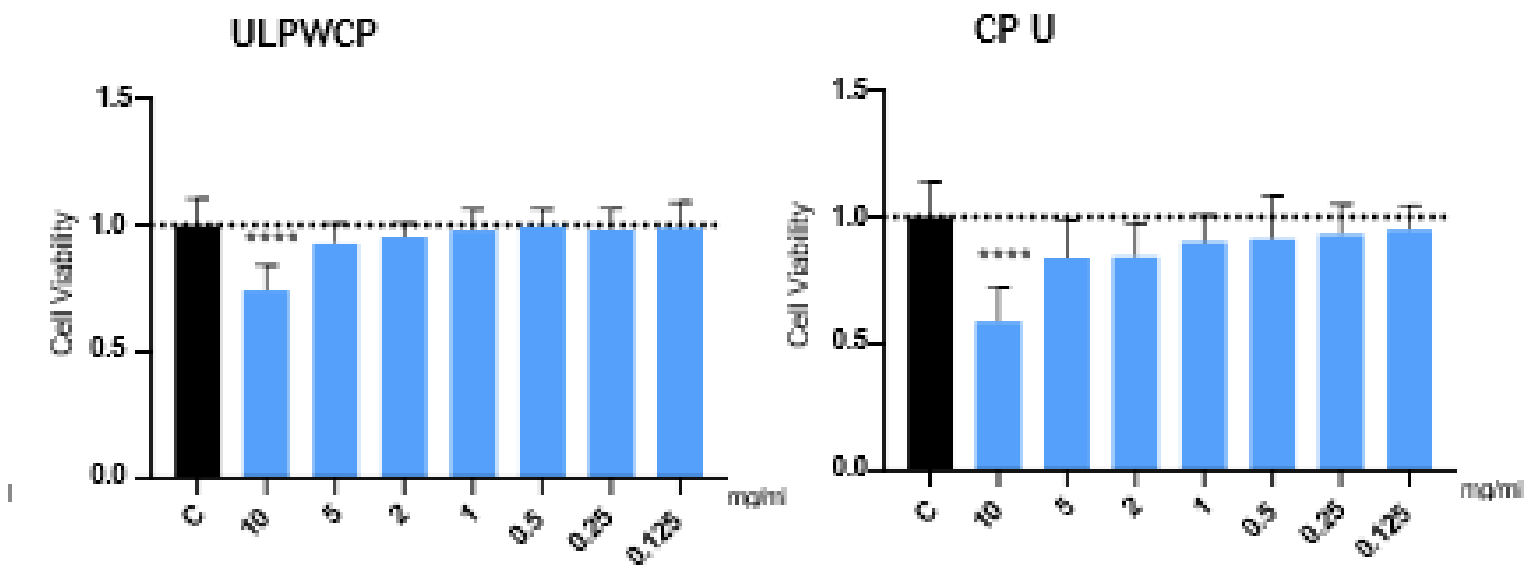

Figure 4. Graphical representation of the results showing the cell viability of CaCo2 cells after treatment with the digested products at the indicated concentrations for 24 h, compared to the non-treated control. Asterisks represent statistical significance as * for p-value < 0.05, ** for p-value < 0.01, **** for p-value < 0.0001.

7.2 Assessment of CaCo2 cell monolayer

The 100% confluence of the CaCo2 cell monolayer in transwells was determined by the permeability of cell membrane-impermeable dyes (FITC and 7AAD) by fluorescence spectrometry, as described in the Material and Methods section (Figure 5). The "only medium" condition refers to the intrinsic fluorescence signal of the culture media (background signal). The "cell-free transwell" condition refers to a well where the dyes were added to a transwell without CaCo2 cells, to assess whether the dyes can pass through the transwell filter. Results showed that the dyes pass freely through the transwell filter, given the fluorescence signal of the basolateral media detected in the "cell-free transwell" control condition (Figure 5). The FITC fluorescene signal in the basolateral media decreased gradually in a time-dependent manner, indicating that the diffusion of the FITC dye through the filter is reduced due to the CaCo2 cell proliferation and the establishment of a confluent monolayer (Figure 5). After 16 days of incubation in the transwells, the CaCo2 monolayer had a confluence of 100%, since no FITC or 7AAD were detected in the basolateral media (Figure 5).

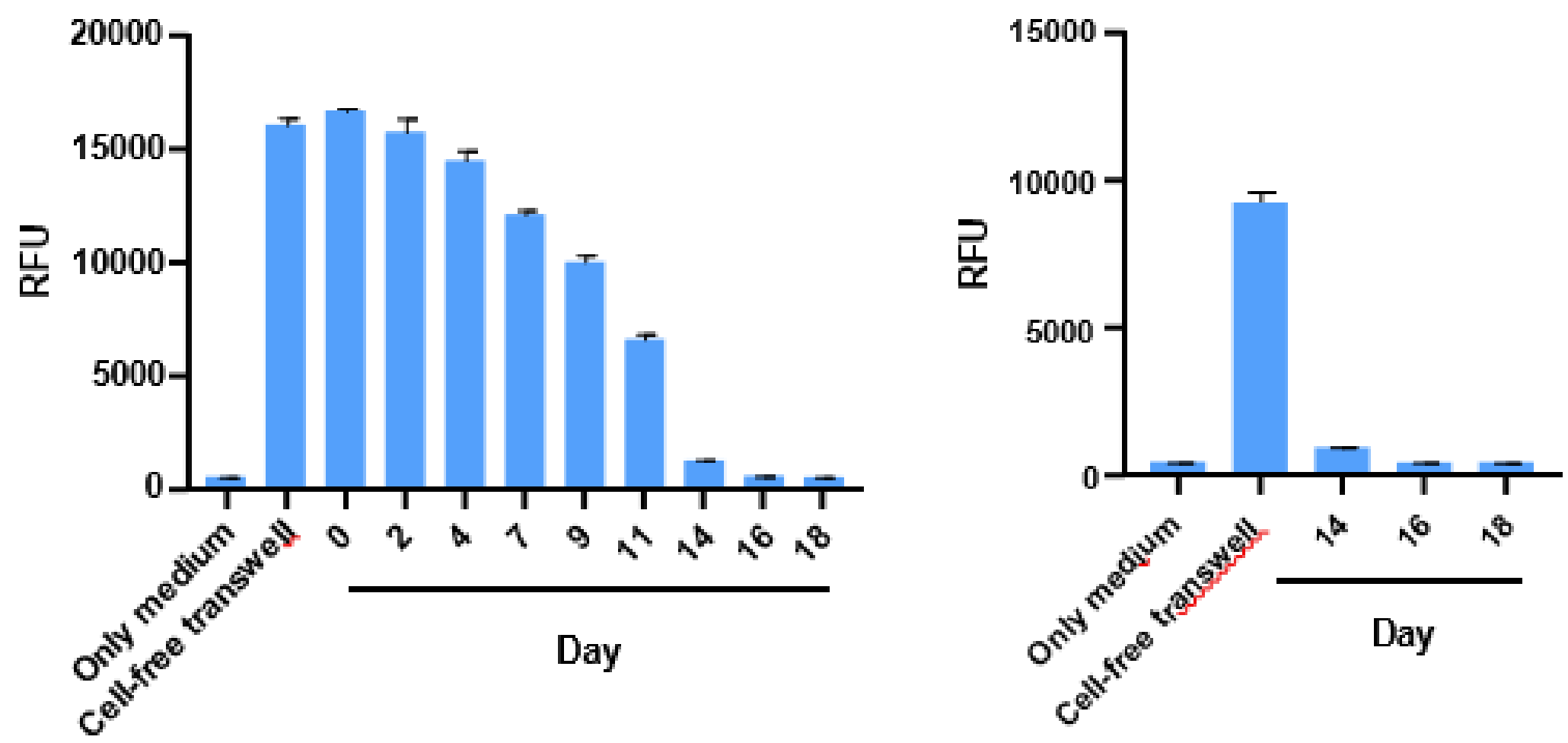

Figure 5. Graphical representation of the results showing the amount of dye (FITC or 7AAD) present in the basolateral media as an indicator of the monolayer confluency. "Only medium" and "Cell-free transwell" conditions refer to the amount of fluorescence detected in the culture media alone, and in the basolateral media of a transwell without cells, respectively.

In vitro bioavailability analysis

The placebo and digested products at a concentration of 5 mg/ml were added to the CaCo2 cell monolayer in the apical media, according to the cell viability results shown above (Figure 4). Then, the in vitro bioavailability of collagen-derived amino-acid was assessed by UPLC-MS/MS in the basolateral media before adding the product and after 5, 10, 15, 30, 120 and 240 minutes (Figure 3).

Tables 1 to 4 show the amino-acid levels for each assessed time point (in mg/L, mean ± SEM). In the first row of each table, it is indicated the value for the corresponding digested product, which corresponds to the amount of each amino-acid in the product that underwent the in vitro digestion process, was diluted in cell culture media and added to the apical medium of the CaCo2 cell experiment; in other words, it is the amount of each amino-acid that is present in the apical media, directly in contact with the CaCo2 cell monolayer. In Tables 1 to 4, the T0 corresponds to the Time 0, that is, the amino-acid levels in the basolateral culture media before adding the digested products. The rest of the rows in Tables 1 to 4 correspond to the amino-acid levels in the indicated time points. Data marked in green indicate a significant difference ($p\text{-value} < 0.05$) compared to the corresponding basal level (T0). Data marked in yellow indicate a statistical trend ($0.05 < p\text{-value} < 0.1$) compared to the corresponding basal level (T0).

In some time points, for some amino-acids, the value is higher than the digested product added to the cells. In some cases, this difference is minimal, what could be attributed to the biological variability of the experiment and the technical variability of the measurement (like Arginine in ULMWC). In other cases, the difference is higher (Alanine in ULMWC). This could indicate that the levels of those amino-acids are increased upon digestion and transport of peptides or larger proteins by CaCo2 cells.

After adding the digested products dissolved in cell culture media in the apical compartment, an increase in the amino-acid levels in the basolateral media was detected for some of the tested amino-acids at specific time points (Tables 1 to 4):

	Alanine	AlfaaminoButyricAcid	Aminoadipic acid	Arginine	Asparagine	Aspartic acid	Citrulline	Cystine
T0	22.8 ±0.3	1.1 ±0	0.5 ±0	72.3 ±0.9	26.7 ±0.6	7.5 ±0.2	2.7 ±0.3	25.3 ±1.9
5 min	24.9 ±0.3	1.1 ±0	0.5 ±0	77 ±3.8	29.4 ±1.4	7.8 ±0.3	3.4 ±0	27 ±1.5
10 min	28.3 ±3.4	1.2 ±0	0.7 ±0.1	81.8 ±8.9	31.7 ±3.8	9.3 ±1.3	3.4 ±0.3	27.8 ±0.4
15 min	26.7 ±0.8	1.2 ±0	0.6 ±0	83 ±0.8	31.2 ±0.2	9.2 ±0.3	3.1 ±0	27.6 ±0.1
30 min	25.4 ±0	1.2 ±0	0.5 ±0	76.8 ±1	29.8 ±0.5	8 ±0.2	2.6 ±0.1	26.9 ±0.7
120 min	28.8 ±0.5	1.2 ±0	0.7 ±0	83 ±3.5	33.2 ±1.5	8.7 ±0.1	3 ±0	27 ±0
240 min	29.7 ±0.4	1.2 ±0	0.6 ±0	86.1 ±4.3	35.3 ±1.9	7.2 ±0.6	2.7 ±0	26.5 ±1.3

	Glutamic acid	Glycine	Histidine	Hydroxylysine	Hydroxyproline	Isoleucine	Leucine	Lysine
T0	28.2 ±1.3	11.9 ±0.4	27.8 ±0	0.2 ±0	3.4 ±0	38.2 ±0.3	38.9 ±0.3	36.2 ±0.5
5 min	29.8 ±0.9	13.2 ±0.2	27 ±0.5	0.1 ±0	3.6 ±0	40.4 ±1.9	41.6 ±1.5	38.3 ±2.1
10 min	33.2 ±4.1	13.8 ±0.9	28.3 ±0.4	0.2 ±0	3.7 ±0.2	43.7 ±4.3	45.1 ±4.3	48.1 ±8.9
15 min	35.3 ±1.2	13.9 ±0	27.4 ±0.7	0.2 ±0	3.5 ±0	43.3 ±0.8	45.1 ±0.8	47.1 ±0.2
30 min	29.7 ±1.2	13.3 ±0	27.3 ±0.2	0.2 ±0	3.6 ±0	41 ±0.2	42.5 ±0.6	42.9 ±0
120 min	34 ±2.4	13.5 ±0	27.6 ±0	0.2 ±0	3.5 ±0.1	44.6 ±1.2	46.6 ±1.1	50.3 ±2.6
240 min	29.3 ±2	13.8 ±0.2	28.3 ±1	0.2 ±0	3.8 ±0.1	42.9 ±1.1	44.2 ±1	46.8 ±2.5

	Ornithine	Phenylalanine	Proline	Serine	Taurine	Threonine	Tryptophan	Valine	Tyrosine
T0	4.3 ±0.2	30.9 ±0.4	14.9 ±0.1	11.9 ±0.4	3.8 ±0	34.3 ±0.3	11.2 ±0.3	37.4 ±0	38.1 ±0.5
5 min	3.9 ±0.1	32.3 ±1.2	16.3 ±0.8	13.6 ±0.1	4 ±0	36.1 ±0.8	11.6 ±0.3	39.3 ±1.5	39.1 ±1.9
10 min	4.5 ±0.3	31.9 ±1.2	18.3 ±1.7	14.1 ±0.9	4.1 ±0.2	39 ±3.8	11.2 ±0.3	43.5 ±4.2	39.8 ±1.5
15 min	4.4 ±0.3	32.1 ±0	17.4 ±0.9	13 ±0	4.1 ±0.1	38.2 ±0.1	11.2 ±0	43.1 ±0.4	39.3 ±0.3
30 min	4.4 ±0.2	32.5 ±0.7	17.1 ±0	12.8 ±0.1	3.9 ±0.1	37 ±0	11.3 ±0.1	40.3 ±0.1	39 ±0.7
120 min	5.4 ±0	32.5 ±0.3	17.9 ±0	13.6 ±0.2	4 ±0	39.7 ±0.3	11.2 ±0	44.4 ±0.4	39.3 ±0.3
240 min	5 ±0.3	32 ±0.2	18.1 ±0.5	13.6 ±0.2	4.1 ±0	38.5 ±0.7	11.2 ±0	42.3 ±0.8	40 ±0.6

Table 1. Placebo values corresponding to the amino-acid levels in the basolateral media at the indicated time points. Data are represented in mg/L (mean ± SEM). Data marked in green indicate a significant difference (p-value < 0.05) compared to the corresponding basal level (T0min). Data marked in yellow indicate a statistical tendency (0.05 < p-value < 0.1) compared to the corresponding basal level (T0min).

	Alanine	AlfaaminoButyricAcid	Aminoadipic acid	Arginine	Asparagine	Asparticacid	Citrulline	Cystine
d Prima	28.1	1.8	0.7	79.9	28.5	10.5	2.8	34.0
T0	29.4 ±1.2	0.9 ±0.3	0.5 ±0.2	83.3 ±0.2	29.4 ±5.9	7.5 ±1	2.5 ±0.3	27.1 ±1
5 min	24.8 ±4.5	1 ±0.4	0.5 ±0	67.9 ±7.4	23.5 ±4.8	7.7 ±0.4	2.7 ±1.1	24.2 ±3
10 min	26.5 ±0.7	0.9 ±0.3	0.5 ±0	77.8 ±4.2	28.1 ±6.8	8.6 ±0	2.8 ±0.5	25.7 ±0.8
15 min	28.1 ±1.3	0.9 ±0.2	0.5 ±0.1	88.2 ±8.2	30.6 ±7.2	8.7 ±0.7	3.5 ±1.3	27.6 ±1.4
30 min	27.6 ±2	1 ±0.3	0.4 ±0	73.7 ±10.1	27.6 ±8.1	7.9 ±1.3	2.6 ±0.8	25.7 ±2.5
120 min	31.2 ±0.6	1 ±0.3	0.5 ±0	82.5 ±2.6	29.2 ±5.3	8.7 ±0.4	3.3 ±0.6	31.9 ±1.4
240 min	29.5 ±0	0.8 ±0	0.4 ±0	85.1 ±1.1	22.9 ±0.4	8.7 ±0.2	2.2 ±0	31.2 ±0.6

	Glutamic acid	Glycine	Histidine	Hydroxylysine	Hydroxyproline	Isoleucine	Leucine	Lysine
d Prima	36.8	17.8	27.2	0.4	3.7	45.6	48.4	50.9
T0	32.2 ±0.5	11.6 ±2.2	25 ±1.5	0.1 ±0	3.4 ±0.2	42.2 ±0.8	44.6 ±0.6	44.5 ±4.7
5 min	26.1 ±3.1	13.1 ±1.1	22.2 ±2.6	0.1 ±0	3 ±0.5	42 ±0.6	42.9 ±0.1	35.8 ±8.2
10 min	30.3 ±0.1	13.2 ±0.8	24.2 ±1.5	0.1 ±0	4 ±0.1	41.3 ±0.5	43.5 ±0.2	39.7 ±6.1
15 min	31.6 ±1.7	14 ±3.1	25.9 ±3.2	0.2 ±0.1	4.1 ±0	44.1 ±0	45.6 ±0.3	41.8 ±4.8
30 min	28.2 ±6.1	13.5 ±0.9	23.6 ±5.9	0.2 ±0	3.6 ±0	45 ±0.2	46.2 ±0.4	41.7 ±8.4
120 min	34.4 ±1	14.4 ±1.5	28.3 ±2	0.2 ±0	4.3 ±0.1	48.6 ±0.2	47.5 ±3.1	48.1 ±5.2
240 min	33.2 ±1.2	13.2 ±0.3	20.5 ±2.6	0.1 ±0	3.9 ±0.2	44 ±2.4	42.6 ±0.1	43.3 ±3.1

	Ornithine	Phenylalanine	Proline	Serine	Taurine	Threonine	Tryptophan	Valine	Tyrosine
d Prima	11.6	34.5	19.4	12.2	2.8	41.1	10.8	45.4	40.5
T0	6.1 ±0.6	31.8 ±0	17.8 ±1	13.5 ±0.6	3 ±0.7	38.4 ±0	10 ±0.6	41.1 ±0.6	39.6 ±0.8
5 min	5.7 ±1.9	28.6 ±3.3	15.1 ±2.1	11.1 ±1.4	2.7 ±0.7	31.7 ±3.8	9.1 ±1.3	40.7 ±0.1	39.3 ±0.4
10 min	4.1 ±0.7	30.4 ±1.3	16.6 ±0.7	13.3 ±0.7	3.2 ±0.7	37.7 ±0.4	9.7 ±0.9	42 ±0.6	40 ±0.4
15 min	4.4 ±1.1	32.3 ±1.5	17.2 ±1.3	15.8 ±2.4	3 ±0.8	38.1 ±1.5	10.2 ±1.1	43.7 ±0.7	41.5 ±0.9
30 min	4.9 ±1	29.5 ±2.6	17.2 ±1.5	12.6 ±1.4	3.1 ±0.7	37.3 ±2.5	9.5 ±1.1	44.8 ±0.4	39 ±0
120 min	5.6 ±0.2	35.8 ±1.5	19 ±1	15.7 ±0.1	3.5 ±1.3	44 ±0.6	11.8 ±1.5	47 ±0.3	43.8 ±1.1
240 min	4.6 ±0.7	30.7 ±1.9	16.9 ±0	14.6 ±0	2.6 ±0.1	39.6 ±1.2	8.9 ±1	45.8 ±0.2	43.5 ±0

Table 2. Values corresponding to the amino-acid levels in the apical compartment of the CaCo2 cell transwell (Digested CP P) and in the basolateral media at the indicated time points. Data are represented in mg/L (mean ± SEM). Data marked in green indicate a significant difference (p-value < 0.05) compared to the corresponding basal level (T0min). Data marked in yellow indicate a statistical tendency (0.05 < p-value < 0.1) compared to the corresponding basal level (T0min).

	Alanine	AlfaaminoButyricAcid	Aminoadipic acid	Arginine	Asparagine	Asparticacid	Citrulline	Cystine
d Ultra	28.3	1.8	0.7	90.8	32.3	10.6	3.6	35.4
T0	22 ±0.8	1.1 ±0	n.d.	66.2 ±5.8	n.d.	7.3 ±0.3	2.7 ±0.3	n.d.
5 min	24.8 ±2	1.1 ±0	0.5 ±0.1	72.4 ±8	30.6 ±4.7	8.2 ±1	3.3 ±0.1	24.7 ±2.3
10 min	23.9 ±4.1	1 ±0.1	0.5 ±0.1	74.4 ±6.8	31.3 ±3.4	8.4 ±1.5	3.3 ±0.3	22.7 ±2.9
15 min	22.5	1.1	0.4	63.9	29.6	8.5	3.3	21.7
30 min	24.8 ±2	1.1 ±0	0.6 ±0	70.2 ±8.7	30.4 ±1.6	8.2 ±1.1	2.8 ±0.4	23.6 ±3.2
120 min	25.3 ±1.2	1.2 ±0	0.5 ±0	67 ±4.1	30.9 ±1.6	7.4 ±0	2.7 ±0.3	22 ±1.5
240 min	27.3 ±2.2	1.1 ±0	0.4 ±0.1	71.1 ±7.9	30.7 ±2.6	6.5 ±0.3	2.7 ±0.2	21.2 ±2.3

	Glutamic acid	Glycine	Histidine	Hydroxylysine	Hydroxyproline	Isoleucine	Leucine	Lysine
d Ultra	38.5	16.6	28.4	0.4	3.7	47.3	49.3	54.3
T0	27 ±0.5	11.5 ±1	n.d.	n.d.	n.d.	35.1 ±2.8	36.9 ±3	36.2 ±0.6
5 min	28.8 ±3	12.7 ±0.7	23.7 ±1.1	0.2 ±0	3.4 ±0	38.4 ±2.7	39.8 ±3.1	41.6 ±5.2
10 min	29.5 ±5.7	12.4 ±1.7	24.4 ±0.4	0.1 ±0	3.4 ±0	38.4 ±6.3	39.8 ±6.4	40.4 ±10.8
15 min	27.6	11.4	20.3	0.2	2.8	34.9	36.1	36.5
30 min	30.9 ±4	11.9 ±1	24.5 ±0.2	0.2 ±0	2.9 ±0.3	38.1 ±4.5	39.5 ±4.7	42.7 ±5.1
120 min	27.5 ±1	12 ±0.7	23.9 ±1.1	0.2 ±0	2.8 ±0.1	37.4 ±1	38.6 ±0.6	42.1 ±2.1
240 min	26.5 ±1.7	12.8 ±1.1	24.2 ±2.1	0.2 ±0	3.3 ±0.2	38 ±3	39.1 ±3.1	44.6 ±2.9

	Ornithine	Phenylalanine	Proline	Serine	Taurine	Threonine	Tryptophan	Valine	Tyrosine
d Ultra	10.9	33.8	17.9	13.6	2.9	41.3	10.9	45.7	50.1
T0	4.3 ±0	26.9 ±2.7	14.7 ±1.1	11.2 ±0.7	3.4 ±0.4	30.4 ±1.3	9.9 ±1.1	35.2 ±2.5	33.3 ±3.3
5 min	5.2 ±0.7	29.7 ±1.2	15.5 ±0.9	12.1 ±0.6	4.2 ±0.4	34.3 ±3	10.5 ±0.2	37.8 ±3.6	36.3 ±1.3
10 min	4.8 ±1.5	29.8 ±1.8	15 ±1.8	12.6 ±1.7	3.8 ±0.1	33.9 ±5.4	10.6 ±0.2	37.6 ±5.6	34.9 ±2
15 min	5.0	26.1	14.2	11.9	3.7	32.1	8.9	34.3	30.8
30 min	5.1 ±0.4	27.3 ±2.6	15.1 ±1.3	12.1 ±1.6	3.6 ±0.1	34.3 ±3.9	9.3 ±0.6	37.1 ±4.3	32.9 ±2.7
120 min	6 ±0.2	27.3 ±0.5	15.5 ±0.7	12.1 ±0.1	3.3 ±0	33.5 ±0.8	9.4 ±0.1	36.9 ±1.6	33.4 ±1.2
240 min	6.3 ±0.5	28.5 ±1.8	17.2 ±1	12.5 ±1.1	3.4 ±0.2	34.8 ±1.8	9.6 ±0.5	38 ±2.3	34.4 ±2.1

Table 3. Values corresponding to the amino-acid levels in the apical compartment of the CaCo2 cell transwell (Digested CP U) and in the basolateral media at the indicated time points. Data are represented in mg/L (mean ± SEM). Data marked in green indicate a significant difference (p-value < 0.05) compared to the corresponding basal level (T0min). Data marked in yellow indicate a statistical tendency (0.05 < p-value < 0.1) compared to the corresponding basal level (T0min).

	Alanine	AlfaaminoButyricAcid	Aminoadipic acid	Arginine	Asparagine	Aspartic acid	Citrulline	Cystine
d Supra	26.7	1.6	0.7	78.0	24.6	11.1	3.8	25.8
T0	24.8 ±0.3	1.2 ±0	0.6 ±0	71.6 ±0.1	34.6 ±0.7	7.2 ±0	2.7 ±0	22.9 ±0.8
5 min	23.6 ±1.1	1 ±0	0.5 ±0	69.4 ±1.3	32.3 ±2.3	7.8 ±0.4	2.4 ±0	22.5 ±2.3
10 min	26.6 ±0.5	1.1 ±0	0.6 ±0	71.8 ±1.1	35.6 ±2.4	9.3 ±0	2.7 ±0	23.7 ±0.3
15 min	26.2 ±1.9	1.2 ±0	0.7 ±0	71.6 ±1	33.8 ±0.6	7.4 ±0.8	2.6 ±0	22.1 ±0.1
30 min	25.8 ±1.1	1.2 ±0	0.5 ±0	72.5 ±7.5	34.1 ±1.3	9 ±0.6	2.8 ±0.3	22.8 ±1.2
120 min	26.3 ±0.9	1.2 ±0	0.6 ±0	76.7 ±0.1	33.8 ±0.1	8.9 ±0.6	2.9 ±0.3	25.5 ±0.8
240 min	32.9 ±2.3	1.3 ±0	0.7 ±0	80.2 ±3.6	38.3 ±0.8	10 ±2.7	3.2 ±0	25.5 ±0.7

	Glutamic acid	Glycine	Histidine	Hydroxylysine	Hydroxyproline	Isoleucine	Leucine	Lysine
d Supra	29.4	18.2	29.3	0.3	4.1	39.0	42.0	38.3
T0	27.6 ±0.1	11.7 ±0.1	21.7 ±0.7	0.2 ±0	2.9 ±0	37.6 ±0.2	38.9 ±0.3	43.1 ±0.8
5 min	29 ±2.2	11.4 ±0.6	22.7 ±0.1	0.2 ±0	2.9 ±0.1	36.6 ±2.3	38.3 ±2.2	41.8 ±2.7
10 min	32.8 ±1.5	12.7 ±0.7	23.5 ±1.1	0.3 ±0	3.4 ±0	41.2 ±0.6	42.2 ±1	48.1 ±1.6
15 min	28.3 ±2.5	11.9 ±0.1	23.8 ±0	0.2 ±0	3.2 ±0	38 ±0	39.5 ±0	43.6 ±1.3
30 min	32.2 ±2	12.7 ±0.6	23.9 ±0	0.3 ±0	3.1 ±0.2	37.2 ±0.4	41.5 ±2.6	45.4 ±1.8
120 min	32.8 ±1.9	13 ±0.7	23.6 ±0.7	0.3 ±0	3.2 ±0	41 ±1.9	42.4 ±1.5	46.7 ±2.8
240 min	28.9 ±0.9	14.4 ±0.1	26.8 ±0.1	0.3 ±0	3.5 ±0.1	42.9 ±0.3	44.6 ±0.5	49.4 ±1.6

	Ornithine	Phenylalanine	Proline	Serine	Taurine	Threonine	Tryptophan	Valine	Tyrosine
d Supra	8.3	34.4	17.0	15.7	3.0	33.4	10.6	36.3	37.1
T0	5.9 ±0	26.9 ±0.2	15.2 ±0.1	11.4 ±0.1	3.2 ±0	33.6 ±0.5	8.8 ±0	37.5 ±0.4	33.2 ±0.6
5 min	5.2 ±0	26.6 ±1.1	14.3 ±1	10.9 ±0.4	3.2 ±0.1	32.7 ±1.5	8.9 ±0.4	36.1 ±1.9	31.7 ±1.4
10 min	6.3 ±0.4	29.5 ±0.8	17.1 ±0.7	12.3 ±0.8	3.4 ±0.1	36.2 ±0.5	9.7 ±0.2	40.2 ±0.9	36 ±0.8
15 min	6.7 ±1.1	28.1 ±0.3	16.4 ±1	11.1 ±0.2	3.2 ±0.1	34 ±0.3	9.4 ±0.1	37.4 ±0.3	33.9 ±0.8
30 min	5.7 ±0	28.4 ±1.5	15.6 ±0.6	12.2 ±0.8	3.3 ±0	34.3 ±2.1	9.3 ±0.3	39.2 ±1.7	34.1 ±1.9
120 min	5.7 ±0.4	29.5 ±0.7	16 ±0.6	12.7 ±0.2	3.6 ±0	35.8 ±0.3	9.7 ±0	40 ±2	36 ±1.5
240 min	6.9 ±0.3	32.7 ±0	18.5 ±0.1	14.2 ±0	3.4 ±0.1	39.1 ±0.2	10.6 ±0.1	42.8 ±0.1	39.3 ±0.7

Table 4. Values corresponding to the amino-acid levels in the apical compartment of the CaCo2 cell transwell (Digested ULMWC) and in the basolateral media at the indicated time points. Data are represented in mg/L (mean ± SEM). Data marked in green indicate a significant difference (p-value < 0.05) compared to the corresponding basal level (T0min). Data marked in yellow indicate a statistical tendency (0.05 < p-value < 0.1) compared to the corresponding basal level (T0min).

To address the observed low amino acid concentrations in the initial quantification, a dilution test was conducted using the ULMWC product in both its original and digested form. The experiment involved supplementing the CaCo2 culture media with ULMWC at increasing concentrations of 1 mg/mL, 5 mg/mL, and 10 mg/mL, followed by quantification of total, essential, non-essential, and collagen-specific amino acids.

The results demonstrate that while the concentration of amino acids increases with higher amounts of supplemented product, the increase is not proportional to the added concentration. Specifically:

- For collagen amino acids (glycine, hydroxyproline, proline), the observed trend indicates that increasing the ULMWC concentration from 1 mg/mL to 10 mg/mL results in incremental increases rather than linear proportionality. For example:
    - Glycine increases from 15.19 mg/L (1 mg/mL digested) to 24.30 mg/L (10 mg/mL digested), an increase of approximately 1.6-fold, despite the product concentration being increased 10-fold.
    - Hydroxyproline rises from 5.59 mg/L to 8.94 mg/L, a 1.6-fold increase.
    - Proline increases from 16.23 mg/L to 28.28 mg/L, approximately 1.7-fold.

Once we validated our analytical method for amino-acid quantification in the tested samples, we represented the curves showing the levels of the different amino-acid types (in mg/L and normalized to T0) in the basolateral media for each product at the different tested time points according to the UPLC-MS/MS method (Tables 1 to 4). In addition, the area under the curve was calculated for each product curve and represented in bar graphs, considering the total duration of the experiment (240 min) and different time intervals (from T0 to 5 min, 10 min, 15 min, 30 min and 120 min).

Try-outs about the concentration in the in vitro digestion reaction (up to 2x) and to perform a MTT assay at 6 hours to evaluate the short-term cytotoxicity.

- Since enzyme inactivation did not work to reduce the cytotoxic effect of the digested products in the CaCo2 cell model, we developed and alternative strategy to load more digested product on the CaCo2 cells without altering the cell viability.
- For this, first we repeated the in vitro digestion of the products by loading a higher amount as input. This yielded digested product samples at a higher concentration. This is important since, in the MTT viability assay, we can load more digested product by using the same volume.
- The cytotoxicity of these new digested products was evaluated by the MTT assay at 6 h (Figure 17).
    - The standard MTT assay is read after 24 h; however, since the high concentrations of the digested products were cytotoxic at this time-point, and the duration of the experiment was 4 h, we performed the MTT assay at 6 h.
    - The extent of the cytotoxic effect of a compound depends, among other factors, on the exposure time to the cells. A compound that is toxic after 24 h of exposure, may not be at 6 h.
    - Although the MTT assay is performed after 24 h of incubation to properly evaluate the cytotoxic effect of a product, the evaluation of the bioavailability in this project takes place within the first 4 h. Therefore, we evaluated whether during these 4 hours cytotoxic effects are observed, extending the time-point up to 6 h, to make sure that the concentrations we are using do not have a cytotoxic effect during the duration of the experiment.
    - Nevertheless, it is important to note that these data only provide information of the cytotoxic effect of the products during the experiment, but it is not suitable to evaluate the cytotoxicity by the standard MTT protocol.
    - In the original MTT assay performed at 24 hours, the digested products were cytotoxic to CaCo2 cells at concentrations higher than 5 mg/ml.

Altogether, our results showed that supplementation with the digested ULMWC increases the in vitro bioavailability of collagen amino-acids (Glycine, Proline, Hydroxylysine and Hydroxyproline) after 240 minutes. A higher input (> 5 mg/ml) of digested product in the in vitro bioavailability model does not show a cytotoxic effect for the CaCo2 cells during the first 6 hours of incubation.

In vitro bioavailability of collagen-derived peptides (ongoing methodology preliminary workflow)

Collagen-derived peptides in vitro were assessed by quantifying the selected di- and tri-peptides (Pro-Hyp, Hyp-Gly, Ala-Hyp, Gly-Pro, Gly-Pro-Hyp, Pro-Hyp-Gly, Ala-Hyp-Gly and Ser-Hyp-Gly).

First, the detection of the purified standards of each peptide was optimized in the UPLC-MS/MS system. For each peptide, below is shown a representative chromatogram of the analysis, indicating the acquisition time for each of them. In addition, for each peptide, a graph showing the corresponding mass-to-charge (m/z) ratio is also included, indicating the transition points in the top of the graph. First, the detection of the purified standards of each peptide was optimized in the UPLC-MS/MS system. For each peptide, below is shown a representative chromatogram of the analysis, indicating the acquisition time for each of them. This part is an ongoing project

Hyp-Gly

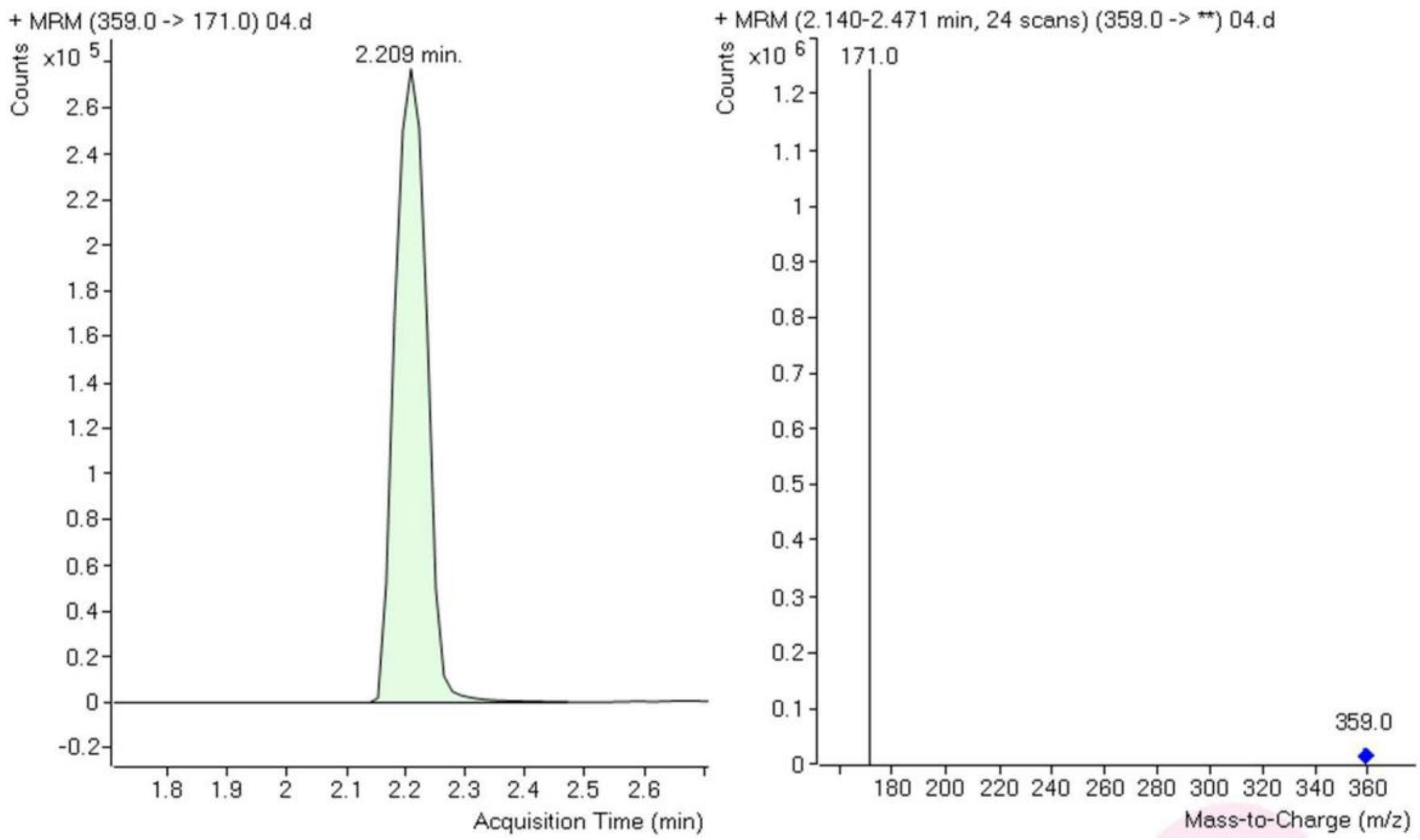

Ala-Hyp

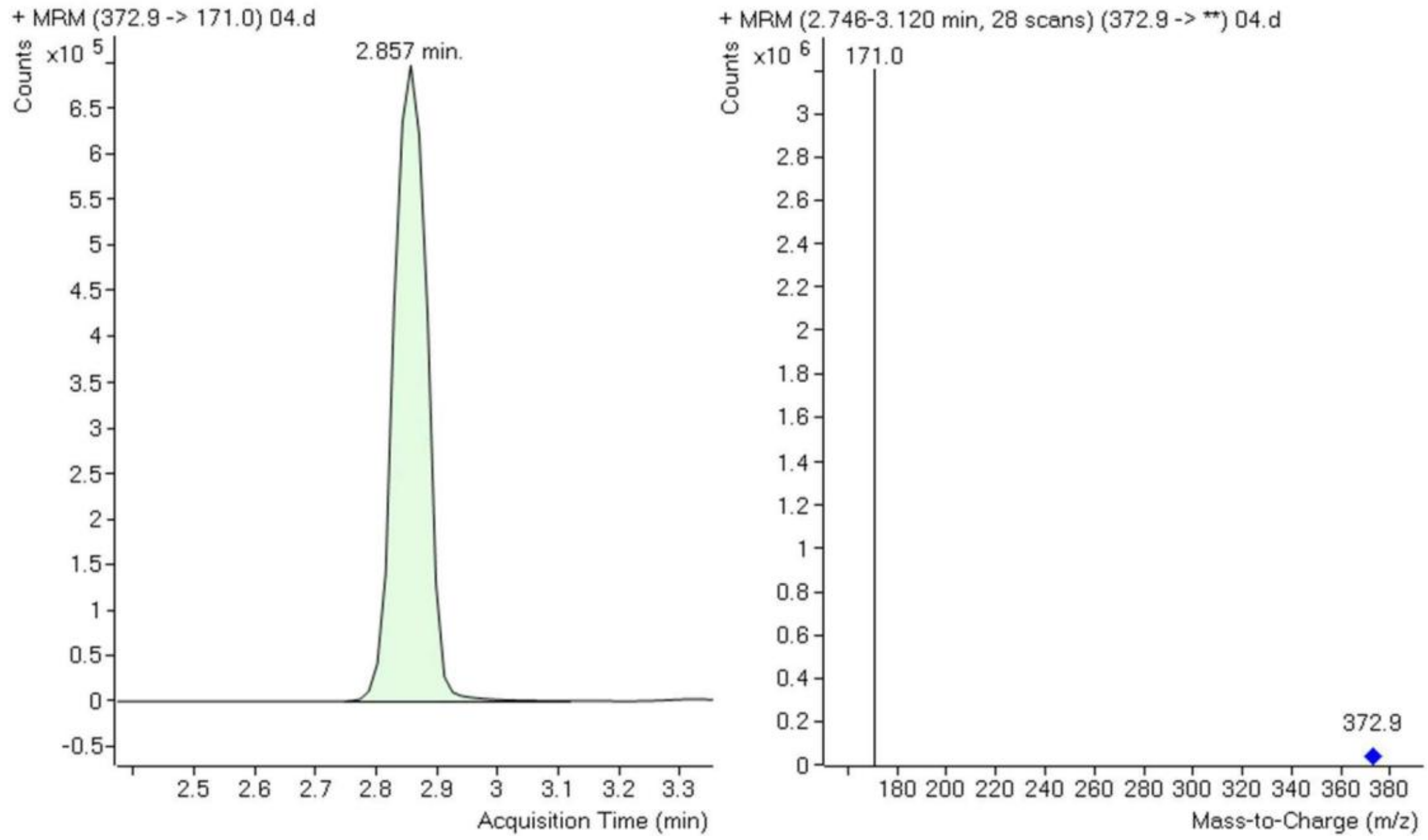

Gly-Pro

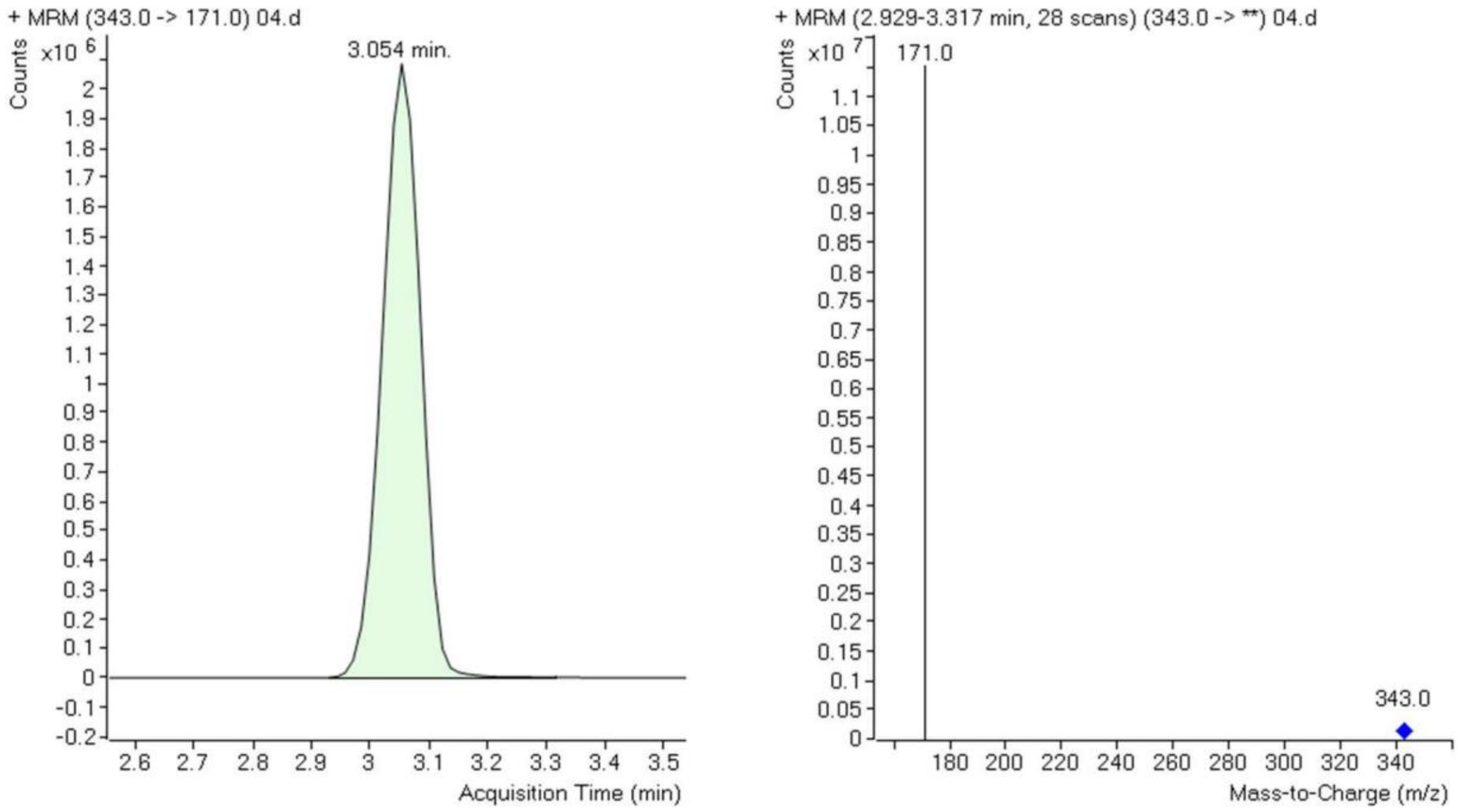

Gly-Pro-Hyp

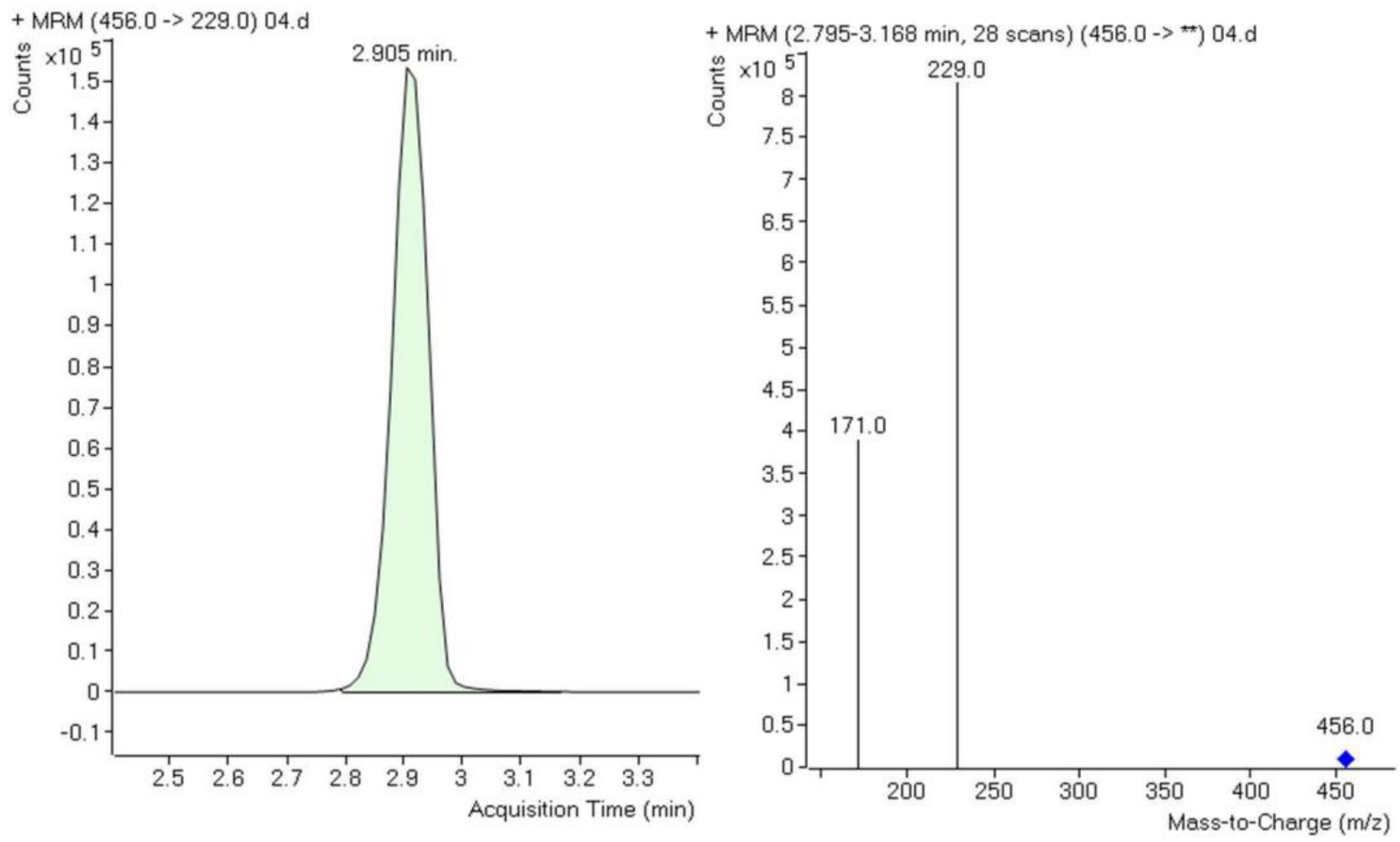

Ala-Hyp-Gly

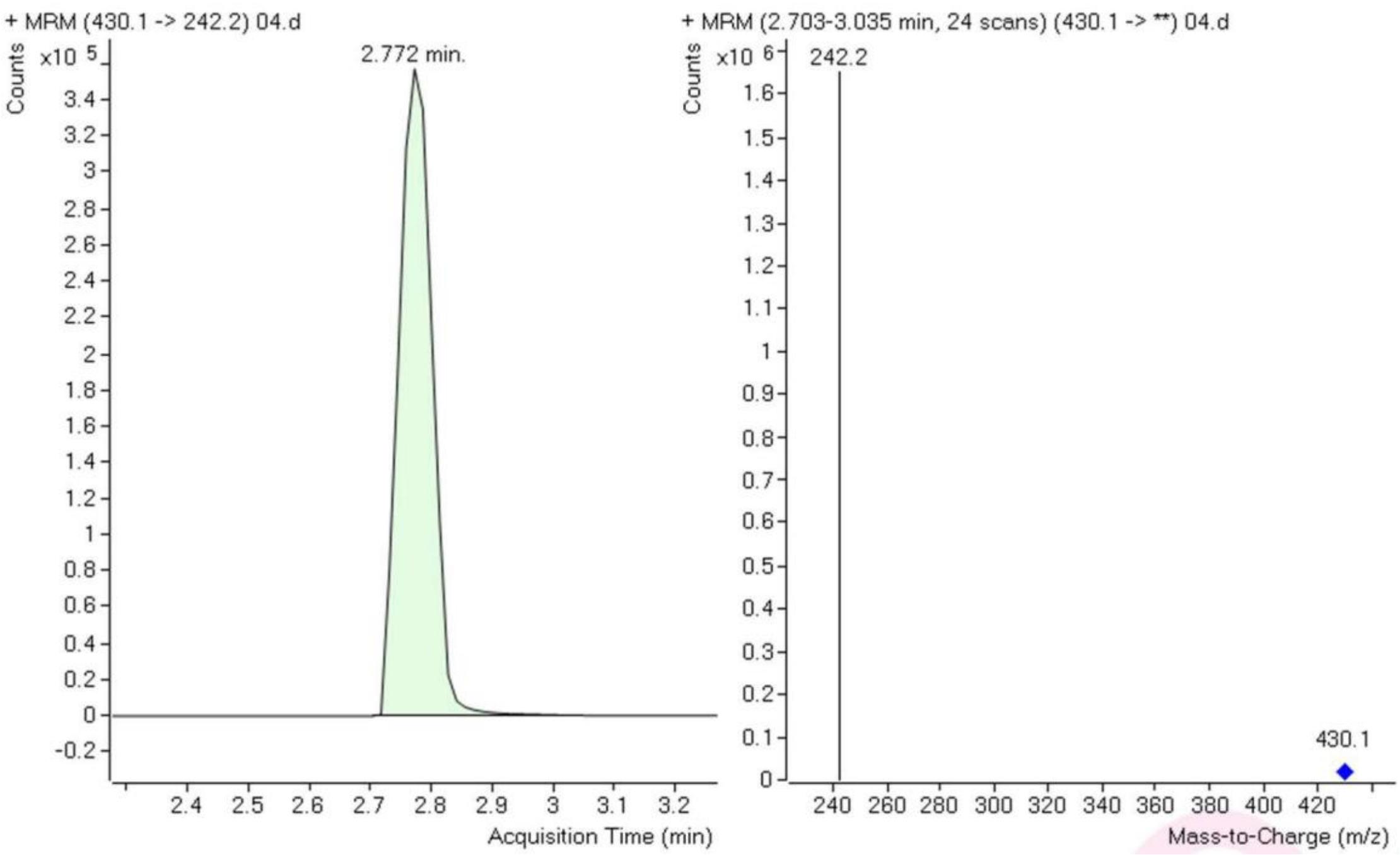

Ser-Hyp-Gly

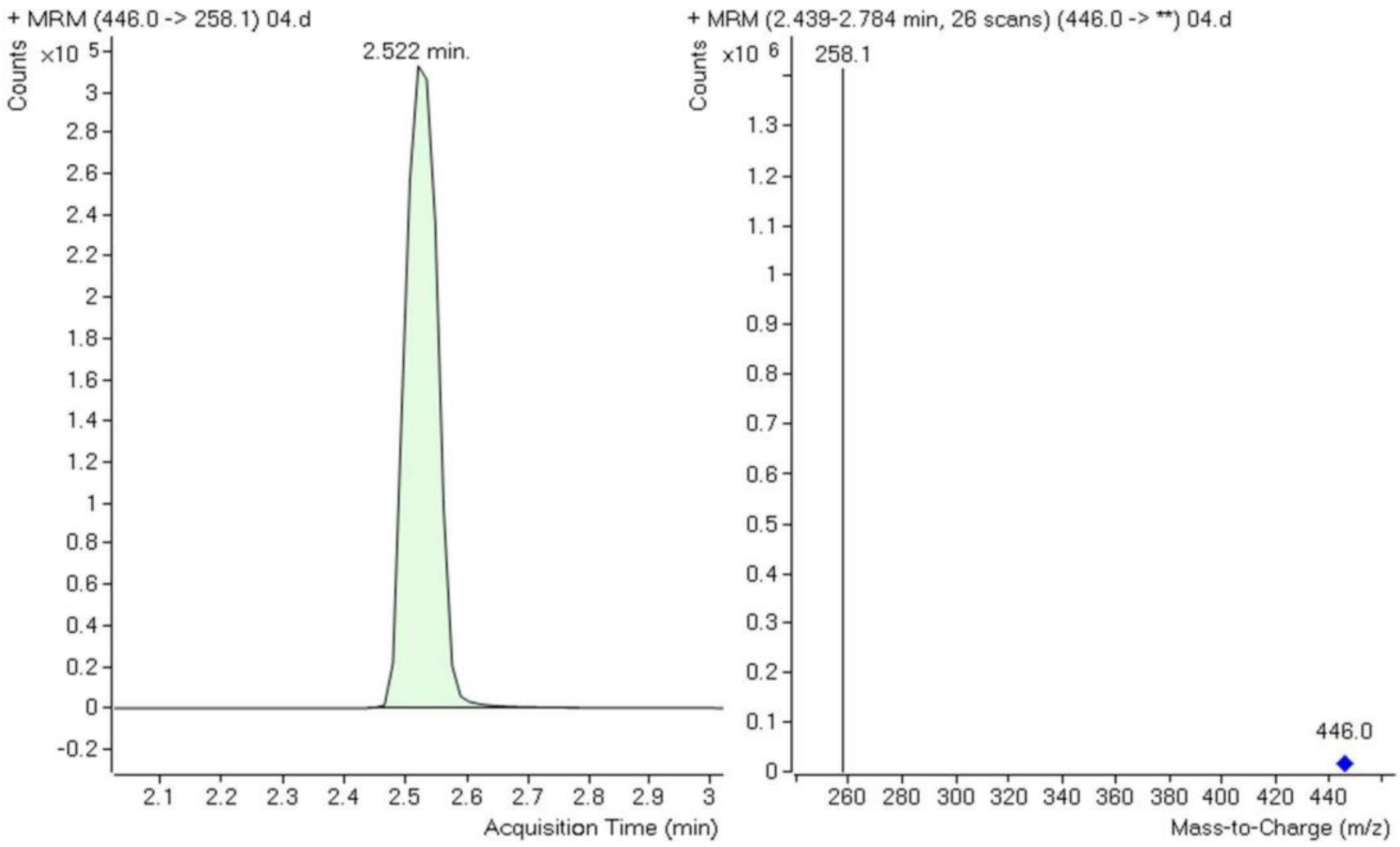

Pro-Hyp-Gly

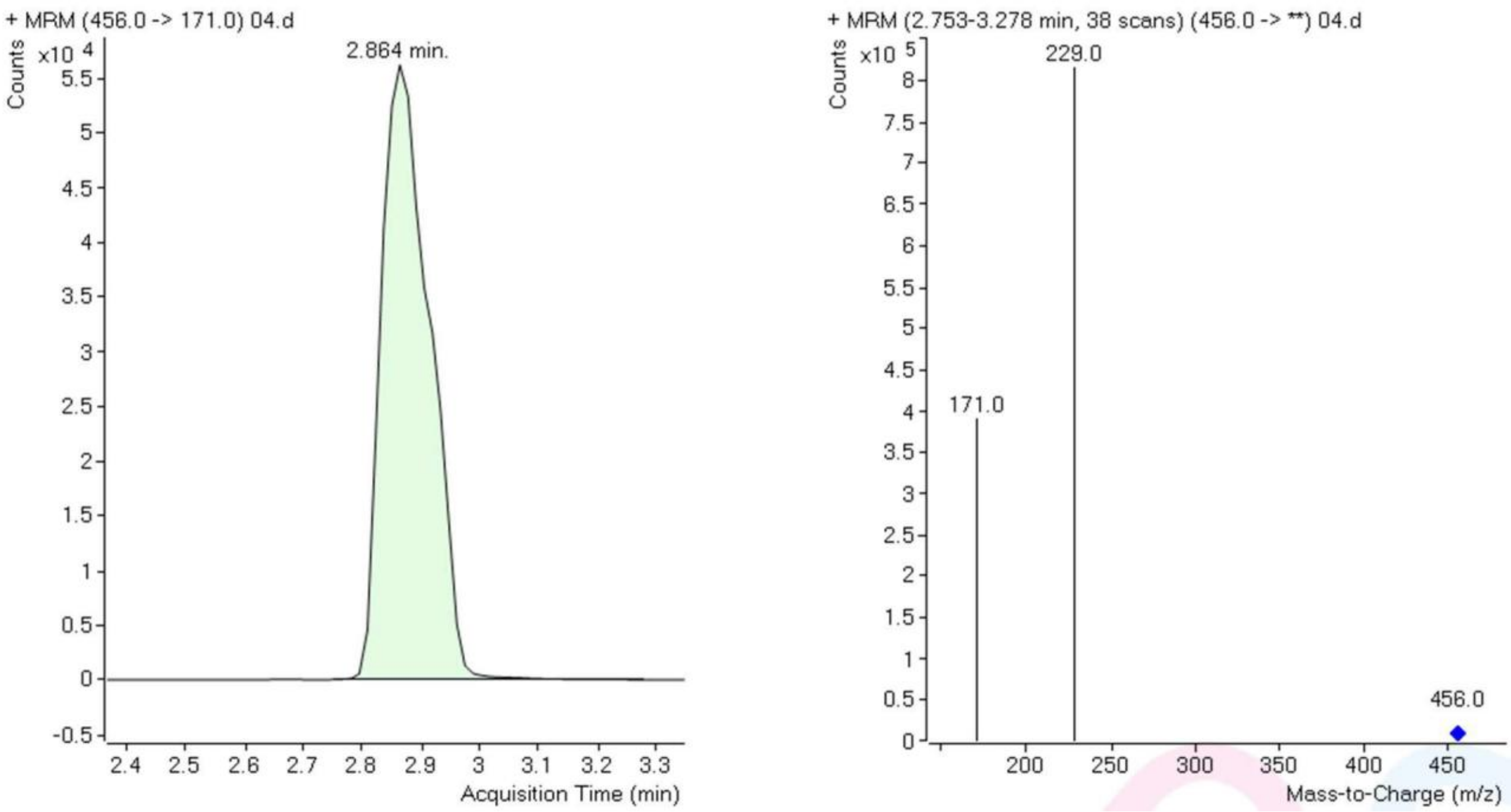

A calibration curve was then obtained for each peptide and the correlation index (R) is indicated.

The peptide standards were successfully detected by our protocol, demonstrating that the developed analytical method can detect these peptide samples.

Hyp-Gly

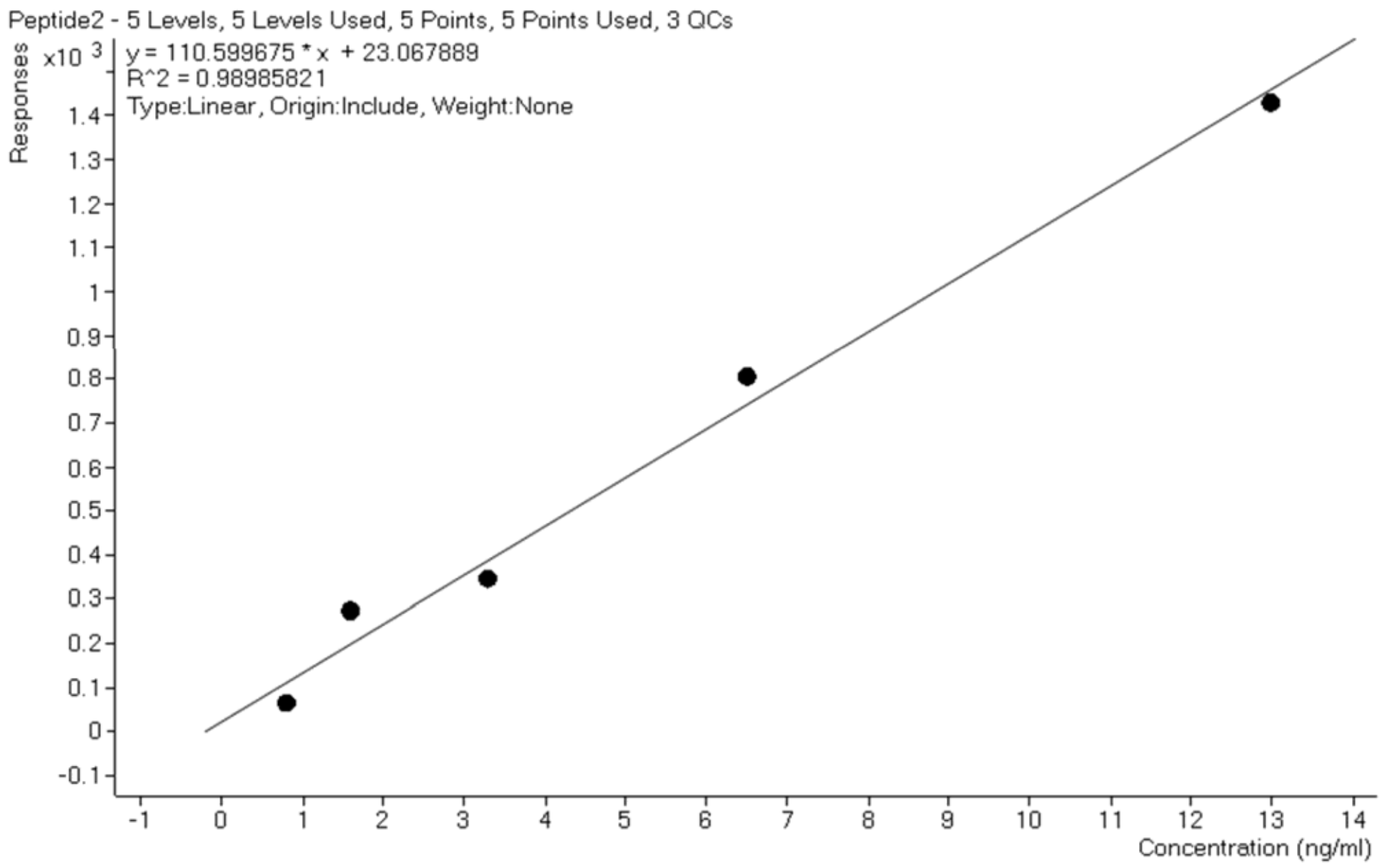

Ala-Hyp

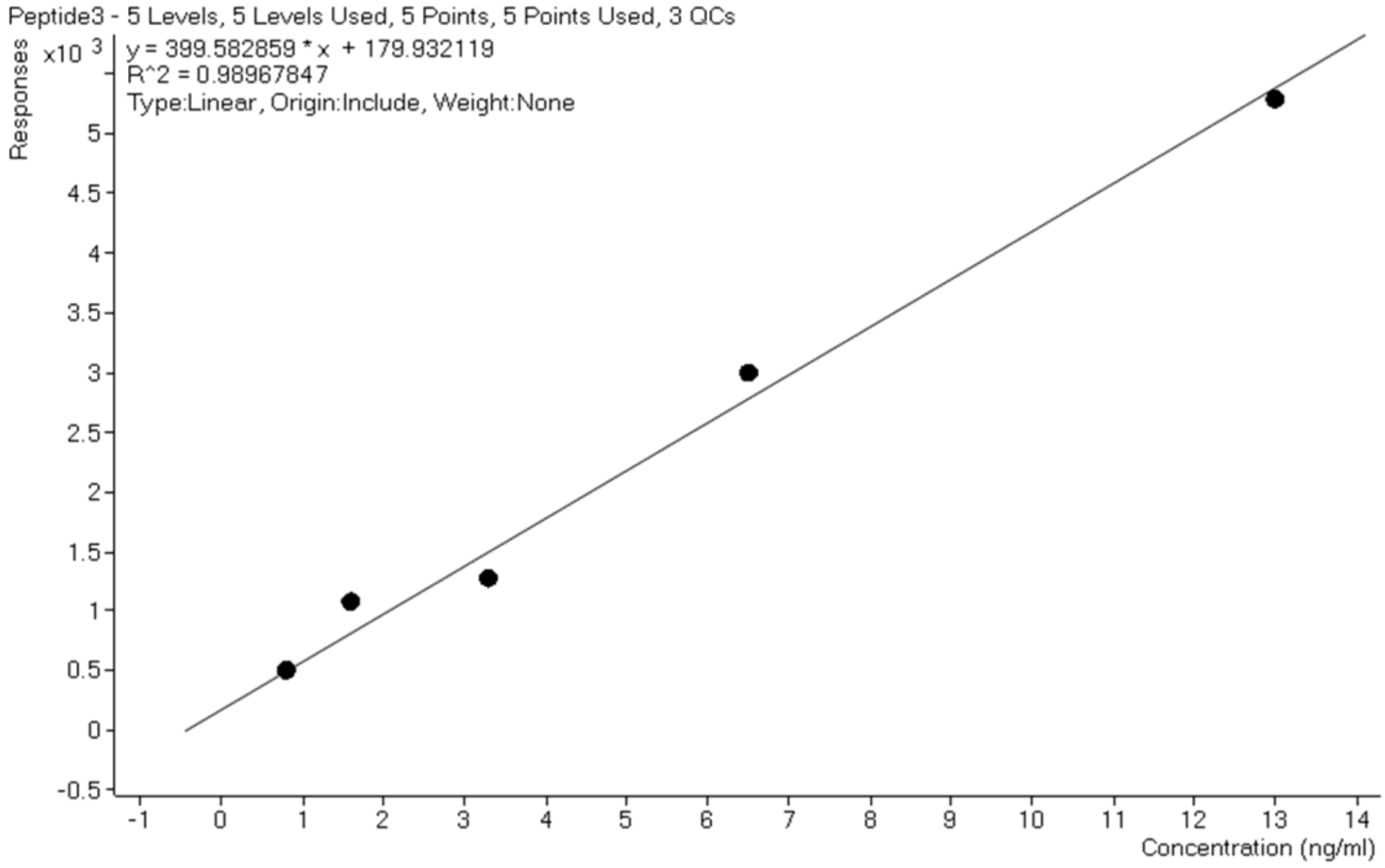

Gly-Pro

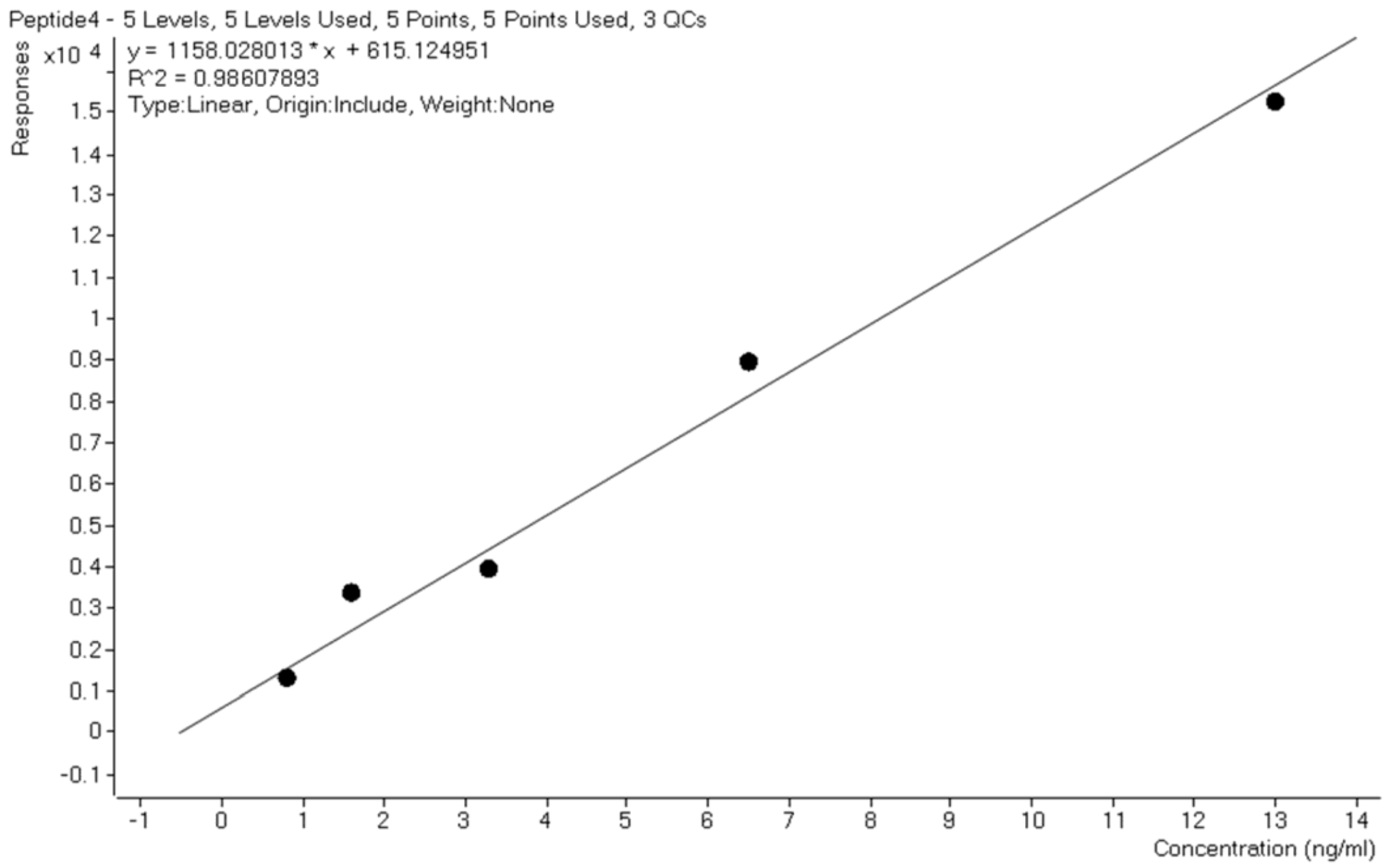

Gly-Pro-Hyp

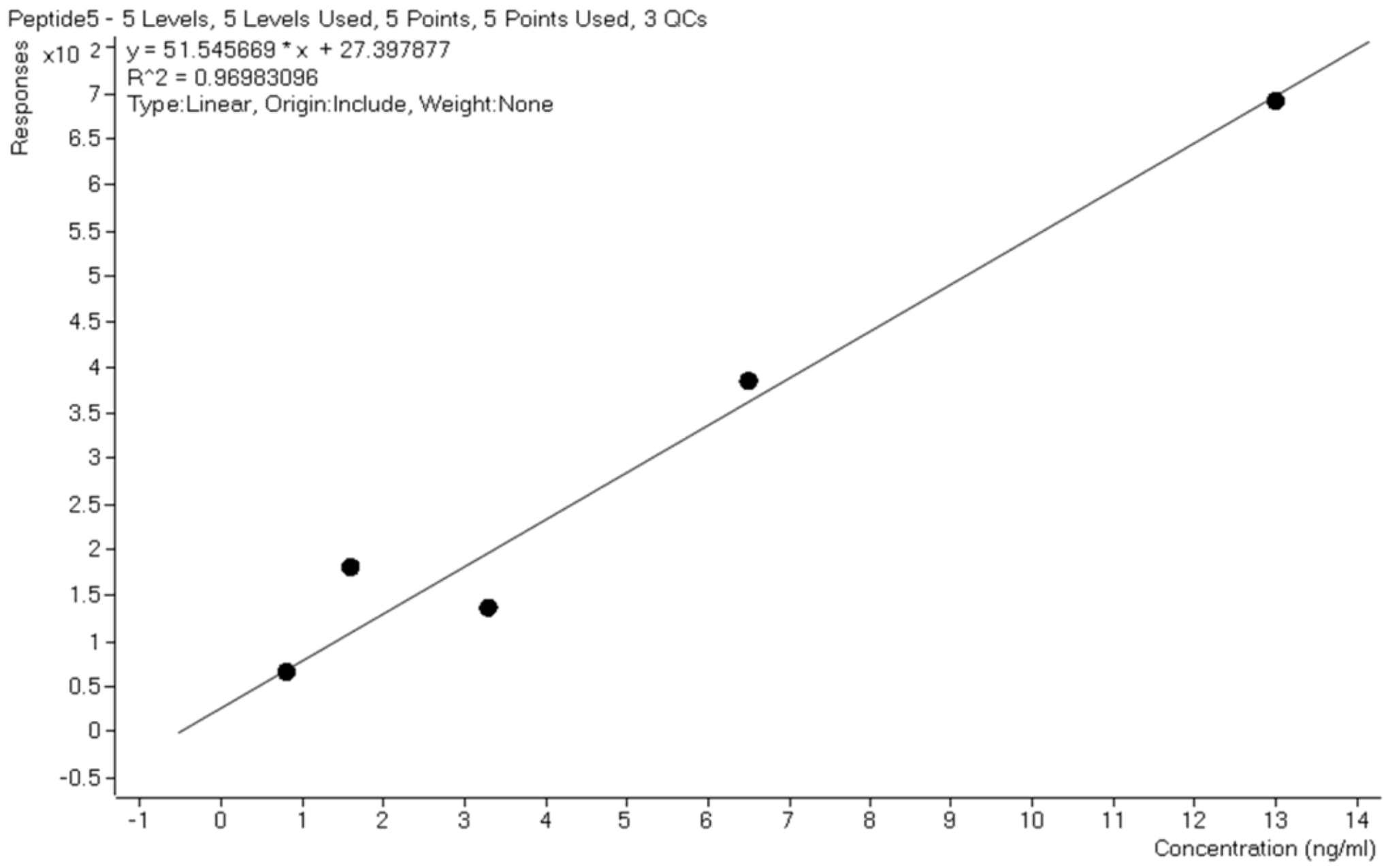

Ala-Hyp-Gly

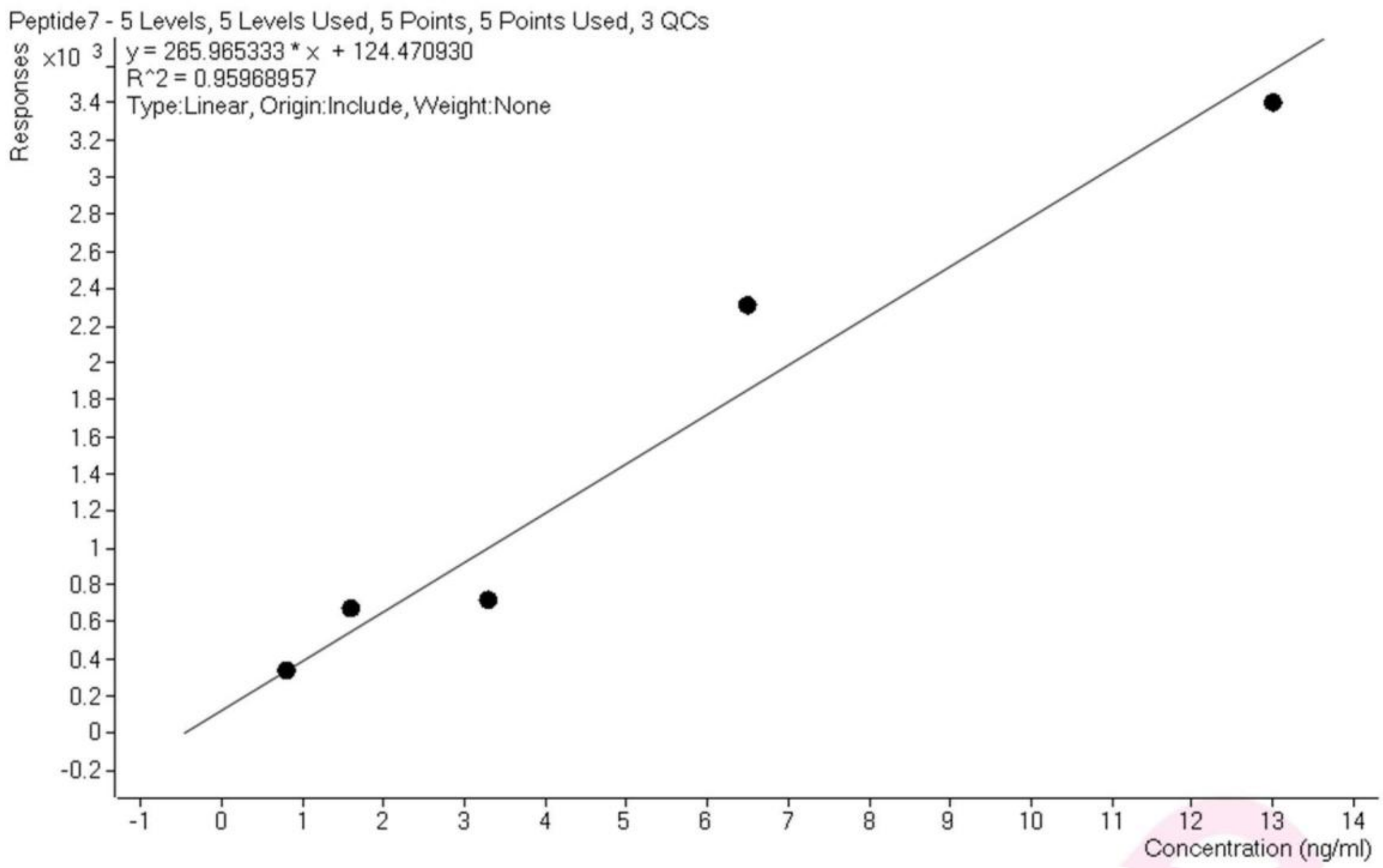

Ser-Hyp-Gly

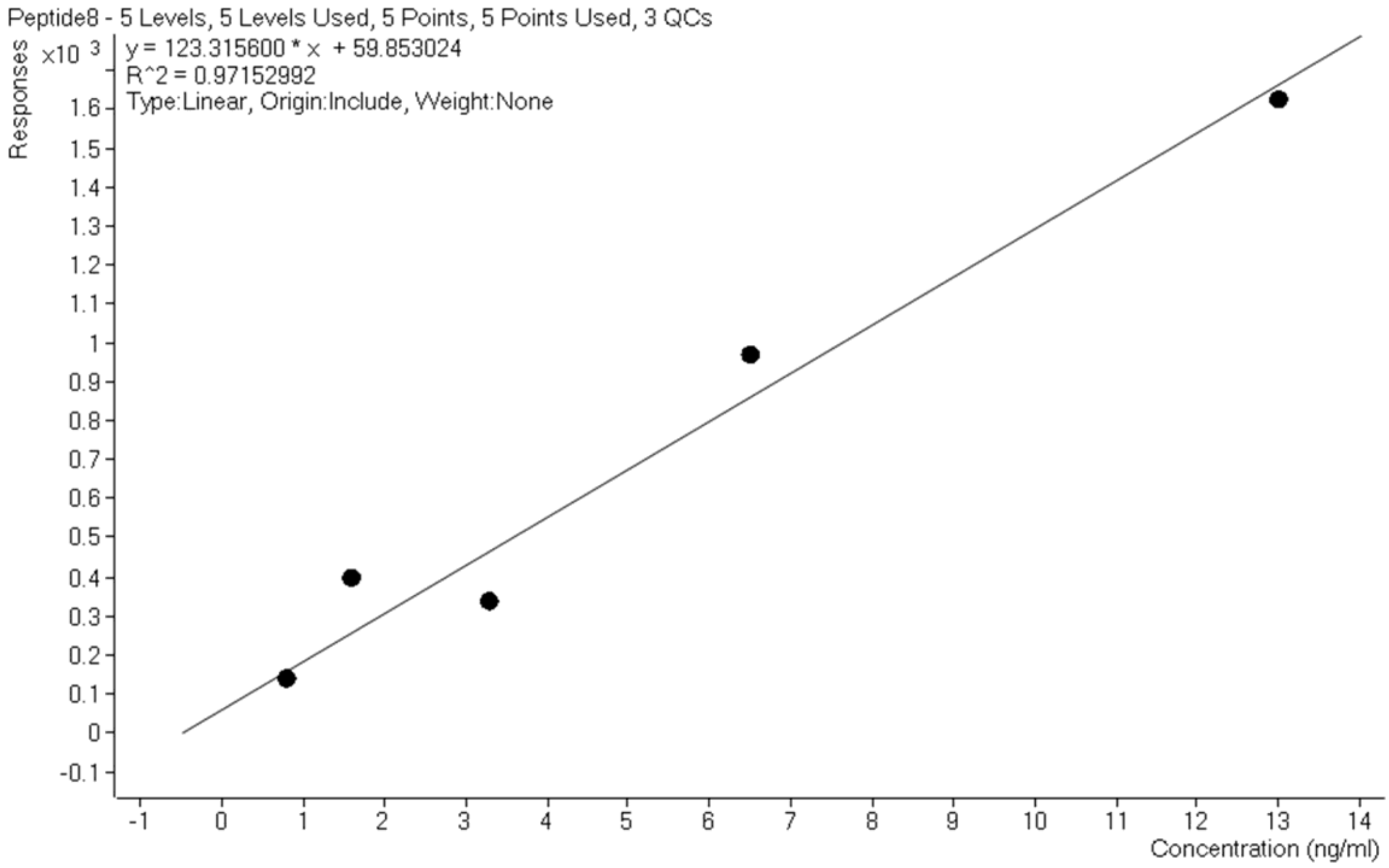

Peptide8 - 5 Levels, 5 Levels Used, 5 Points, 5 Points Used, 3 QCs
Responses
x10 3
y = 123.315600 * x + 59.853024
R^2 = 0.97152992
Type:Linear, Origin:Include, Weight:None
1.6
1.5
1.4
1.3
1.2
1.1
1
0.9
0.8
0.7
0.6
0.5
0.4
0.3
0.2
0.1
0
-0.1
-1
0
1
2
3
4
5
6
7
8
9
10
11
12
13
14
Concentration (ng/ml)

8 References

Al-Atif, H. (2022). Collagen Supplements for Aging and Wrinkles: A Paradigm Shift in the Fields of Dermatology and Cosmetics. Dermatology Practical & Conceptual, 12(1), e2022018. https://doi.org/10.5826/dpc.1201a18

Avila Rodríguez, M. I., Rodríguez Barroso, L. G., & Sánchez, M. L. (2018). Collagen: A review on its sources and potential cosmetic applications. Journal of Cosmetic Dermatology, 17(1), 20-26. https://doi.org/10.1111/jocd.12450

Bröker, M. E. E., Lalmahomed, Z. S., Roest, H. P., van Huizen, N. A., Dekker, L. J. M., Calame, W., Verhoef, C., IJzermans, J. N. M., & Luider, T. M. (2013). Collagen Peptides in Urine: A New Promising Biomarker for the Detection of Colorectal Liver Metastases. PLoS ONE, 8(8), e70918. https://doi.org/10.1371/journal.pone.0070918

Gallegos-Tintoré, S., Torres-Fuentes, C., Solorza-Feria, J., Alaíz Barragán, M., Girón-Calle, J., Martínez-Ayala, A. L., Chel Guerrero, L., & Vioque, J. (2015). Antioxidant and chelating activity of nontoxic Jatropha curcas L. protein hydrolysates produced by in vitro digestion using pepsin and pancreatin. https://doi.org/10.1155/2015/190129

Gao, J., Ma, Y., Guo, Z., Zhang, Y., Xing, F., Zhang, T., Kong, Y., Luo, X., Xu, L., & Zhang, G. (2023). Evaluating the Degradation Process of Collagen Sponge and Acellular Matrix Implants In Vivo Using the Standardized HPLC-MS/MS Method. Separations, 10(1), Article 1. https://doi.org/10.3390/separations10010047

Guo, S., Xu, X., Zhou, X., & Huang, Y. (2018). A rapid and simple UPLC-MS/MS method using collagen marker peptides for identification of porcine gelatin. RSC Advances, 8(7), 3768-3773. https://doi.org/10.1039/C7RA12539A

Heidenreich, E., Pfeffer, T., Kracke, T., Mechtel, N., Nawroth, P., Hoffmann, G. F., Schmitt, C. P., Hell, R., Poschet, G., & Peters, V. (2021). A Novel UPLC-MS/MS Method Identifies Organ-Specific Dipeptide Profiles. International Journal of Molecular Sciences, 22(18), 9979. https://doi.org/10.3390/ijms22189979

Ichikawa, S., Morifuji, M., Ohara, H., Matsumoto, H., Takeuchi, Y., & Sato, K. (2010). Hydroxyproline-containing dipeptides and tripeptides quantified at high concentration in human blood after oral administration of gelatin hydrolysate. International Journal of Food Sciences and Nutrition, 61(1), 52-60. https://doi.org/10.3109/09637480903257711

Ito, D., O'Brien, T. D., & Modiano, J. F. (2010). Exclusion of cytoplasmic fragments in flow cytometric analysis of lymph node samples from dogs with lymphoma using membrane-permeable violet laser-excitable DNA-binding fluorescent dye (DyeCycle Violet). Veterinary Clinical Pathology, 39(4), 494-498. https://doi.org/10.1111/j.1939-165X.2010.00252.x

Kumazawa, Y., Taga, Y., Iwai, K., & Koyama, Y. (2016). A Rapid and Simple LC-MS Method Using Collagen Marker Peptides for Identification of the Animal Source of Leather. Journal of Agricultural and Food Chemistry, 64(30), 6051-6057. https://doi.org/10.1021/acs.jafc.6b02132

Kumazawa, Y., Taga, Y., Takashima, M., & Hattori, S. (2018). A novel LC–MS method using collagen marker peptides for species identification of glue applicable to samples with multiple animal origins. Heritage Science, 6(1), 43.

https://doi.org/10.1186/s40494-018-0209-y
Li, Y., Wang, B., & Li, B. (2020). The in vitro bioavailability of anti-platelet peptides in collagen hydrolysate from silver carp (Hypophthalmichthys molitrix) skin. Journal of Food Biochemistry, 44(6), e13226. https://doi.org/10.1111/jfbc.13226
Liu, D., Nikoo, M., Boran, G., Zhou, P., & Regenstein, J. M. (2015). Collagen and gelatin. Annual Review of Food Science and Technology, 6, 527-557. https://doi.org/10.1146/annurev-food-031414-111800
Minekus, M., Alminger, M., Alvito, P., Ballance, S., Bohn, T., Bourlieu, C., Carrière, F., Boutrou, R., Corredig, M., Dupont, D., Dufour, C., Egger, L., Golding, M., Karakaya, S., Kirkhus, B., Le Feunteun, S., Lesmes, U., Macierzanka, A., Mackie, A., … Brodkorb, A. (2014). A standardised static in vitro digestion method suitable for food—An international consensus. Food & Function, 5(6), 1113-1124. https://doi.org/10.1039/c3fo60702j
Moumaris, M., Rajoelya, B., & Abuafa, N. (2015). Fluorescein Isothiocyanate-Dextran can Track Apoptosis and Necrosis Induced by Heat Shock of Peripheral Blood Mononuclear Cells and HeLa Cells. Open Biological Sciences Journal, 1(1). https://doi.org/10.2174/2352633501501010007
Pataridis, S., Eckhardt, A., Mikulíková, K., Sedláková, P., & Miksík, I. (2008). Identification of collagen types in tissues using HPLC-MS/MS. Journal of Separation Science, 31(20), 3483-3488. https://doi.org/10.1002/jssc.200800351
Quirós, A., del Mar Contreras, M., Ramos, M., Amigo, L., & Recio, I. (2009). Stability to gastrointestinal enzymes and structure-activity relationship of beta-casein-peptides with antihypertensive properties. Peptides, 30(10), 1848-1853. https://doi.org/10.1016/j.peptides.2009.06.031
Rohm, F., Daniel, H., & Spanier, B. (2019). Transport Versus Hydrolysis: Reassessing Intestinal Assimilation of Di- and Tripeptides by LC-MS/MS Analysis. Molecular Nutrition & Food Research, 63(21), e1900263. https://doi.org/10.1002/mnfr.201900263
Shimizu, K., Sato, M., Zhang, Y., Kouguchi, T., Takahata, Y., Morimatsu, F., & Shimizu, M. (2010). Molecular size of collagen peptide reverses the permeability of Caco-2 cells. Bioscience, Biotechnology, and Biochemistry, 74(5), 1123-1125. https://doi.org/10.1271/bbb.100015
Song, H., Tian, Q., & Li, B. (2020). Novel Hyp-Gly-containing antiplatelet peptides from collagen hydrolysate after simulated gastrointestinal digestion and intestinal absorption. Food & Function, 11(6), 5553-5564. https://doi.org/10.1039/D0FO00219D
Taga, Y., Kusubata, M., Ogawa-Goto, K., & Hattori, S. (2017). Identification of Collagen-Derived Hydroxyproline (Hyp)-Containing Cyclic Dipeptides with High Oral Bioavailability: Efficient Formation of Cyclo(X-Hyp) from X-Hyp-Gly-Type Tripeptides by Heating. Journal of Agricultural and Food Chemistry, 65(43), 9514-9521. https://doi.org/10.1021/acs.jafc.7b03714
Xu, J., Xing, F., Luo, X., Gao, J., Zhang, Y., Zhang, G., Bai, X., & Huang, C.-C. (2022). Quantitation of Collagen Type V in Tissues by High-Performance Liquid Chromatography Coupled to Mass Spectrometry. Tissue Engineering Part C: Methods, 28(3), 95-103. https://doi.org/10.1089/ten.tec.2022.0003
Yao, C., Rudnitzki, F., Hüttmann, G., Zhang, Z., & Rahmanzadeh, R. (2017). Important factors for cell-membrane permeabilization by gold nanoparticles activated by nanosecond-laser irradiation. International Journal of Nanomedicine, 12, 5659-5672. https://doi.org/10.2147/IJN.S140620

9 Registry and Regulation

The final report, raw data and the assay protocol have been saved in a digital format and on a paper copy. All the information provided from the client, and generated by Bionos Biotech will be considered as confidential. Information regarding materials, reagents and protocols adopted by Bionos Biotech SL during the assays is confidential and will not be shared with third parts.

The whole process involving this assay was performed under Quality Management System UNE-EN-ISO 9001/2015.

Article 20 of the EC Cosmetic Products Regulation 1223/2009 (CPR) frames the requirements for cosmetic claims. Furthermore, cosmetic claims have to comply with EU Regulation 655/2013 that provides the Common Criteria to ensure that the information conveyed to the end-users through claims is useful, understandable, and reliable so that consumers can make informed decisions.

The third Common Criterion, 'Evidential support', states that "claims for cosmetic products, whether explicit or implicit, shall be supported by adequate and verifiable evidence regardless of the types of evidential support used to substantiate them, including where appropriate expert assessments. Evidence for claim substantiation shall take into account the state-of-the-art practices. Where studies are being used as evidence, they shall be relevant to the product and the benefit claimed, shall follow well-designed, well-conducted methodologies (valid, reliable, and reproducible) and shall respect ethical considerations."

Experimental studies include (but are not limited to) studies in silico, in vitro, ex-vivo, with instrumental or biochemical methods, studies conducted on volunteers, investigator evaluations, sensory evaluations, etc. Different types of experimental studies can be used to provide data on the performance of cosmetic products. It is useful to take into consideration existing relevant guidelines, e.g., guidelines relating to instrumental clinical techniques, other European or international guidelines or standards (e.g., CEN, ISO, etc.).

Such studies should comprise methods which are reliable and reproducible. The studies should follow a well-designed and scientifically valid methodology according to best practices. The criteria used for evaluation of product performance should be defined with accuracy and chosen in accordance with the aim of the test. The experimental aspect of studies calls for reliance on knowledge and awareness of statistical principles in the design and analysis of the study, e.g., in terms of number of subjects, test samples, etc. This is necessary in order to ensure that the studies achieve scientifically and statistically valid conclusions.

A study protocol should be drawn up and validated in order to enable the study to be conducted

and monitored appropriately, thereby ensuring its quality. Whatever the type of study, it is important that the person conducting the study has the appropriate qualifications, has training and experience in the field of the proposed study and has high ethical qualitied standards and professional integrity.

Test facilities should maintain a quality assurance system, including standardised operating procedures. A monitoring system should be set up for each study in order to ensure that the protocol and the operating procedures are correctly followed.

Data processing and the interpretation of results should be fair and should not overstep the limits of the test's significance. Data recording, transformations, and representation in tabular or graphical form should be transparent or clearly explained if complex. It should not be designed to overstate the effect(s) measured. Appropriate statistical analysis of the data should be performed.
