# Supplementary material for: Enhanced digestion and absorption kinetics of ultra–low molecular weight collagen containing di- and tripeptides: evidence from human and cell models
Source: PeerJ. 2026 Aug 14;14:e21398. doi: 10.7717/peerj.21398 (PMC13480140; doi:10.7717/peerj.21398)
Supplement: Supplementary file 2 [file peerj-14-21398-s004.pdf]

PB Leiner, Part of Tessenderlo Group

IN VIVO EVALUATION OF THE BIOAVAILABILITY OF COLLAGEN FORMULATIONS (ULMWCP versus CP)

FINAL REPORT

DATE – December 9th, 2024

PB Leiner

Marius Duchéstraat 260,

1800 Vilvoorde,

Belgium

VAT: BE 0412 101 728

GOOD CLINICALPRACTICE
CERTIFICACIÓN ISO9001:2015
Nº EC-10595/21
LGAI Technologigcal Center, S.A. (Appus+) Certifies that the Quality Management System of the organization:
BIONOS BIOTECH, S.L.
For the following activites of:
To perform efficacy studies on products from the cosmetic and food industries. To perform biological diagnostic Assays.
Is in accordance with the requirements of the standard ISO 9001:2015
INITIAL CERTIFICATION DATE: 12/02/2021 THIS CERTIFICATE IS VALID UNTIL: 11/02/2027

C´REDIT D'IMPÔT RECHERCHE (CIR)
Direction Générale de la recherse et de l'innovation
DÉCISION D'AGRÉMENT
(Dépenses de recherche)
BIONOS
BIOTECH 46026
VALENCIA
Espagne Cet agrément est accordé au titre des années: 2023, 2024, 2025

INDEX

1 Title ....

2 Introduction...

3 Registration dates ....

4 Collagen formulations tested ....

5 Platform ....

6 Material and Methods...

7 Results ....

8 References ....

9 Registry and Regulation ....

1 Title

In vivo evaluation of the bioavailability of collagen formulations in human plasma.

2 Introduction

Collagen is a fibrous protein highly demanded in biomedical and cosmetic industries due to its benefits on the skin, biocompatibility, bioactivity, and weak immunogenicity (Avila Rodríguez et al., 2018). This biomaterial is composed of repetitive triplets of amino acids glycine (Gly), proline (Pro), and hydroxyproline (Hyp), and it is found to exert beneficial effects on several health benefits. Proteins in their native form such as collagen present low solubility in water due to its high molecular weight of approximately 300 kDa. These natural properties present difficulties in their application to cosmetics and edible products. However, the hydrolyzed collagen, which consists of segmented proteins with low molecular weight (Mw) between 0.3 kDa and 10 kDa, do not have this problem. The use of natural peptides like collagen hydrolysates has been widely utilized due to their excellent biocompatibility, easy biodegradability, and weak antigenicity (Liu et al., 2015). Recent evidence suggests that the consumption of amino-acids and/or peptides abundantly present in collagen may have the capacity to influence the synthesis of new collagen in the body (Al-Atif, 2022). Indeed, it has been found that collagen supplements improve skin moisture, elasticity, and hydration when orally administered (Al-Atif, 2022). Additionally, collagen reduces the wrinkling and roughness of the skin, and existing studies have not found any side effects of its oral supplements (Al-Atif, 2022).

3 Collagen formulations tested

The tested samples were received in Bionos on 18/11/2022 at room temperature and labelled

The products were stored at room temperature (RT) at Bionos Biotech facilities.

4 Registration dates

- Study begins: 09/12/2022.
- Study ends: 09/12/2024.
- Experimental phase begins: 18/01/2023.
- Experimental phase ends: 09/09/2024.

5 Platform

Volunteers consumed the product by oral intake following client's instructions.

6 Material and Methods

6.1 Analytical equipment

Inverted microscope, refrigerated centrifuge, thermoblock, micropipettes, serological pipettes, rack, laminar flow hood, vortex, freezer -80ºC, freezer -20ºC, UPLC-MS/MS 6460, CORTECS C18 column (2.1 x 150mm x 1.6µm).

6.2 Reagents

Ultrapure Water (Sigma), PBS (Gibco), formic acid (Sigma), acetonitrile (Sigma), DMSO (Sigma), ethanol (Sigma), methanol (Sigma), AccQ TagTM CP U derivatization reagent kit (Waters).

6.3 Analysis

The amino-acid quantification was performed using a methodology based on Waters' AccQ Tag method, widely used in the literature for the determination of amino-acids. Sample preparation consisted of protein precipitation by the addition of cold methanol and subsequent precolumn derivatization of the analytes, following the kit instructions. Once the sample was derivatized, it was injected into an Agilent 6460 UPLC-MS/MS. The chromatographic separation of the compounds was carried out using the CORTECS C18 column (2.1 x 150mm x 1.6µm).

The table indicates the amino acid sequence, solvent and stock concentration of the reference peptides used for calibration (GenScript). From these stock solutions, individual standards and multicomponent standards of 10 mg/L were prepared to fine-tune the method and prepare the calibration curve. In order to quantify the samples, a multicomponent calibration curve was prepared on a matrix in the indicated ranges.

6.4 Procedure

The study was a randomized, single-blinded trial to evaluate the bioavailability of collagen-derived amino-acids and peptides after the oral intake of different products. Twenty-five volunteers were enrolled in a 1-day study. Volunteers were divided in 5 groups, with 5 volunteers per group (4 groups corresponding to the tested products, 1 group corresponding to placebo). Trained personnel prepared drinkable solutions containing the study products in water. Control group (placebo) ingested approximately same amount of plain water. The amount of ingested product for each volunteer was calculated depending on their body weight (400 mg/kg of body weight). Drinkable solutions were provided to subjects right before consumption. After an 12h-fasting, volunteers ingested the placebo or products dissolved in water. Blood samples were taken from a digital puncture (non-invasive fingerstick procedure, by using VeriFine safety lancets 21G) before (0 min) and 15, 30, 120 and 240 minutes after

the oral intake of the product. Blood samples were collected in EDTA-containing tubes (Minicollect tube K3E EDTA), centrifuged at 1000 rpm, 5 minutes at 4ºC, and plasma (supernatant) was collected and stored at -20ºC. Digital attachment 1 shows the assignment of each volunteer to the different products. Volunteers were not allowed to drink water before or during the 4 hours of the study.

Use conditions

The product was ingested by oral intake by the volunteers following the client instructions (described above in the Procedure section).

The manufactured products by PB Leiner tested in this project are produced in compliance with:

- Regulation (EC) No 178/2002 of the European Parliament and of the council laying down the general principles and requirements of food law, establishing the European Food Safety Authority and laying down procedures in matters of food safety.
- Regulation (EC) No 853/2004 of the European Parliament and of the Council laying down specific hygiene rules for food of animal origin and amendments to this regulation.
- Commission Regulation (EC) No 2073/2005 on microbiological criteria for foodstuffs.

Panel

The panel represents the susceptible population to use the product. Inclusion criteria were:

- Healthy male volunteers to avoid any possible hormonal interference.
- Age is 30 years (between 25-45 years old (inclusive))
- Body weight is 70 kg (60-80 kg (inclusive) the range depending on the body mass index (BMI))
  - BMI in normal (healthy weight and overweight) range: 18.5 to 29.9 kg/m2 (Reference: WHO and CDC).
- Informed of the purpose and the protocol of the study and signed a written informed-consent form.
- Co-operative and available during the study period.
- Understanding and signature of Informed Consent .

On the other hand, the criteria of exclusion were:

- Diagnosis of allergies to gelatin, red meat (Alpha Gal Syndrome) or fish.
- Diagnosis of diseases about collagen metabolism such as Goodpasture syndrome, scleroderma, periarteritis nodosa or polyarteritis, dermatomyositis, and disseminated lupus erythematosus
- Diagnosis of following chronic Gastro-Intestinal Tract diseases blocking transfer of polypeptides from GIS to the blood such as: Peptic Ulcer, Duodenal Ulcer,Chronic Atrophic Gastritis, Helicobacter Pylori, Chronic Peptic Disorders, Chronic Acid Reflux = GER and GERD, Celiac Disease, Crohn's Disease, Irritable Bowel Syndrome, Lactose Intolerance, Diverticulitis, Diverticulosis.
- Diagnosis of any diseases such as cardiovascular disease, chronic kidney disease (CKD), gastrointestinal disorder, endocrinological disorder, immunological disorder, metabolic disease
- Consume more than the recommended alcohol guidelines i.e. >21 alcohol units/week for males and >14 units/week for females (europa.eu)
- Current smoking habit or history of smoking within the past 1 year.
- History of depression, schizophrenia, alcoholism, drug addiction, or mental illness.
- Current or previous intake of contraceptives, female hormones, obesity drugs, absorption inhibitors, antidepressants, or appetite suppressants.
- History of asthma or autoimmune disease Use of oral hormone therapies ie. cortisone or steroids in the 6 months prior to initiation of the study.
- Abnormal liver function or abnormal renal function.
- Women who had undergone, or planned to undergo, pregnancy or breastfeeding.
- Blood pressure >140/90 mmHg or hypertension with intake of a diuretic.
- No participation to another clinical trial in the last 6 months.
- No blood donation within a month.
- Problems with overall findings in blood-test results as determined by a specialist.
- Any condition judged by the investigator to be unsuitable for participation in the study.

Volunteers

The number of volunteers according to the client's needs was 25. Volunteer's data is found in Digital attachment 1, together with the assignment of each volunteer to the different products.

Study requirements

Requirements imposed on volunteers were the following:

- To respect the conditions of use.
- Do not use other food supplement products during the study period.
- Do not include any new nutricosmetic product in their daily routine.
- To maintain personal eating habits.
- Do not take drugs or dietary supplements, aimed at reducing weight or regulating gastrointestinal metabolic parameters during the study period.

Informed consent

Informed consent was obtained from each volunteer prior to initiating the study describing reasons for the study, possible adverse effects, associated risks and potential benefits of the treatment, and their limits of liability. The panelists signed and dated the informed consent document to indicate his authorization to proceed and acknowledge his understanding of the contents before the start of the study. The informed consent model is shown in Digital attachment 3.

Ethics

The study protocol is in accordance with the Scientific Committee on Consumer Safety (SCCS) guidance. It meets all international standards for research studies involving human subjects, the Good Clinical Practices (ICH-GCP), and World Medical Association. It has been conducted pursuant to the Declaration of Helsinki (1864), with the amendments of Tokyo (1975), Venice (1983), Hong Kong (1989), Somerset West (1996), Edinburgh (2000), Washington (2002), Tokyo (2004), Seoul (2008) and Fortaleza (2013). The standard protocol and test conditions were submitted to and approved by the Ethical Committee of Hospital Universitario y Politécnico La Fe

(Date 2022/11/30 and Code number 2022-920-1).

Checking of the acceptability

The subjects were requested to note any reaction observed, and sensation of discomfort felt. Together with the clinical examinations performed during the treatment, each subject was questioned by the responsible technician about the possible sensations of discomfort they felt, at the end of the study.

7 Results

7.1 In vivo bioavailability of collagen

The bioavailability of collagen-derived amino-acids in vivo was assessed by quantifying the free amino-acids present in the plasma of volunteers after the oral intake of the products at the indicated time points. For this, a subset of samples was first analyzed (pilot study), including a complete time-course (from time 0 min to 240 min) of Volunteer 10 and Volunteer 11 . Amino-acid levels in plasma were quantified as described in material and methods and represented in Table 1:

- In this table, "T0" indicates the amino-acid levels in plasma before the oral intake of the product (i.e., the basal amino-acid levels).
- For comparative purposes, the amino-acid quantification is expressed as percentage, detailed as follows:
  - The quantification of each amino-acid is represented as 100% in T0 (basal level).
  - For the rest of time points, the percentage of each amino-acid compared to T0 is shown.
  - Cells marked in red indicate that the percentage of the amino-acid in that time-point is higher than the basal level (T0).

Then, we performed the analysis of all the samples of the study. Table 2 summarizes the results involving all 25 volunteers. Volunteers were divided into 5 groups, corresponding to the placebo treatment and the 4 products; for each participant, 5 blood measurements were taken (0, 15, 30, 120 and 240 min). In table 2, cells marked in red indicate that the percentage of the amino-acid in that time-point is higher than the basal level (T0), while cells with bold black border represent significant values compared to the corresponding T0 value.

As Table 2 shows:

- A total of 23 different amino acids were detected in human plasma.
- For the placebo group, no increase in amino acid levels were observed during the tested time points, in accordance with the lack of amino acid intake.
- Oral intake of the high molecular weight collagen did not result in a significant increase of the level of any amino acid in plasma.
- In contrast, oral intake of an other special preparation significantly increased the hydroxyproline, arginine, glycine and proline at several time points.
- Most importantly, volunteers that ingested the ULMWC product presented an increase in the bioavailability of the collagen-derived amino acids hydroxyproline (after 30 min, 120 min and 240 min) and proline (30 min and 120 min).

Histidine	1,3-Methyl Histidine	Hydroxyproline	Arginine	Carnosine	Aspargine	Phosphoethanolamine	Phosphoserine	Anserine	Taurine	Serine	Ethanolamine	Glycine	Aspartic acid	Citrulline	Glutamic acid	Sarcosine	b-Alanine
100.0	nd	100.0	100.0	nd	100.0	nd	nd	nd	100.0	100.0	100.0	100	nd	100.0	100.0	nd	nd
79.4	#DIV/0!	52.9	80.3	#DIV/0!	64.5	#DIV/0!	#DIV/0!	#DIV/0!	93.8	70.7	91.5	80.0	#DIV/0!	73.4	98.1	#DIV/0!	#DIV/0!
82.0	#DIV/0!	58.6	83.0	#DIV/0!	74.4	#DIV/0!	#DIV/0!	#DIV/0!	92.7	63.7	91.1	84.6	#DIV/0!	83.4	97.7	#DIV/0!	#DIV/0!
78.2	#DIV/0!	57.3	80.5	#DIV/0!	74.7	#DIV/0!	#DIV/0!	#DIV/0!	99.9	72.0	85.5	80.4	#DIV/0!	67.8	96.2	#DIV/0!	#DIV/0!
80.0	#DIV/0!	67.5	108.8	#DIV/0!	94.1	#DIV/0!	#DIV/0!	#DIV/0!	108.4	84.5	94.8	94.6	#DIV/0!	106.4	114.1	#DIV/0!	#DIV/0!
100.0	nd	100.0	100.0	nd	100.0	nd	nd	nd	100.0	nd	100.0	100	nd	100.0	100.0	nd	nd
98.5	#DIV/0!	199.1	194.1	#DIV/0!	157.6	#DIV/0!	#DIV/0!	#DIV/0!	112.8	96.5	83.1	99.4	#DIV/0!	100.7	120.5	#DIV/0!	#DIV/0!
101.7	#DIV/0!	293.1	185.1	#DIV/0!	108.6	#DIV/0!	#DIV/0!	#DIV/0!	116.0	90.7	105.9	135.1	#DIV/0!	116.0	101.7	#DIV/0!	#DIV/0!
89.1	#DIV/0!	216.0	89.7	#DIV/0!	123.5	#DIV/0!	#DIV/0!	#DIV/0!	129.5	104.1	103.6	106.1	#DIV/0!	94.3	124.4	#DIV/0!	#DIV/0!
101.0	#DIV/0!	348.5	75.1	#DIV/0!	207.4	#DIV/0!	#DIV/0!	#DIV/0!	131.6	135.2	97.0	126.4	#DIV/0!	116.4	131.6	#DIV/0!	#DIV/0!
100.0	nd	100.0	100.0	nd	100.0	nd	nd	nd	100.0	100.0	100.0	100	nd	100.0	100.0	nd	nd
133.0	#DIV/0!	345.6	408.7	#DIV/0!	153.8	#DIV/0!	#DIV/0!	#DIV/0!	107.2	103.3	82.7	199.4	#DIV/0!	90.1	103.0	#DIV/0!	#DIV/0!
128.9	#DIV/0!	717.3	521.8	#DIV/0!	138.8	#DIV/0!	#DIV/0!	#DIV/0!	106.0	118.3	101.7	211.0	#DIV/0!	119.0	118.4	#DIV/0!	#DIV/0!
107.8	#DIV/0!	1057.4	301.9	#DIV/0!	172.0	#DIV/0!	#DIV/0!	#DIV/0!	112.8	119.1	85.8	207.6	#DIV/0!	89.5	107.6	#DIV/0!	#DIV/0!
139.9	#DIV/0!	773.0	192.1	#DIV/0!	165.6	#DIV/0!	#DIV/0!	#DIV/0!	112.3	124.3	88.3	135.7	#DIV/0!	104.1	135.8	#DIV/0!	#DIV/0!
100.0	nd	100.0	100.0	nd	100.0	nd	nd	nd	100.0	nd	100.0	100	nd	100.0	100.0	nd	nd
89.0	#DIV/0!	549.6	294.3	#DIV/0!	100.8	#DIV/0!	#DIV/0!	#DIV/0!	86.6	91.6	92.9	197.9	#DIV/0!	83.9	75.4	#DIV/0!	#DIV/0!
81.4	#DIV/0!	1116.6	390.2	#DIV/0!	135.3	#DIV/0!	#DIV/0!	#DIV/0!	108.2	102.8	102.2	264.2	#DIV/0!	91.3	90.6	#DIV/0!	#DIV/0!
63.8	#DIV/0!	1437.0	161.1	#DIV/0!	103.4	#DIV/0!	#DIV/0!	#DIV/0!	102.2	99.1	96.7	180.2	#DIV/0!	78.1	75.8	#DIV/0!	#DIV/0!
73.3	#DIV/0!	835.0	134.5	#DIV/0!	84.6	#DIV/0!	#DIV/0!	#DIV/0!	95.7	78.2	83.9	119.2	#DIV/0!	78.5	87.7	#DIV/0!	#DIV/0!
100.0	nd	100.0	100.0	nd	100.0	nd	nd	nd	100.0	100.0	100.0	100	nd	100.0	100.0	nd	nd
74.9	#DIV/0!	196.1	111.6	#DIV/0!	63.3	#DIV/0!	#DIV/0!	#DIV/0!	62.8	70.6	83.5	97.3	#DIV/0!	57.4	54.2	#DIV/0!	#DIV/0!
75.6	#DIV/0!	439.1	173.3	#DIV/0!	80.8	#DIV/0!	#DIV/0!	#DIV/0!	73.5	88.1	84.9	135.0	#DIV/0!	63.5	69.3	#DIV/0!	#DIV/0!
86.6	#DIV/0!	636.3	99.8	#DIV/0!	89.1	#DIV/0!	#DIV/0!	#DIV/0!	79.5	100.5	82.2	100.4	#DIV/0!	72.4	67.1	#DIV/0!	#DIV/0!
107.2	#DIV/0!	425.0	102.0	#DIV/0!	76.0	#DIV/0!	#DIV/0!	#DIV/0!	73.5	65.4	64.6	70.7	#DIV/0!	60.0	65.8	#DIV/0!	#DIV/0!

Threonine	Hydroxylysine	Alanine	GammaaminoButyricAc	Aminoadipic acid	Ornithine	Proline	b-Amino isobutyric acid	Cystathionine	Lysine	Cystine	AlfaaminoButyricAcid	Tyrosine	Methionine	Valine	Homocystine	Isoleucine	Leucine	Phenylalanine	Tryptophan
100.0	nd	100.0	nd	nd	100.0	100.0	nd	nd	100.0	nd	100.0	100.0	100.0	100.0	nd	100.0	100	100	100
78.7	#DIV/0!	83.4	#DIV/0!	#DIV/0!	78.2	83.0	#DIV/0!	#DIV/0!	88.5	#DIV/0!	86.7	81.9	82.5	85.4	#DIV/0!	82.3	82.2	87.8	94.1
78.0	#DIV/0!	78.3	#DIV/0!	#DIV/0!	73.0	86.9	#DIV/0!	#DIV/0!	79.5	#DIV/0!	89.6	78.1	86.1	83.9	#DIV/0!	78.3	82.3	88.6	93.3
75.8	#DIV/0!	83.0	#DIV/0!	#DIV/0!	78.8	87.0	#DIV/0!	#DIV/0!	83.6	#DIV/0!	80.5	75.5	79.1	78.8	#DIV/0!	76.7	78.0	85.0	87.8
89.2	#DIV/0!	88.7	#DIV/0!	#DIV/0!	89.3	101.4	#DIV/0!	#DIV/0!	101.8	#DIV/0!	95.6	82.6	94.2	92.1	#DIV/0!	96.8	96.7	95.4	92.0
100.0	nd	100.0	nd	nd	100.0	100.0	nd	nd	100.0	nd	100.0	100.0	100.0	100.0	nd	100.0	100.0	100.0	100.0
110.4	#DIV/0!	110.4	#DIV/0!	#DIV/0!	102.9	120.3	#DIV/0!	#DIV/0!	132.8	#DIV/0!	92.4	110.4	134.9	112.7	#DIV/0!	119.2	115.5	108.8	114.8
99.0	#DIV/0!	108.6	#DIV/0!	#DIV/0!	89.3	149.7	#DIV/0!	#DIV/0!	113.8	#DIV/0!	108.6	91.8	97.3	106.5	#DIV/0!	95.2	101.6	96.2	84.5
104.1	#DIV/0!	98.0	#DIV/0!	#DIV/0!	67.6	99.9	#DIV/0!	#DIV/0!	85.8	#DIV/0!	104.0	86.4	83.4	98.8	#DIV/0!	94.7	94.6	79.1	92.9
131.5	#DIV/0!	120.8	#DIV/0!	#DIV/0!	119.8	143.9	#DIV/0!	#DIV/0!	124.2	#DIV/0!	103.6	93.2	107.8	109.5	#DIV/0!	104.5	105.0	93.8	97.6
100.0	nd	100.0	nd	nd	100.0	100.0	nd	nd	100.0	nd	101.0	nd	100.0	100.0	nd	100.0	100.0	100.0	100.0
109.1	#DIV/0!	127.1	#DIV/0!	#DIV/0!	132.2	194.3	#DIV/0!	#DIV/0!	139.2	#DIV/0!	145.0	113.1	106.2	120.3	#DIV/0!	139.1	140.7	138.0	98.8
125.7	#DIV/0!	154.2	#DIV/0!	#DIV/0!	153.6	259.8	#DIV/0!	#DIV/0!	173.8	#DIV/0!	142.3	110.8	102.7	138.2	#DIV/0!	149.2	147.0	125.4	85.5
120.1	#DIV/0!	131.1	#DIV/0!	#DIV/0!	115.1	296.8	#DIV/0!	#DIV/0!	141.8	#DIV/0!	139.9	96.8	107.8	114.1	#DIV/0!	117.5	121.5	109.5	81.1
109.1	#DIV/0!	119.2	#DIV/0!	#DIV/0!	134.7	206.1	#DIV/0!	#DIV/0!	141.6	#DIV/0!	123.8	97.1	103.9	104.8	#DIV/0!	99.9	100.0	105.0	88.8
100.0	nd	100.0	nd	nd	100.0	100.0	nd	nd	100.0	nd	101.0	100.0	100.0	100.0	nd	100.0	100.0	100.0	100.0
98.3	#DIV/0!	114.0	#DIV/0!	#DIV/0!	127.6	165.3	#DIV/0!	#DIV/0!	96.4	#DIV/0!	122.6	99.5	150.9	103.2	#DIV/0!	114.4	122.1	120.3	94.1
121.2	#DIV/0!	139.9	#DIV/0!	#DIV/0!	136.9	229.9	#DIV/0!	#DIV/0!	123.2	#DIV/0!	145.0	97.8	160.7	109.3	#DIV/0!	111.0	121.2	113.2	84.2
99.5	#DIV/0!	106.7	#DIV/0!	#DIV/0!	101.5	231.4	#DIV/0!	#DIV/0!	80.7	#DIV/0!	125.5	85.7	120.0	100.7	#DIV/0!	93.6	101.4	98.6	76.8
79.7	#DIV/0!	74.8	#DIV/0!	#DIV/0!	81.8	158.7	#DIV/0!	#DIV/0!	71.1	#DIV/0!	112.9	79.2	97.2	94.5	#DIV/0!	93.1	93.6	96.4	80.4
100.0	nd	100.0	nd	nd	100.0	100.0	nd	nd	100.0	nd	102.0	100.0	100.0	100.0	nd	100.0	100.0	100.0	100.0
71.5	#DIV/0!	83.5	#DIV/0!	#DIV/0!	97.0	139.3	#DIV/0!	#DIV/0!	65.6	#DIV/0!	81.7	82.4	107.9	79.6	#DIV/0!	88.9	93.9	97.1	75.9
89.4	#DIV/0!	105.1	#DIV/0!	#DIV/0!	121.8	196.9	#DIV/0!	#DIV/0!	92.1	#DIV/0!	102.6	81.2	123.7	83.7	#DIV/0!	89.8	98.1	99.6	73.1
82.5	#DIV/0!	82.2	#DIV/0!	#DIV/0!	143.6	196.1	#DIV/0!	#DIV/0!	83.3	#DIV/0!	102.3	74.2	92.9	79.4	#DIV/0!	78.1	86.0	87.5	67.5
61.7	#DIV/0!	64.9	#DIV/0!	#DIV/0!	97.5	142.8	#DIV/0!	#DIV/0!	72.8	#DIV/0!	90.9	61.1	76.0	69.7	#DIV/0!	66.9	71.8	79.2	71.6

in plasma before the oral intake of the product (i.e., the basal amino-acid levels). Results are represented as percentage, where the quantification of each amino-acid is represented as 100% in T0 (basal level). Cells marked in red indicate that the percentage of the amino-acid in that time-point is higher than the basal level (T0). Amino acid names marked in yellow represent major collagen peptides. Amino acid names marked in red represent essential amino acids. Names not marked in red represent non-essential peptides. Percentages with bold black border represent significant values, compared to the corresponding T0 value. Statistics detailed in attachment 1.

We then examined the levels of the main collagen amino acids collectively; proline, hydroxyproline and glycine, observing different absorption dynamics for each product (Figure 1). As expected, in the Placebo group, there was no significant increase in collagen-related amino acids across the time points, indicating minimal impact on bioavailability. In contrast, CP U/O , and ULMWC groups showed significant increases in proline, hydroxyproline, and glycine levels, particularly between 15 and 120 minutes after the oral intake. These results indicate that these products enhance the absorption and bioavailability of the main collagen-derived amino acids.

In addition, we represented the plasma levels of the total amino acids, the non-essential amino acids and the essential amino acids, in all assessed time points (Figure 2). As Figure 2 shows, no statistically significant differences in the bioavailability of these groups of amino acids were found after the oral intake of the products when compared to the basal levels.

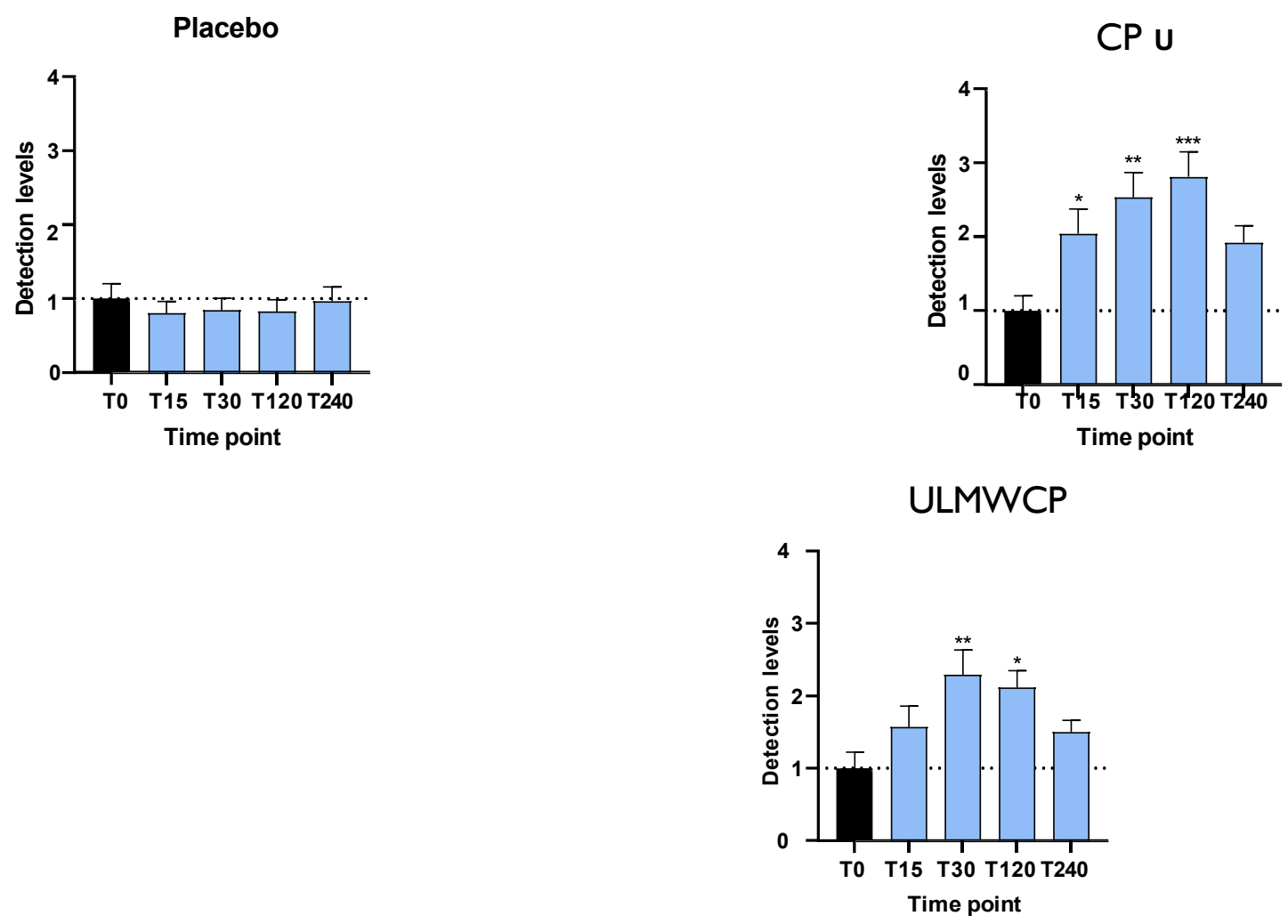

Figure 1. Representation of the normalized proline, hydroxyproline and glycine levels in plasma of volunteers at the assessed time-points after the oral intake of the products. T0 indicates the amino-acid levels in plasma before the oral intake of the product (i.e., the basal amino-acid levels). Results are normalized to T0 (basal level). Asterisks indicate significant differences

*compared to T0: * p-value < 0.05, ** p-value < 0.01, *** p-value < 0.001.*

7.2 In vivo bioavailability of collagen-derived peptides (Ongoing methodology preliminary work)

Collagen-derived peptides in vitro were assessed by quantifying the selected di- and tri-peptides (Pro-Hyp, Hyp-Gly, Ala-Hyp, Gly-Pro, Gly-Pro-Hyp, Pro-Hyp-Gly, Ala-Hyp-Gly and Ser-Hyp-Gly).

First, the detection of the purified standards of each peptide was optimized in the UPLC-MS/MS system. For each peptide, below is shown a representative chromatogram of the analysis, indicating the acquisition time for each of them. In addition, for each peptide, a graph showing the corresponding mass-to-charge (m/z) ratio is also included, indicating the transition points in the top of the graph. First, the detection of the purified standards of each peptide was optimized in the UPLC-MS/MS system. For each peptide, below is shown a representative chromatogram of the analysis, indicating the acquisition time for each of them. This part is an ongoing project

Hyp-Gly

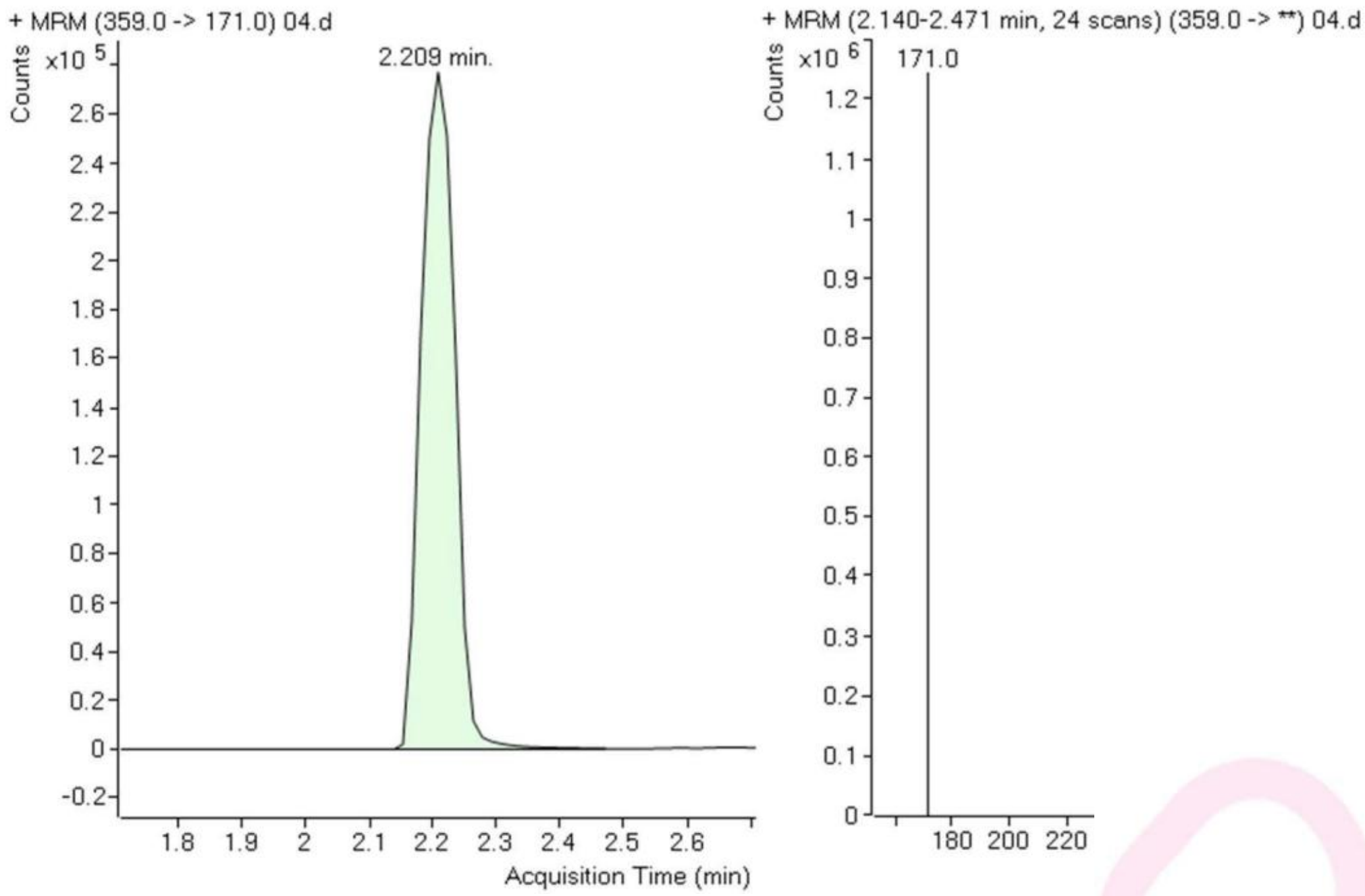

Ala-Hyp

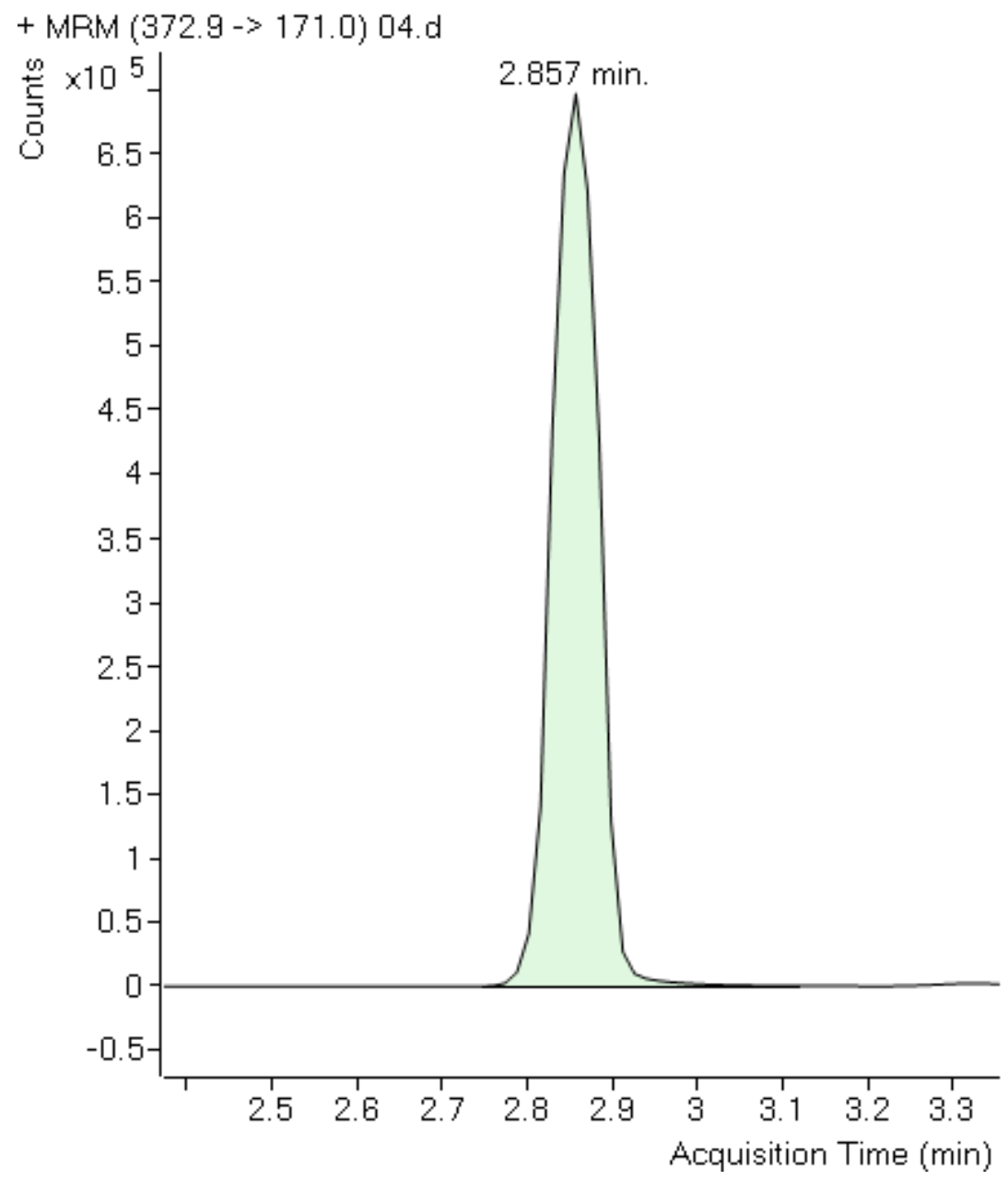

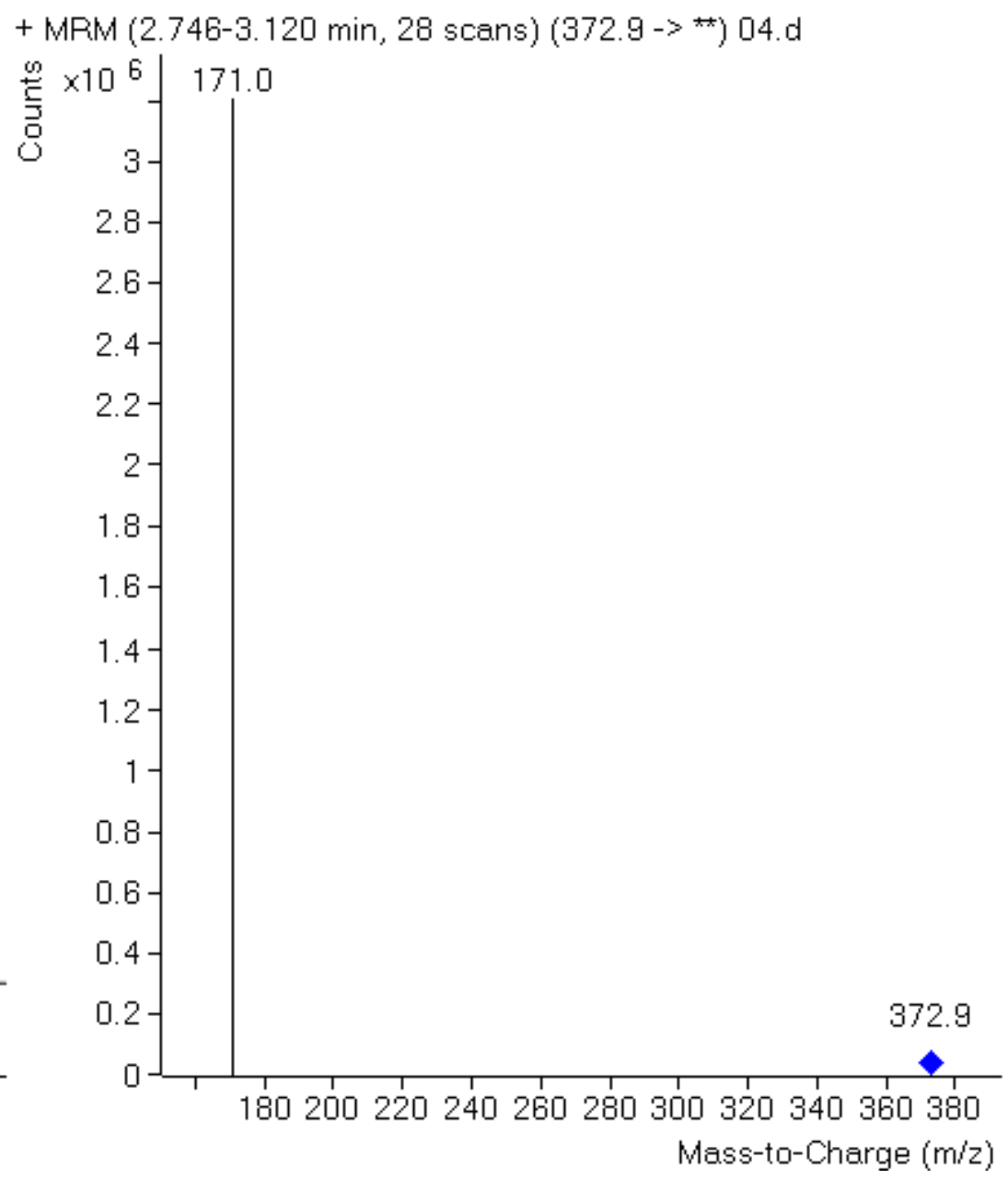

Gly-Pro

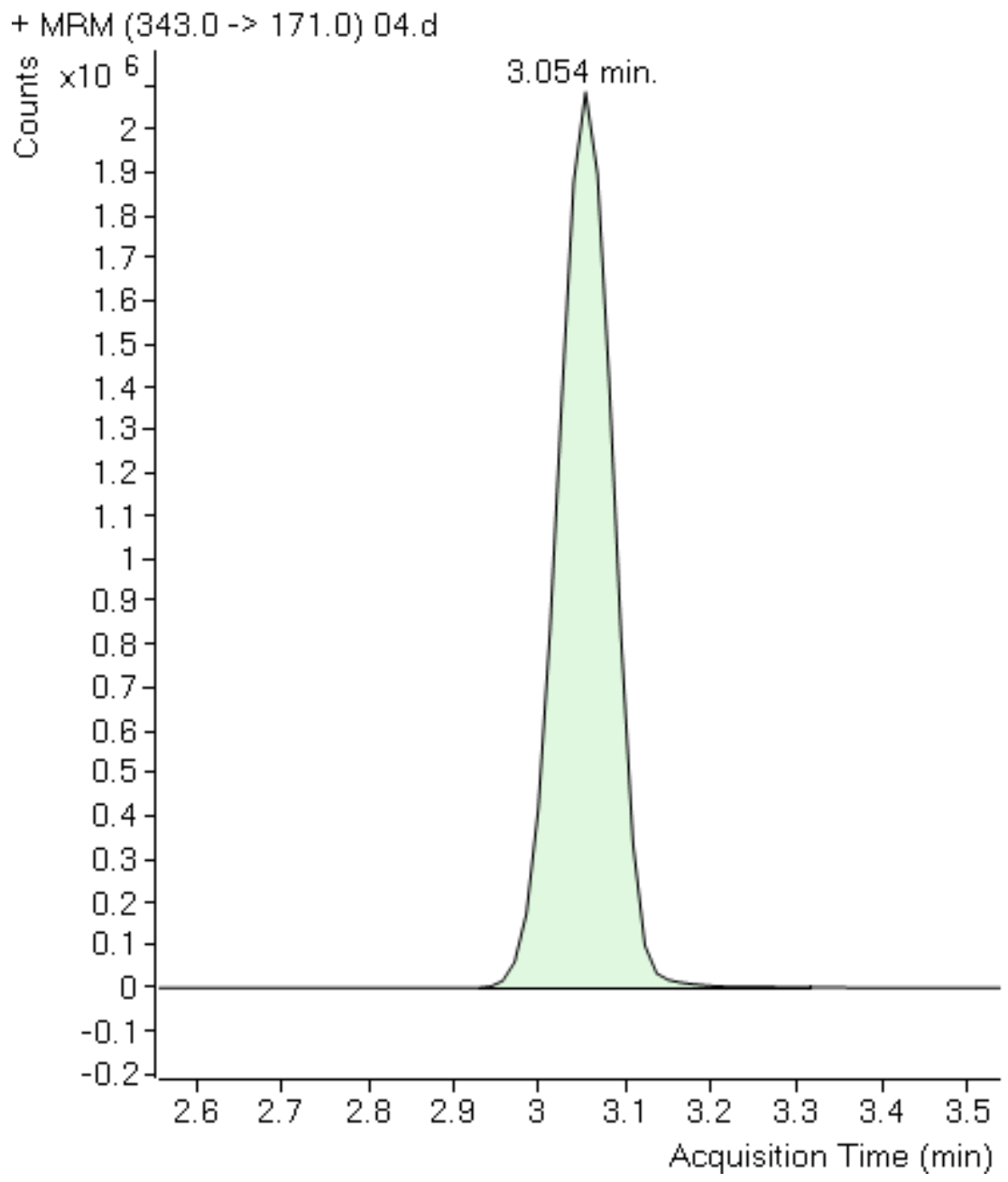

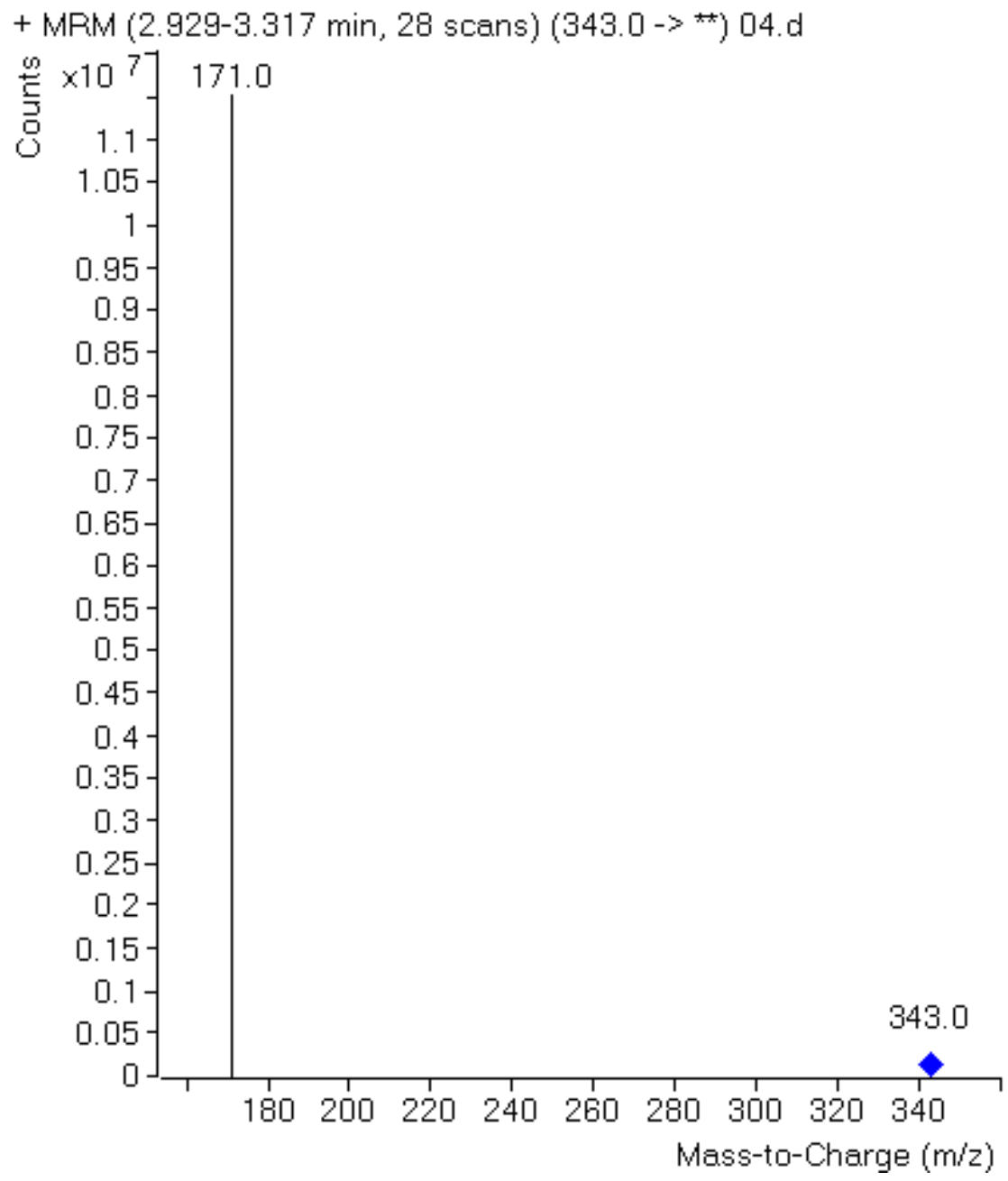

Gly-Pro-Hyp

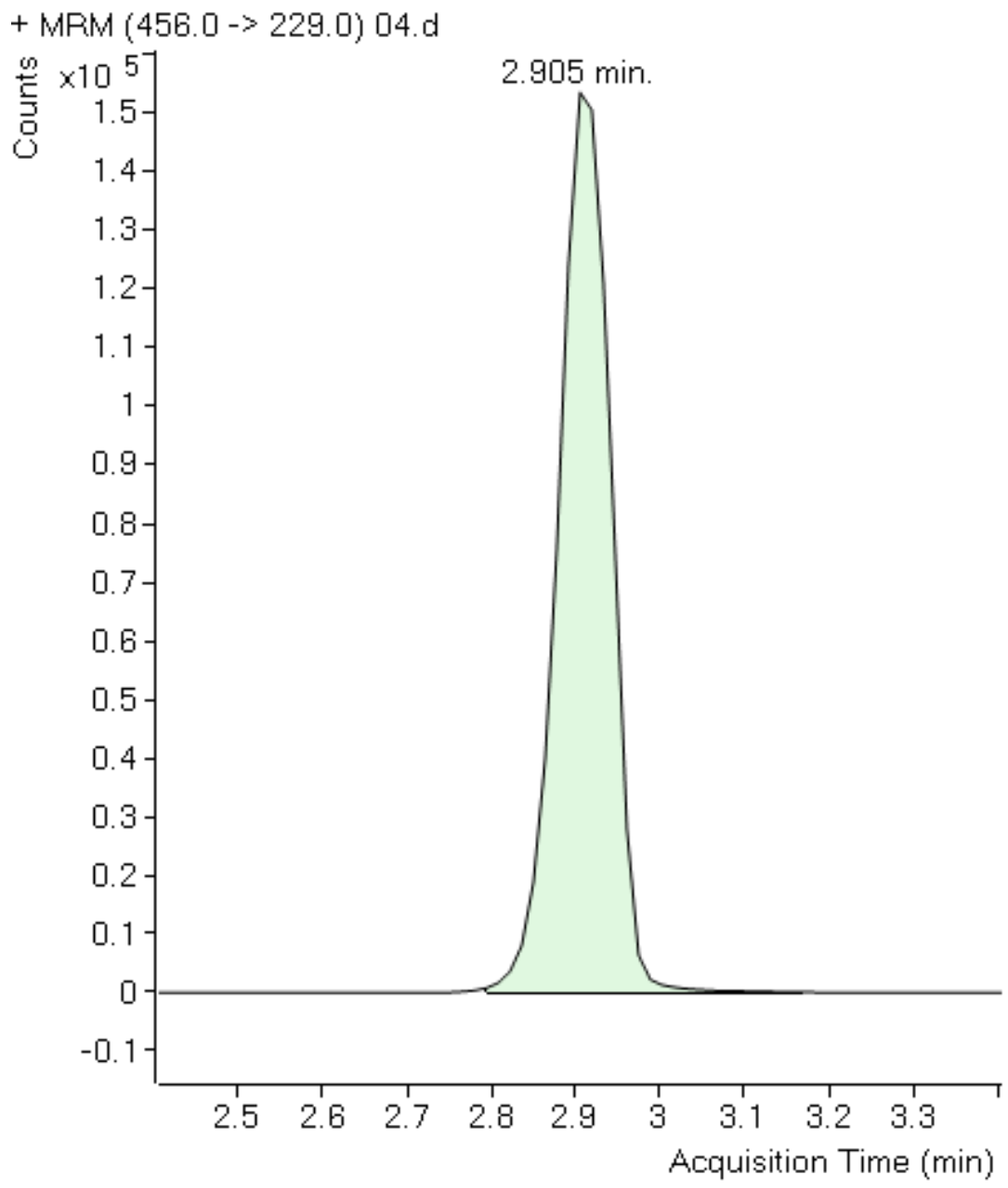

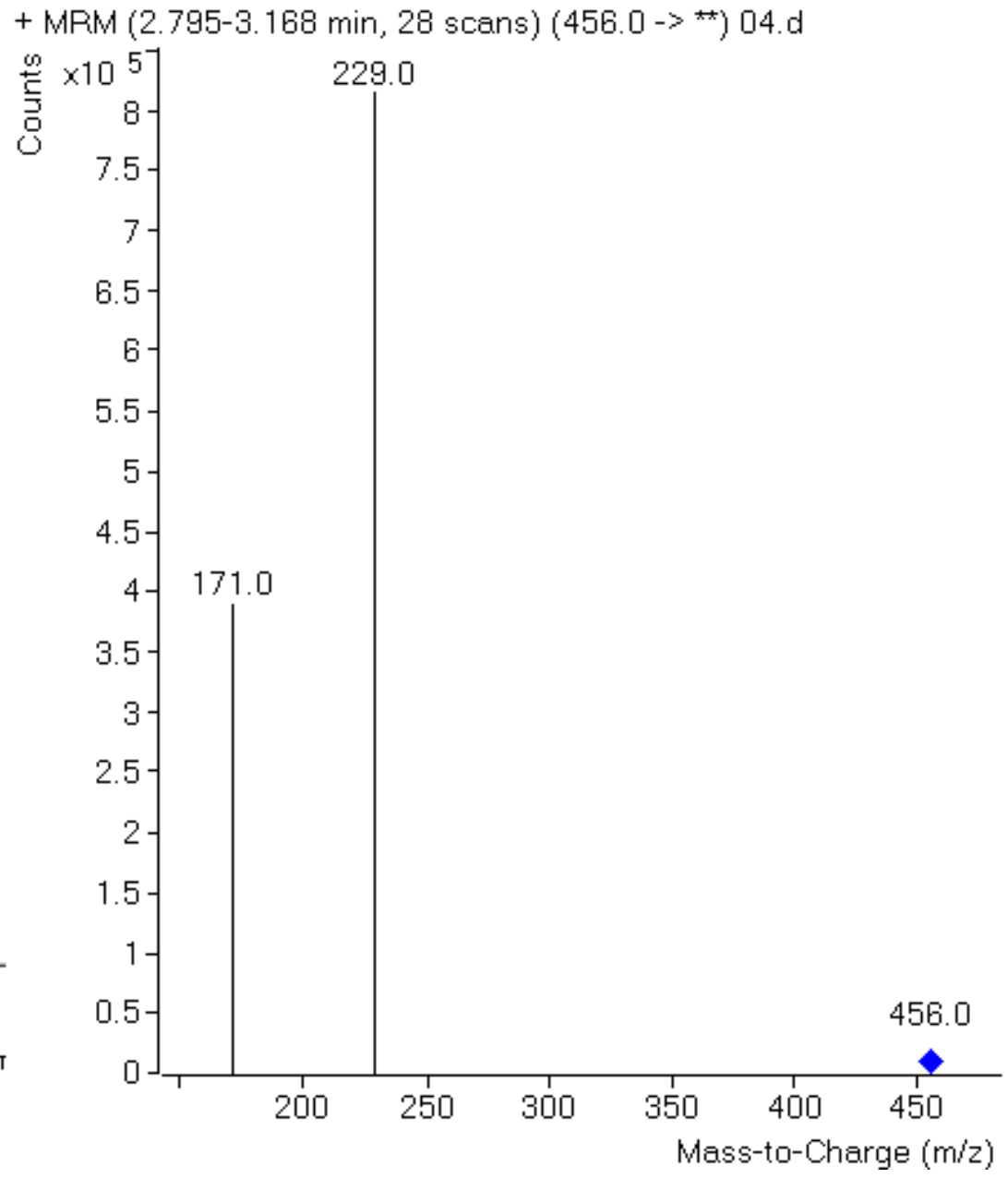

Ala-Hyp-Gly

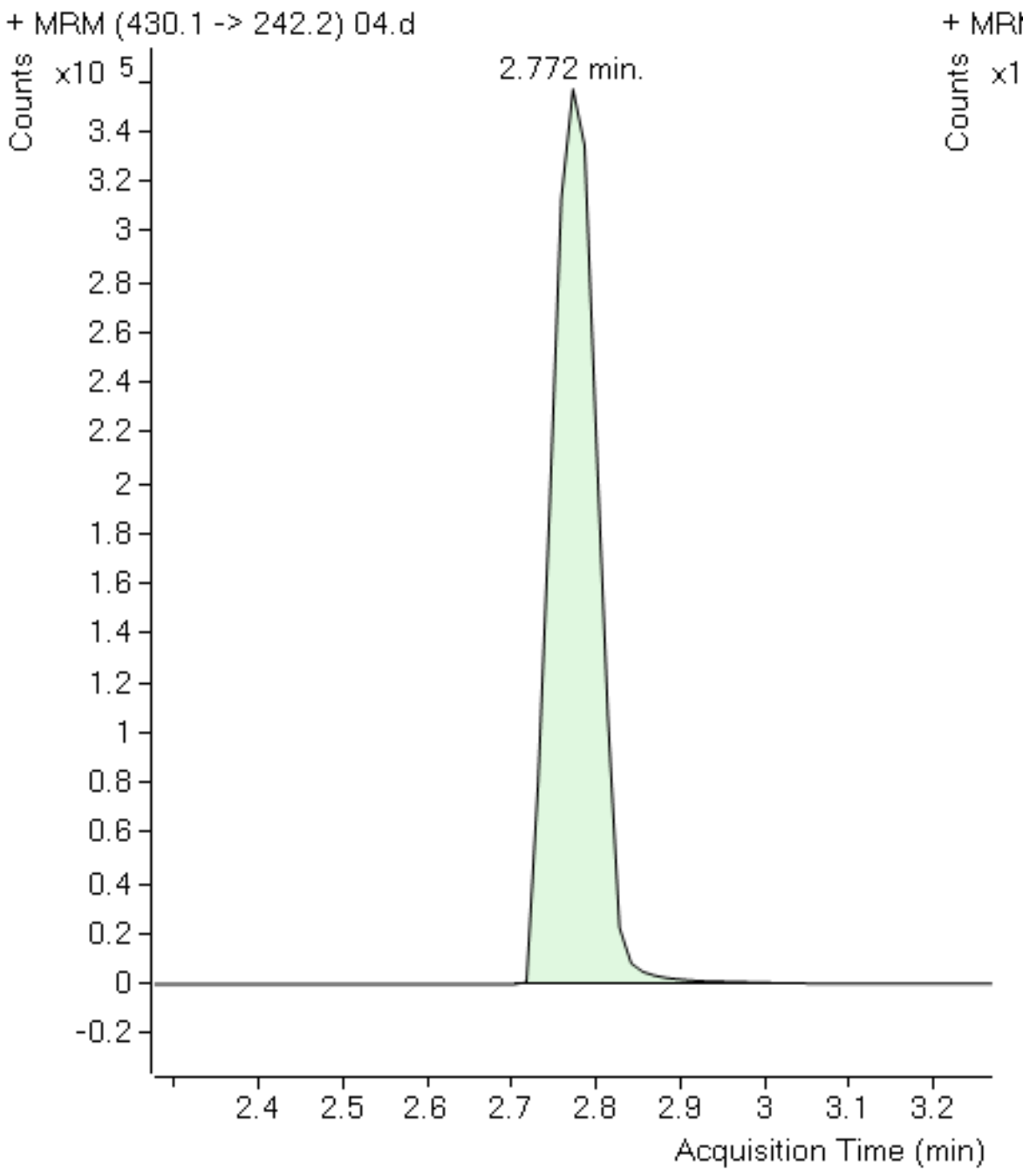

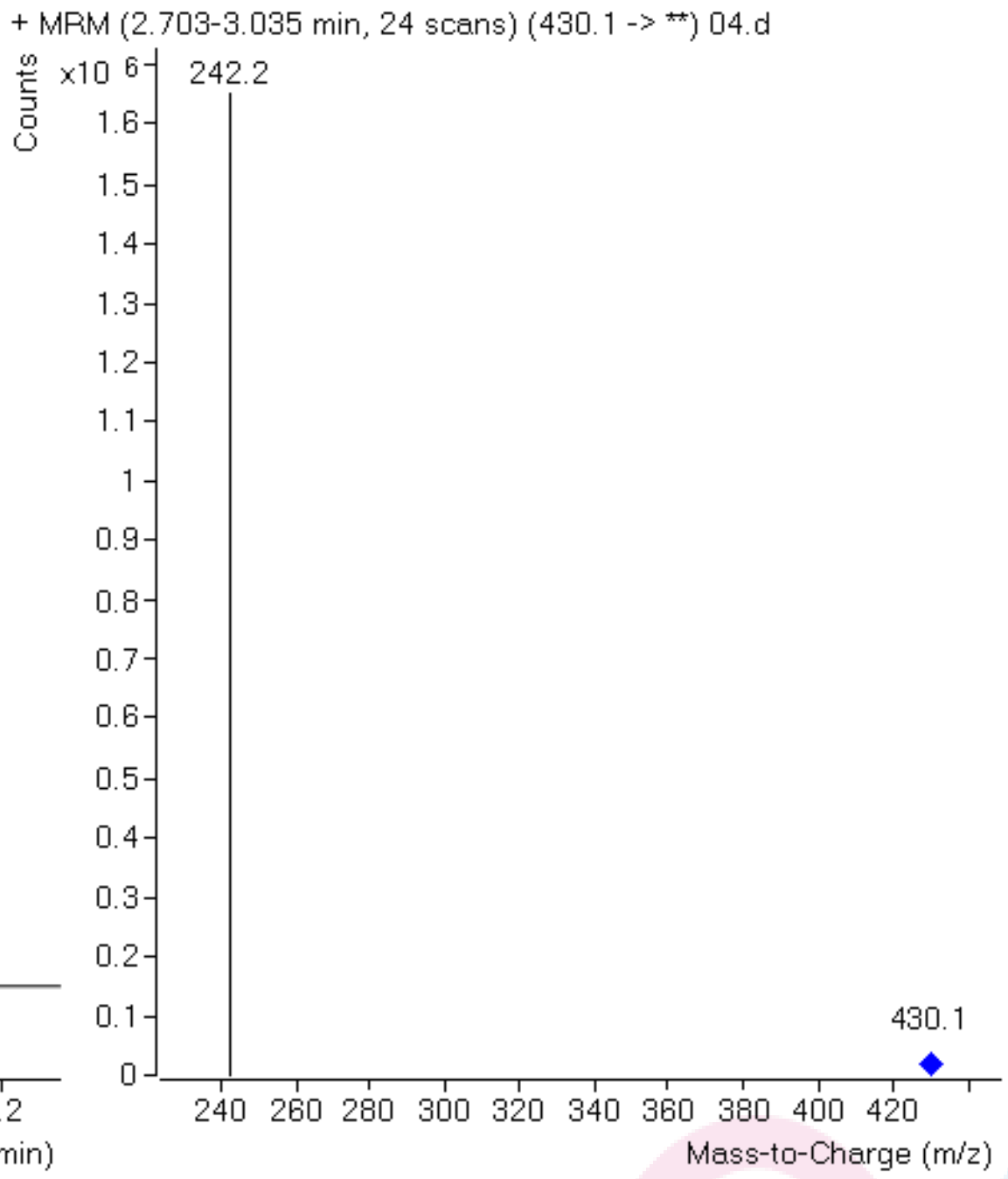

Ser-Hyp-Gly

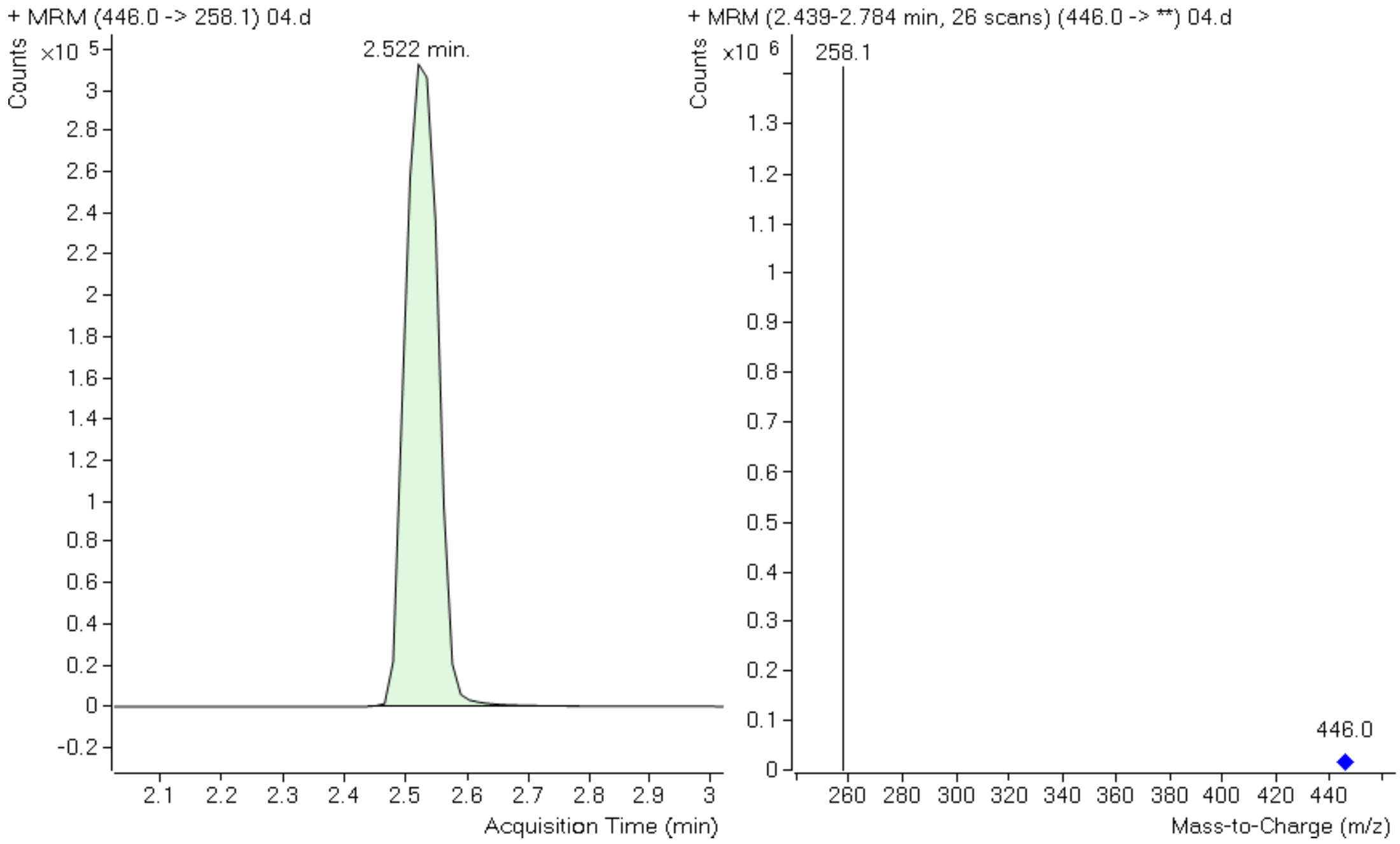

Pro-Hyp-Gly

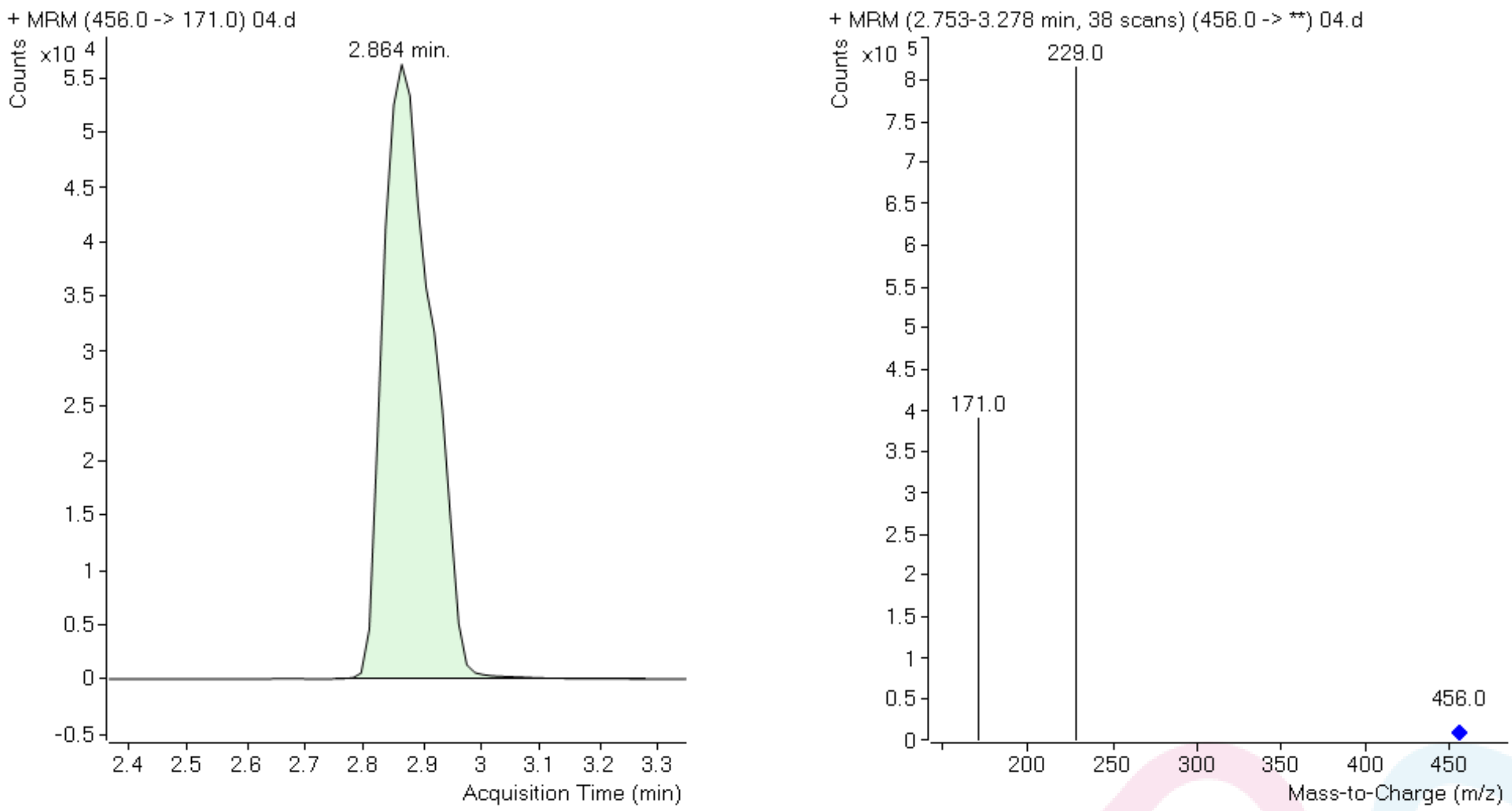

A calibration curve was then obtained for each peptide and the correlation index (R) is indicated.

The rest of the peptide standards were successfully detected by our protocol, demonstrating that the developed analytical method can detect these peptide samples.

Hyp-Gly

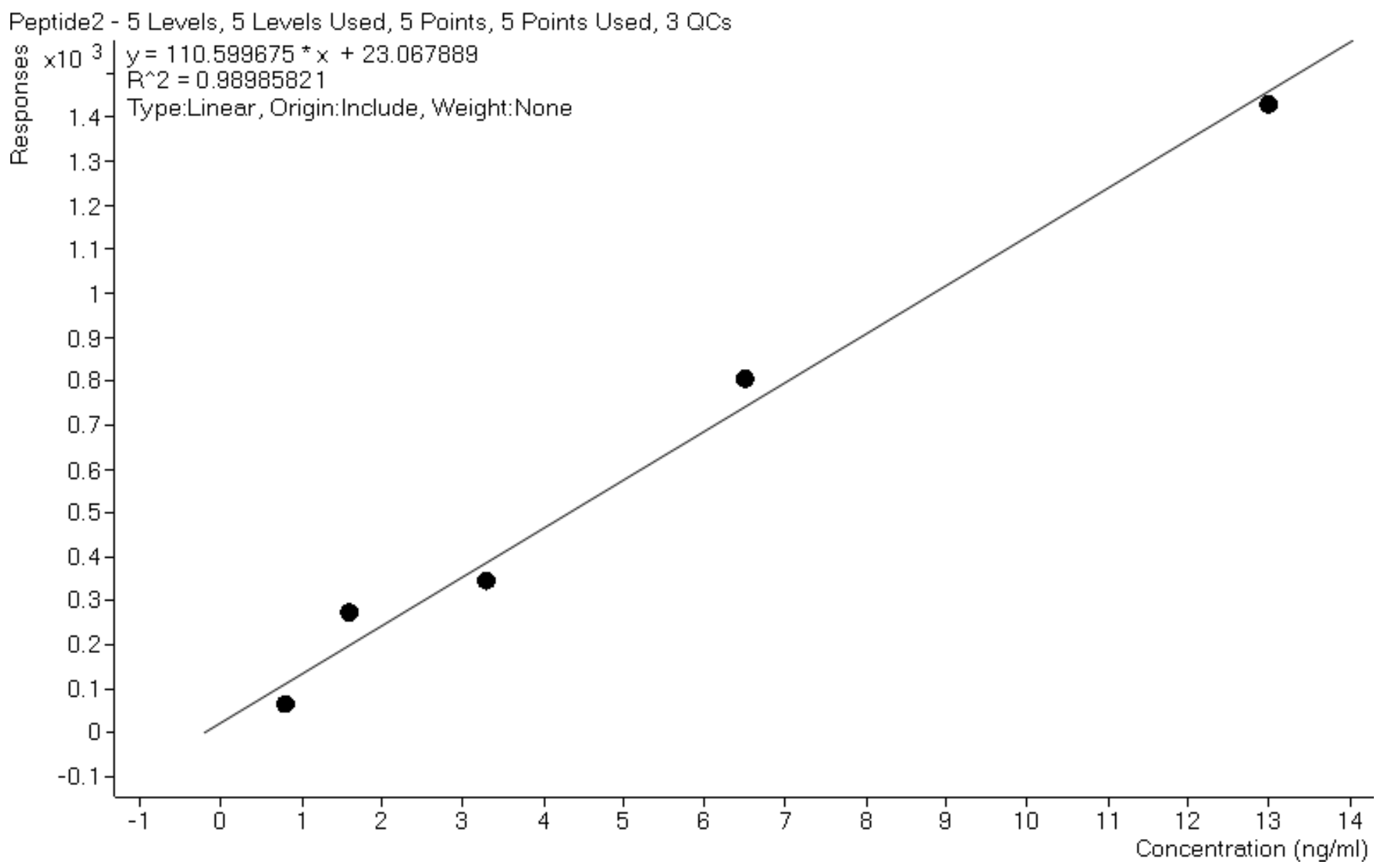

Ala-Hyp

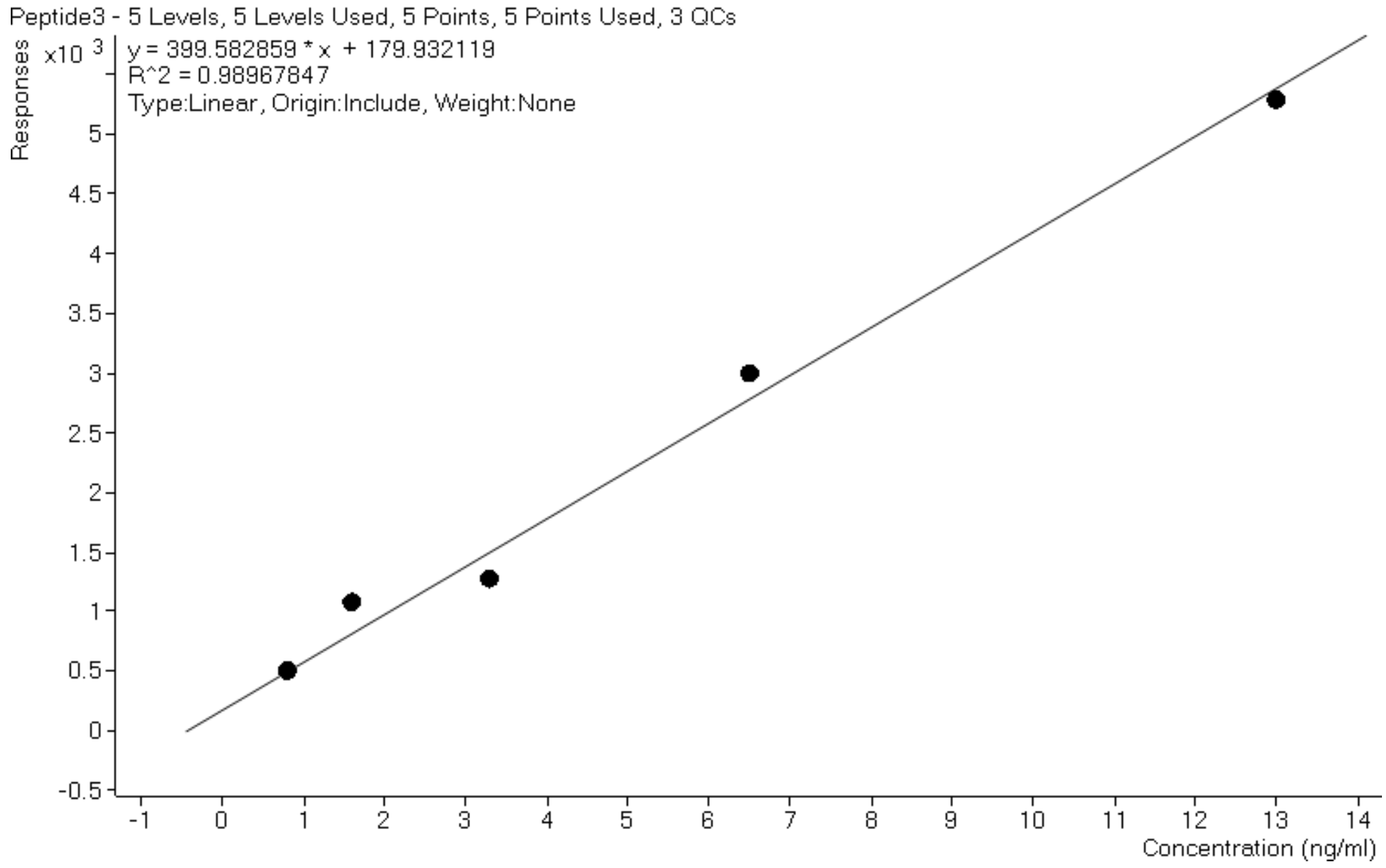

Gly-Pro

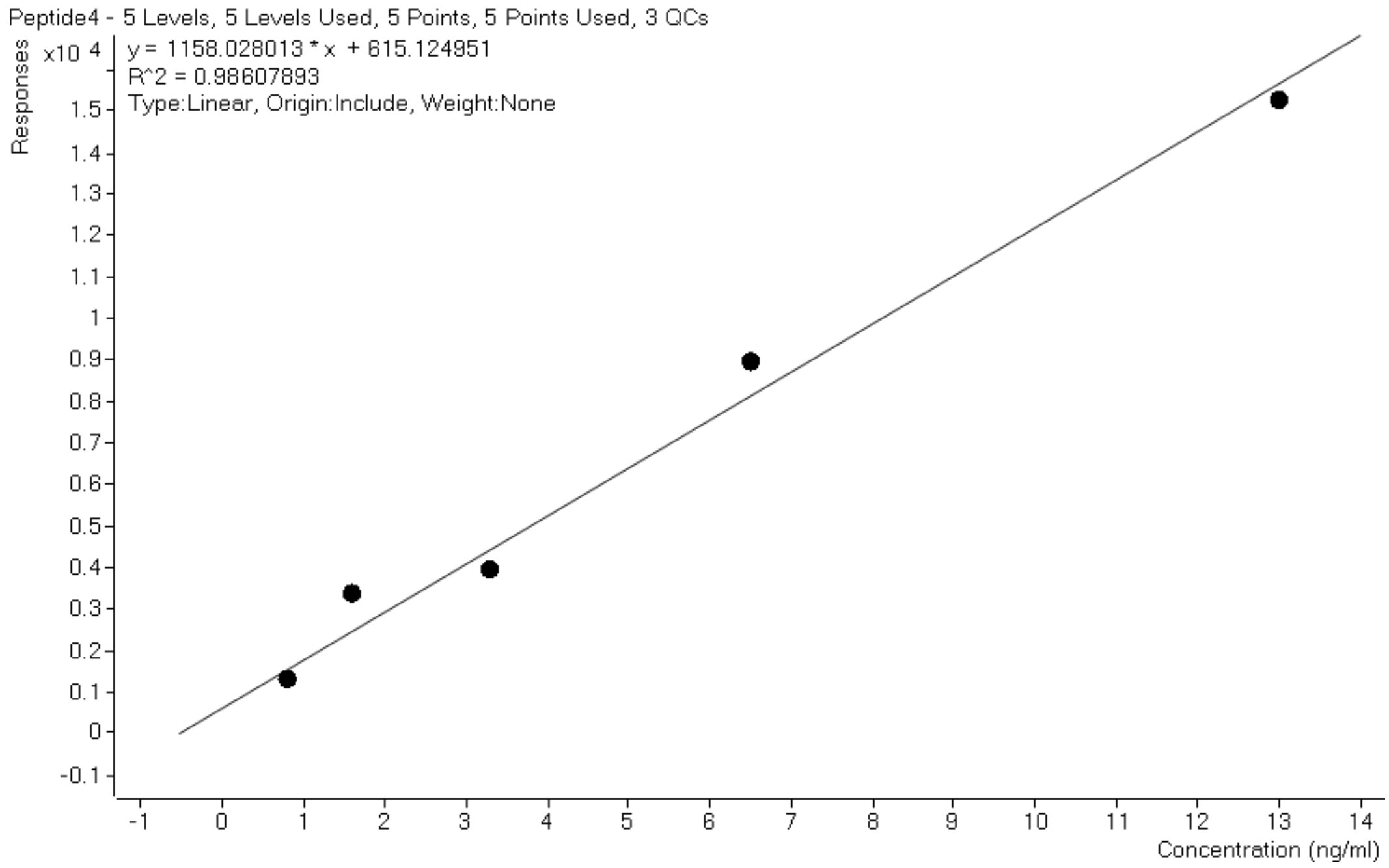

Gly-Pro-Hyp

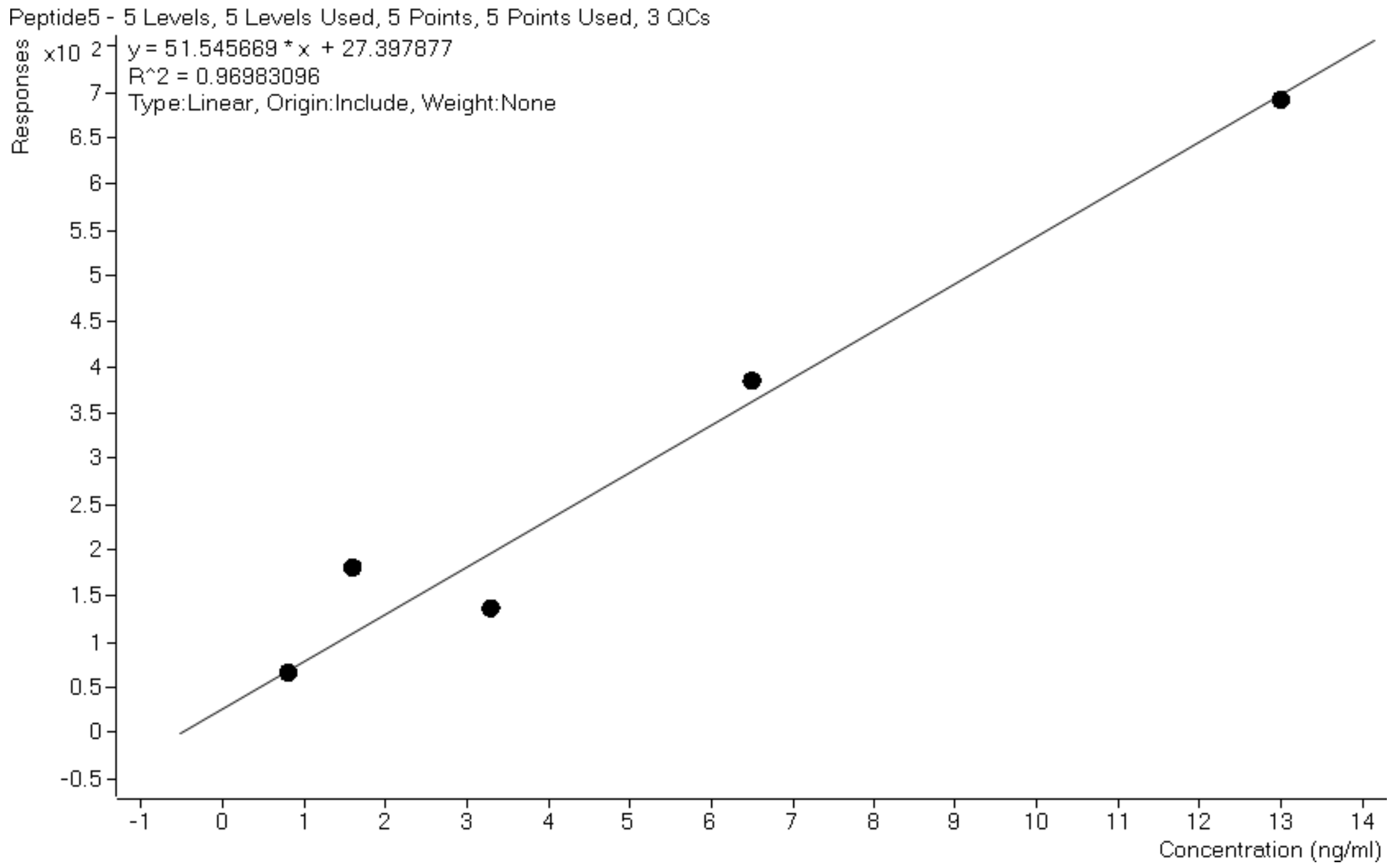

Ala-Hyp-Gly

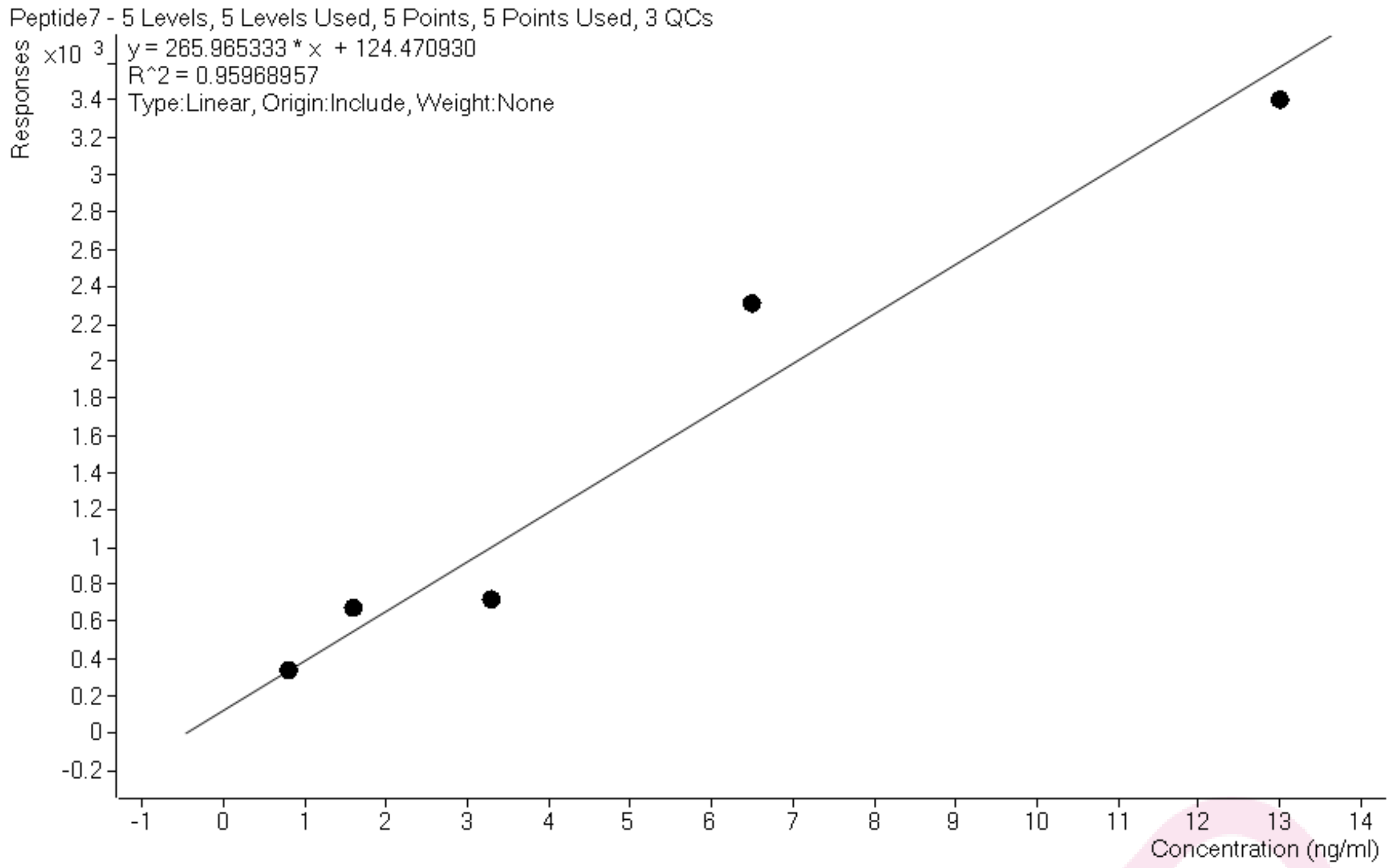

Ser-Hyp-Gly

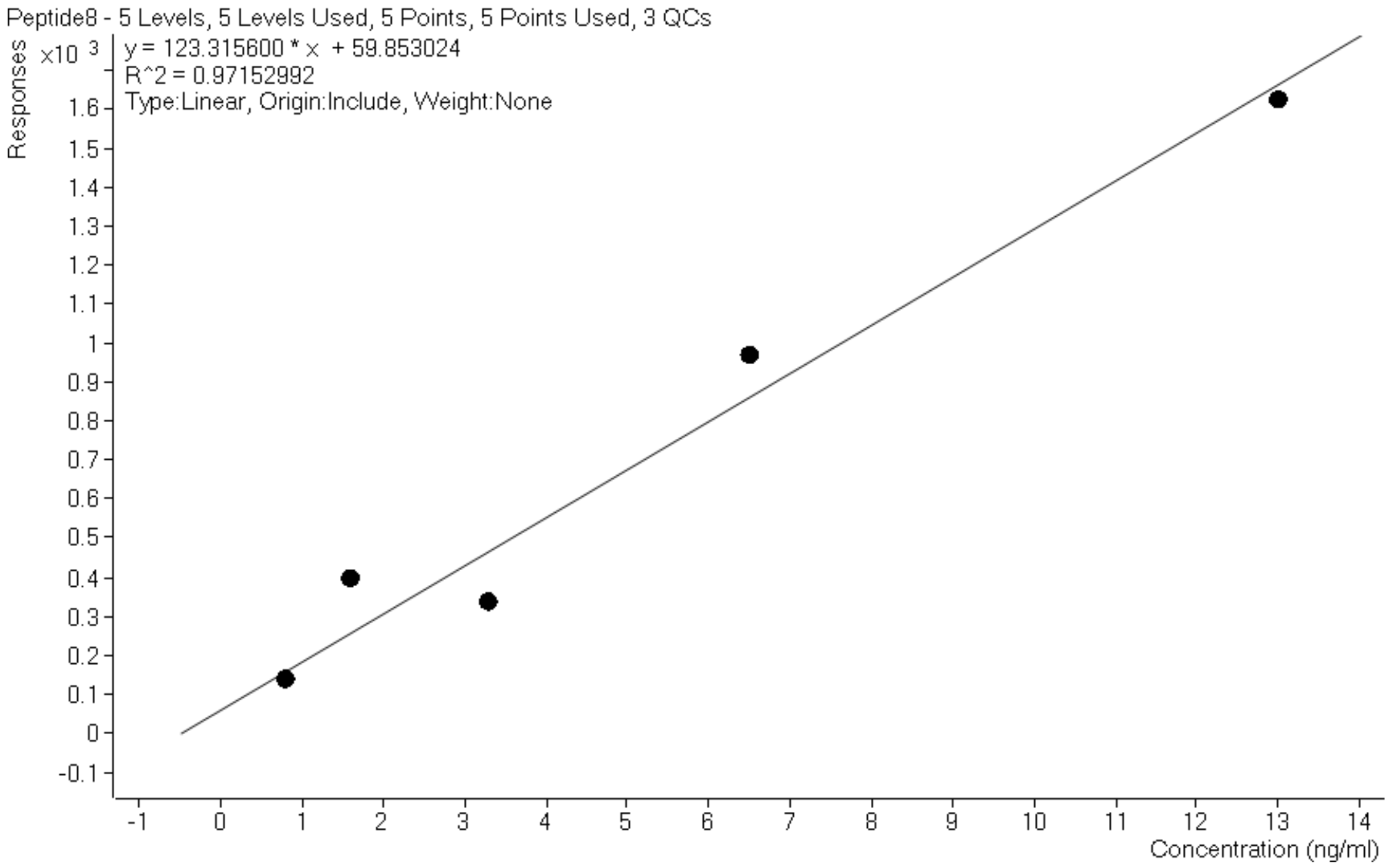
Peptide8 - 5 Levels, 5 Levels Used, 5 Points, 5 Points Used, 3 QCs
y = 123.315600 * x + 59.853024
R^2 = 0.97152992
Type:Linear, Origin:Include, Weight:None
Responses
x10 3
1.6
1.5
1.4
1.3
1.2
1.1
1
0.9
0.8
0.7
0.6
0.5
0.4
0.3
0.2
0.1
0
-0.1
-1
0
1
2
3
4
5
6
7
8
9
10
11
12
13
14
Concentration (ng/ml)

8 References

Al-Atif, H. (2022). Collagen Supplements for Aging and Wrinkles: A Paradigm Shift in the Fields of Dermatology and Cosmetics. Dermatology Practical & Conceptual, 12(1), e2022018. https://doi.org/10.5826/dpc.1201a18

Asai, T., Takahashi, A., Ito, K., Uetake, T., Matsumura, Y., Ikeda, K., Inagaki, N., Nakata, M., Imanishi, Y., & Sato, K. (2019). Amount of Collagen in the Meat Contained in Japanese Daily Dishes and the Collagen Peptide Content in Human Blood after Ingestion of Cooked Fish Meat. Journal of Agricultural and Food Chemistry, 67(10), 2831-2838. https://doi.org/10.1021/acs.jafc.8b06896

Avila Rodríguez, M. I., Rodríguez Barroso, L. G., & Sánchez, M. L. (2018). Collagen: A review on its sources and potential cosmetic applications. Journal of Cosmetic Dermatology, 17(1), 20-26. https://doi.org/10.1111/jocd.12450

Bröker, M. E. E., Lalmahomed, Z. S., Roest, H. P., van Huizen, N. A., Dekker, L. J. M., Calame, W., Verhoef, C., IJzermans, J. N. M., & Luider, T. M. (2013). Collagen Peptides in Urine: A New Promising Biomarker for the Detection of Colorectal Liver Metastases. PLoS ONE, 8(8), e70918. https://doi.org/10.1371/journal.pone.0070918

Gallegos-Tintoré, S., Torres-Fuentes, C., Solorza-Feria, J., Alaíz Barragán, M., Girón-Calle, J., Martínez-Ayala, A. L., Chel Guerrero, L., & Vioque, J. (2015). Antioxidant and chelating activity of nontoxic Jatropha curcas L. protein hydrolysates produced by in vitro digestion using pepsin and pancreatin. https://doi.org/10.1155/2015/190129

Gao, J., Ma, Y., Guo, Z., Zhang, Y., Xing, F., Zhang, T., Kong, Y., Luo, X., Xu, L., & Zhang, G. (2023). Evaluating the Degradation Process of Collagen Sponge and Acellular Matrix Implants In Vivo Using the Standardized HPLC-MS/MS Method. Separations, 10(1), Article 1. https://doi.org/10.3390/separations10010047

Guo, S., Xu, X., Zhou, X., & Huang, Y. (2018). A rapid and simple UPLC-MS/MS method using collagen marker peptides for identification of porcine gelatin. RSC Advances, 8(7), 3768-3773. https://doi.org/10.1039/C7RA12539A

Heidenreich, E., Pfeffer, T., Kracke, T., Mechtel, N., Nawroth, P., Hoffmann, G. F., Schmitt, C. P., Hell, R., Poschet, G., & Peters, V. (2021). A Novel UPLC-MS/MS Method Identifies Organ-Specific Dipeptide Profiles. International Journal of Molecular Sciences, 22(18), 9979. https://doi.org/10.3390/ijms22189979

Ichikawa, S., Morifuji, M., Ohara, H., Matsumoto, H., Takeuchi, Y., & Sato, K. (2010). Hydroxyproline-containing dipeptides and tripeptides quantified at high concentration in human blood after oral administration of gelatin hydrolysate. International Journal of Food Sciences and Nutrition, 61(1), 52-60. https://doi.org/10.3109/09637480903257711

Kumazawa, Y., Taga, Y., Iwai, K., & Koyama, Y. (2016). A Rapid and Simple LC-MS Method Using Collagen Marker Peptides for Identification of the Animal Source of Leather. Journal of Agricultural and Food Chemistry, 64(30), 6051-6057. https://doi.org/10.1021/acs.jafc.6b02132

Kumazawa, Y., Taga, Y., Takashima, M., & Hattori, S. (2018). A novel LC–MS method using collagen marker peptides for species identification of glue applicable to samples with multiple animal origins. Heritage Science, 6(1), 43. https://doi.org/10.1186/s40494-018-0209-y

Liu, D., Nikoo, M., Boran, G., Zhou, P., & Regenstein, J. M. (2015). Collagen and gelatin. Annual Review of Food Science and Technology, 6, 527-557. https://doi.org/10.1146/annurev-food-031414-111800

Pataridis, S., Eckhardt, A., Mikulíková, K., Sedláková, P., & Miksík, I. (2008). Identification of collagen types in tissues using HPLC-MS/MS. Journal of Separation Science, 31(20), 3483-3488. https://doi.org/10.1002/jssc.200800351

Quirós, A., del Mar Contreras, M., Ramos, M., Amigo, L., & Recio, I. (2009). Stability to gastrointestinal enzymes and structure-activity relationship of beta-casein-peptides with antihypertensive properties. Peptides, 30(10), 1848-1853. https://doi.org/10.1016/j.peptides.2009.06.031

Rohm, F., Daniel, H., & Spanier, B. (2019). Transport Versus Hydrolysis: Reassessing Intestinal Assimilation of Di- and Tripeptides by LC-MS/MS Analysis. Molecular Nutrition & Food Research, 63(21), e1900263. https://doi.org/10.1002/mnfr.201900263

Song, H., Tian, Q., & Li, B. (2020). Novel Hyp-Gly-containing antiplatelet peptides from collagen hydrolysate after simulated gastrointestinal digestion and intestinal absorption. Food & Function, 11(6), 5553-5564. https://doi.org/10.1039/D0FO00219D

Sugihara, F., Inoue, N., Kuwamori, M., & Taniguchi, M. (2012). Quantification of hydroxyprolyl-glycine (Hyp-Gly) in human blood after ingestion of collagen hydrolysate. Journal of Bioscience and Bioengineering, 113(2), 202-203. https://doi.org/10.1016/j.jbiosc.2011.09.024

Taga, Y., Kusubata, M., Ogawa-Goto, K., & Hattori, S. (2017). Identification of Collagen-Derived Hydroxyproline (Hyp)-Containing Cyclic Dipeptides with High Oral Bioavailability: Efficient Formation of Cyclo(X-Hyp) from X-Hyp-Gly-Type Tripeptides by Heating. Journal of Agricultural and Food Chemistry, 65(43), 9514-9521. https://doi.org/10.1021/acs.jafc.7b03714

Xu, J., Xing, F., Luo, X., Gao, J., Zhang, Y., Zhang, G., Bai, X., & Huang, C.-C. (2022). Quantitation of Collagen Type V in Tissues by High-Performance Liquid Chromatography Coupled to Mass Spectrometry. Tissue Engineering Part C: Methods, 28(3), 95-103. https://doi.org/10.1089/ten.tec.2022.0003

Yamamoto, S., Deguchi, K., Onuma, M., Numata, N., & Sakai, Y. (2016). Absorption and Urinary Excretion of Peptides after Collagen Tripeptide Ingestion in Humans. Biological & Pharmaceutical Bulletin, 39(3), 428-434. https://doi.org/10.1248/bpb.b15-00624

Yazaki, M., Ito, Y., Yamada, M., Goulas, S., Teramoto, S., Nakaya, M., Ohno, S., & Yamaguchi, K. (2017). Oral Ingestion of Collagen Hydrolysate Leads to the Transportation of Highly Concentrated Gly-Pro-Hyp and Its Hydrolyzed Form of Pro-Hyp into the Bloodstream and Skin. Journal of Agricultural and Food Chemistry, 65(11), 2315-2322. https://doi.org/10.1021/acs.jafc.6b05679

9 Registry and Regulation

The final report, raw data and the assay protocol have been saved in a digital format and on a paper copy. All the information provided from the client, and generated by Bionos Biotech will be considered as confidential. Information regarding materials, reagents and protocols adopted by Bionos Biotech SL during the assays is confidential and will not be shared with third parts.

The whole process involving this assay was performed under Quality Management System UNE-EN-ISO 9001/2015.

Article 20 of the EC Cosmetic Products Regulation 1223/2009 (CPR) frames the requirements for cosmetic claims. Furthermore, cosmetic claims have to comply with EU Regulation 655/2013 that provides the Common Criteria to ensure that the information conveyed to the end-users through claims is useful, understandable, and reliable so that consumers can make informed decisions.

The third Common Criterion, 'Evidential support', states that "claims for cosmetic products, whether explicit or implicit, shall be supported by adequate and verifiable evidence regardless of the types of evidential support used to substantiate them, including where appropriate expert assessments. Evidence for claim substantiation shall take into account the state-of-the-art practices. Where studies are being used as evidence, they shall be relevant to the product and the benefit claimed, shall follow well-designed, well-conducted methodologies (valid, reliable, and reproducible) and shall respect ethical considerations."

Experimental studies include (but are not limited to) studies in silico, in vitro, ex-vivo, with instrumental or biochemical methods, studies conducted on volunteers, investigator evaluations, sensory evaluations, etc. Different types of experimental studies can be used to provide data on the performance of cosmetic products. It is useful to take into consideration existing relevant guidelines, e.g., guidelines relating to instrumental clinical techniques, other European or international guidelines or standards (e.g., CEN, ISO, etc.).

Such studies should comprise methods which are reliable and reproducible. The studies should follow a well-designed and scientifically valid methodology according to best practices. The criteria used for evaluation of product performance should be defined with accuracy and chosen in accordance with the aim of the test. The experimental aspect of studies calls for reliance on knowledge and awareness of statistical principles in the design and analysis of the study, e.g., in terms of number of subjects, test samples, etc. This is necessary in order to ensure that the studies achieve scientifically and statistically valid conclusions.

A study protocol should be drawn up and validated in order to enable the study to be conducted and monitored appropriately, thereby ensuring its quality. Whatever the type of study, it is important that the person conducting the study has the appropriate qualifications, has training and experience in the field of the proposed study and has high ethical qualitied standards and professional integrity.

Test facilities should maintain a quality assurance system, including standardised operating procedures. A monitoring system should be set up for each study in order to ensure that the protocol and the operating procedures are correctly followed.

Data processing and the interpretation of results should be fair and should not overstep the limits of the test's significance. Data recording, transformations, and representation in tabular or graphical form should be transparent or clearly explained if complex. It should not be designed to overstate the effect(s) measured. Appropriate statistical analysis of the data should be performed.
