# Supplementary material for: Enhanced digestion and absorption kinetics of ultra–low molecular weight collagen containing di- and tripeptides: evidence from human and cell models
Source: PeerJ. 2026 Aug 14;14:e21398. doi: 10.7717/peerj.21398 (PMC13480140; doi:10.7717/peerj.21398)
Supplement: Supplementary file 3 [file peerj-14-21398-s005.pdf]

Bionos
Testing Efficacy

RESEARCH PROJECT – Collagen_2022

Protocol Code of Study: Collagen_2022

Title: Determination of bioavailability and identification of amino acid profile, di- and tri-peptides in blood after oral ingestion of collagen peptides: Randomized, blinded and placebo controlled clinical study.

Products:

- Four different types of hydrolyzed type I collagen/collagen peptides (CP1, CP2, CP3, CP4).
- One placebo.

Sponsor: PBLeiner, Part of Tessenderlo Group

Version 2, Date November 22nd, 2022

CONFIDENTIAL

This protocol is owned by the sponsor and cannot be disclosed without written authorization

RESPONSABILITIES AND SIGNATURES

By signing this protocol entitled "***Determination of bioavailability and identification of amino acid profile, di- and tri-peptides in blood after oral ingestion of collagen peptides: Randomized, blinded and placebo controlled clinical study***", the below signatories agree to carry out the **Collagen_2022** study in accordance with the following documents:

- Protocol of the study "Version 2, Date November 22nd, 2022".
- Declaration of Helsinki, Brazil 2013.
- Current regulation related to research projects.

Sponsor

PBLeiner, Part of Tessenderlo Group

Dr. Reyhan Nergiz Unal, PhD<br>(Health & Nutrition Science Lead)	22/11/2022

Principal Researcher

BIONOS BIOTECH S.L.

Dr. José Luis Mullor Sanjosé (Chief Scientific Officer and CEO)	22/11/2022

SUMMARY

Protocol Title: *Determination of bioavailability and identification of amino acid profile, di- and tri-peptides in blood after oral ingestion of collagen peptides: Randomized, blinded and placebo controlled clinical study.*

Principal Researcher: Dr. José Luis Mullor Sanjosé

Researchers: Dra. Adela Serrano

Research Centers: Bionos Biotech S.L.

Duration of Study: 4 hours

<u>Hypothesis</u>

Collagen is characterized by its high content of glycine, proline and hydroxyproline, and it is found to exert beneficial effects on several health benefits. Recent evidence suggests that the consumption of amino acids and/or peptides abundantly present in collagen may have the capacity to influence the synthesis of new collagen in the body [1]. Indeed, it has been found that collagen supplements improve skin moisture, elasticity, and hydration when orally administered [1]. Additionally, collagen reduces the wrinkling and roughness of the skin, and existing studies have not found any side effects of its oral supplements [1]. Thus, to exert any beneficial effects, it is essential that hydrolyzed collagen is optimally absorbed. This study aimed to investigate the postprandial absorption of collagen and elucidate the absorption rate and bioavailability after oral intake.

<u>Dosage</u>

- The study personnel will prepare the drinkable solutions containing the study products in a room accessible to site staff only. Drinkable solutions will be provided to subjects just before consumption.
- Product ingestion will be early in the morning after overnight fasting for 12 h. On the morning of the experiment, subjects will fast and each subject will take the products orally.
- Dosage (Quantity of product to consume orally): This is calculated depending on the body weight; 400 mg/kg of body weight with 20 w/v % water. Collagen products (fine powder) will be diluted in water prior consumption. Control group ingest approximately same amount of plain water. Participants cannot drink water within 4 hours before and after the product consumption.

<u>Safety</u>

All documentation related to product safety is presented in the files attached to this protocol.

The manufactured products by PBLeiner tested in this project are produced in compliance with:

- Regulation (EC) No 178/2002 of the European Parliament and of the council laying down the general principles and requirements of food law, establishing the European

Food Safety Authority and laying down procedures in matters of food safety.

- Regulation (EC) No 853/2004 of the European Parliament and of the Council laying down specific hygiene rules for food of animal origin and amendments to this regulation.
- Commission Regulation (EC) No 2073/2005 on microbiological criteria for foodstuffs.

Methodology

The assessment of the bioavailability of the four collagen-based treatments (CP1, CP2, CP3, CP4) will be evaluated by quantifying 8 di/tri-peptides and the amino acid profile in the blood of 25 volunteers.

The study personnel will prepare the drinkable solutions containing the study products in a room accessible to site staff only. Drinkable solutions will be provided to subjects just before consumption

Upon oral intake, blood samples will be obtained by digital puncture at each time point; T0 (before consuming the product), and 15 min., 30 min., 120 min. and 240 min. after consuming the product. The digital puncture will be performed with Verifine Safety Lancets, and the blood will be collected in MiniCollect tubes. Blood samples will be centrifuged (1000 rpm, 5 minutes, 4ºC) and plasma will be collected. The profile of single aminoacids and selected di- and tripeptides will be determined in the plasma samples by mass spectrometry (LC-MS). The following molecules will be analyzed:

- Single aminoacids (approx. 20 aminoacids, but especially hydroxyproline, glycine, proline)
- Dipeptides (Pro-Hyp, Hyp-Gly, Ala-Hyp, Phe-Hyp)
- Tripeptides (Gly-Pro-Hyp, Pro-Hyp-Gly, Ala-Hyp-Gly, Ser-Hyp-Gly)

All the process will be developed in the headquarters of Bionos Biotech, S.L.

Number of Subjects: 25 volunteers organized in 5 groups of 5 volunteers. Each group will participate in the evaluation of each product (including the placebo, which will be considered the control group).

Diagnosis and main criteria of inclusion and exclusion: Healthy volunteers.

Inclusion Criteria

- Healthy male volunteers to avoid any possible hormonal interference.
- Between 25-45 years old (both included).
- Body weight is 70 kg (60-80 kg (both included), the range depends on the body mass index (BMI).
    - BMI in normal (healthy weight and overweight) range: 18.5 to 29.9 kg/m2. (Reference: WHO and CDC).
- Co-operative and available during the study period.
- Subjects with capacity to understand the purpose and protocol of the study and sign the Informed Consent.

Exclusion Criteria

- Diagnosis of allergies to gelatin, red meat (Alpha Gal Syndrome) or fish.
- Diagnosis of diseases about collagen metabolism such as Goodpasture syndrome, scleroderma, periarteritis nodosa or polyarteritis, dermatomyositis, and disseminated lupus erythematosus.
- Diagnosis of following chronic Gastro-Intestinal Tract diseases blocking transfer of polypeptides from GIS to the blood such as: Peptic Ulcer, Duodenal Ulcer, Chronic Atrophic Gastritis, *Helicobacter Pylori*, Chronic Peptic Disorders, Chronic Acid Reflux = GER and GERD, Celiac Disease, Crohn's Disease, Irritable Bowel Syndrome, Lactose Intolerance, Diverticulitis, Diverticulosis.
- Diagnosis of any diseases such as cardiovascular disease, chronic kidney disease (CKD), gastrointestinal disorder, endocrinological disorder, immunological disorder, metabolic disease.
- Consume more than the recommended alcohol guidelines i.e. >21 alcohol units/week for males and >14 units/week for females (europa.eu).
- Current smoking habit or history of smoking within the past 1 year.
- History of depression, schizophrenia, alcoholism, drug addiction, or mental illness.
- Current or previous intake of contraceptives, female hormones, obesity drugs, absorption inhibitors, antidepressants, or appetite suppressants.
- History of asthma or autoimmune disease Use of oral hormone therapies ie. cortisone or steroids in the 6 months prior to initiation of the study.
- Abnormal liver function or abnormal renal function.
- Blood pressure >140/90 mmHg or hypertension with intake of a diuretic.
- No participation to another clinical trial in the last 6 months.
- No blood donation within a month.
- Problems with overall findings in blood-test results as determined by a specialist.
- Any condition judged by the investigator to be unsuitable for participation in

the study.
**Products under investigation:**<br>• Four different types of hydrolyzed type I collagen/collagen peptides (CP1, CP2, CP3, CP4).<br>• One placebo.<br>There is no health risk for the oral intake of these products by volunteers, as they have passed all relevant safety and toxicology studies, as described above.<br>**Route of administration:** Oral intake.<br>Product ingestion will be early in the morning after overnight fasting for 12 h. On the morning of the experiment, subjects will fast and each subject will take the products orally.<br>**Dose:** It's calculated depending on the body weight; 400 mg/kg of body weight with 20 w/v % water. Collagen products (fine powder) will be diluted in water prior consumption. Control group (placebo) ingest approximately same amount of plain water. Participants cannot drink water within 4 hours before and after the product consumption.
**Duration of treatment:** 4 hours.

Table A: Schedule of study / procedures for one subject

Time 0 hours (before oral intake)	Oral intake of the product	Time 15 min., 30 min., 120 min. and 240 min. (after oral intake)
Digital puncture and blood sample collection.	-	Digital puncture and blood sample collection.
-	-	Obtain plasma from blood samples. Quantification of single aminoacids and di/tripeptides in plasma samples by LC-MS.

1. INTRODUCTION

1.1. Background

Collagen is a fibrous protein highly demanded in biomedical and cosmetic industries due to its benefits on the skin, biocompatibility, bioactivity, and weak immunogenicity [8]. This biomaterial is composed of repetitive triplets of amino acids glycine (Gly), proline (Pro), and hydroxyproline (Hyp), and it is found to exert beneficial effects on several health benefits. Proteins in their native form such as collagen present low solubility in water due to its high molecular weight of approximately 300 kDa. These natural properties present difficulties in their application to cosmetics and edible products. However, the hydrolyzed collagen (HC), which consists of segmented proteins with low molecular weight (Mw) between 1 kDa and 10 kDa, do not have this problem. The use of natural peptides like collagen hydrolysates has been widely utilized due to their excellent biocompatibility, easy biodegradability, and weak antigenicity [9]. Recent evidence suggests that the consumption of aminoacids and/or peptides abundantly present in collagen may have the capacity to influence the synthesis of new collagen in the body [1]. Indeed, it has been found that collagen supplements improve skin moisture, elasticity, and hydration when orally administered [1]. Additionally, collagen reduces the wrinkling and roughness of the skin, and existing studies have not found any side effects of its oral supplements [1]. Thus, to exert any beneficial effects, it is essential that hydrolyzed collagen is optimally absorbed.

In the present study, we aim to study the postprandial absorption of different types of hydrolyzed collagen molecules and elucidate the impact of raw material and molecular weight (less than 5000 Da) on absorption rate and bioavailability after oral intake.

The product ingestion will be early in the morning after overnight fasting for 12 h. On the morning of the experiment, subjects will fast and each subject will take the products orally. The dose is calculated depending on the body weight; 400 mg/kg of body weight with 20 w/v % water. Collagen products (fine powder) will be diluted in water prior consumption. Control group ingest approximately same amount of plain water. Participants cannot drink water within 4 hours before and after the product consumption.

The manufactured products by PB Leiner tested in this project are produced in compliance with:

- Regulation (EC) No 178/2002 of the European Parliament and of the council laying down the general principles and requirements of food law, establishing the European Food Safety Authority and laying down procedures in matters of food safety.
- Regulation (EC) No 853/2004 of the European Parliament and of the Council laying down specific hygiene rules for food of animal origin and amendments to this regulation.
- Commission Regulation (EC) No 2073/2005 on microbiological criteria for foodstuffs.

1.2. Justification of the trial

Taking together the above information, we hypothesize that oral intake of hydrolyzed collagen products leads to an increase of collagen-derived aminoacid and peptides in the blood of volunteers, compared to the placebo product. We will assess the absorption of the collagen-derived products by quantifying specific aminoacids, dipeptides and tripeptides by LC-MS in the blood obtained by digital puncture.

The benefits of the study lie in the possible health improvement that different products may have in the body, as well as in the market launch of a new product, with effectiveness in increasing the collagen-associated skin health.

Likewise, volunteers will be compensated with the amount of TWENTY €, which will be given in a single payment taking place during the final study visit (20 €) if the study obligations have been fulfilled.

The research team will not receive additional economic benefit.

Products:

- Four different types of hydrolyzed type I collagen/collagen peptides (CP1, CP2, CP3, CP4).
- One placebo.

The study will be carried out according to the presented protocol and the corresponding legal requirements. The population of the study will be 25 healthy volunteers, according to the inclusion and exclusion criteria, explained below in the present protocol.

2. OBJECTIVES OF THE STUDY

Clinical evaluation of the bioavailability of four collagen-based treatments and placebo after oral intake by quantifying eight di/tri-peptides and amino acid profile in the blood of 25 volunteers.

3. DESIGN OF THE STUDY

Treatment:

The assessment of the bioavailability of the four collagen-based treatments (CP1, CP2, CP3, CP4) will be evaluated by quantifying 8 di/tri-peptides and amino acid profile in the blood of 25 volunteers.

Then, the day when the study starts, the volunteers will be required to fast overnight during 12 hours. Blood samples will be obtained early in the morning by digital puncture before consuming the product, and this time will be the time 0 (T0). Right after, the volunteers will take the product by oral intake according to the established dosage (400 mg/kg body weight). From this point on, blood samples will be taken by digital puncture at 15 min., 30 min., 120 min. and 240 min. after the oral intake. The digital puncture will be performed with Verifine Safety Lancets, and the blood will be collected in MiniCollect tubes. Blood samples will be centrifuged (1000 rpm, 5 minutes, 4ºC) and plasma will be collected. The profile of single aminoacids and selected di- and tripeptides will be determined in the plasma samples by mass spectrometry (LC-MS). The following molecules will be analyzed:

- Single aminoacids (approx. 20 aminoacids, but especially hydroxyproline, glycine, proline)
- Dipeptides (Pro-Hyp, Hyp-Gly, Ala-Hyp, Phe-Hyp)
- Tripeptides (Gly-Pro-Hyp, Pro-Hyp-Gly, Ala-Hyp-Gly, Ser-Hyp-Gly)

All the process will be developed in the headquarters of Bionos Biotech, S.L.

Duration: 4 hours.

**Time 0 hours (before oral intake)**	**Oral intake of the product**	**Time 15 min., 30 min., 120 min. and 240 min. (after oral intake)**
Digital puncture and blood sample collection.	-	Digital puncture and blood sample collection.
-	-	Obtain plasma from blood samples. Quantification of single aminoacids and di/tripeptides in plasma samples by LC-MS.

4. Panel selection

The person in charge of selecting the 25 volunteers will be the Principal Investigator of the study, Dr. José Luis Mullor Sanjosé (PhD in Biochemistry and Molecular Biology), in collaboration with the Responsible Investigator Dr. Adela Serrano Gimeno and the promoter PBLeiner.

The participants of the study will be recruited in the Bionos Biotech facilities on a voluntary basis after the promotion and publicity of the study by the social networks of Bionos Biotech. Participants will accept the study conditions after signing the patient information sheet.

Volunteers will be considered eligible if they meet the inclusion and exclusion criteria.

Some extra volunteers will be included to account for the unforeseen withdrawals of the volunteers during the study. Extra volunteers will be divided according to the needs and circumstances of the moment.

4.1. Inclusion Criteria

- Healthy male volunteers to avoid any possible hormonal interference.
- Between 25-45 years old (both included).
- Body weight is 70 kg (60-80 kg (both included), the range depends on the body mass index (BMI).
    - BMI in normal (healthy weight and overweight) range: 18.5 to 29.9 kg/m2. (Reference: WHO and CDC).
- Informed of the purpose and the protocol of the study and signed a written informed-consent form.
- Co-operative and available during the study period.

4.2. Exclusion Criteria

- Diagnosis of allergies to gelatin, red meat (Alpha Gal Syndrome) or fish.
- Diagnosis of diseases about collagen metabolism such as Goodpasture syndrome, scleroderma, periarteritis nodosa or polyarteritis, dermatomyositis, and disseminated lupus erythematosus.
- Diagnosis of following chronic Gastro-Intestinal Tract diseases blocking transfer of polypeptides from GIS to the blood such as: Peptic Ulcer, Duodenal Ulcer, Chronic Atrophic Gastritis, *Helicobacter Pylori*, Chronic Peptic Disorders, Chronic Acid Reflux = GER and GERD, Celiac Disease, Crohn's Disease, Irritable Bowel Syndrome, Lactose Intolerance, Diverticulitis, Diverticulosis.
- Diagnosis of any diseases such as cardiovascular disease, chronic kidney disease (CKD), gastrointestinal disorder, endocrinological disorder, immunological disorder, metabolic disease.
- Consume more than the recommended alcohol guidelines i.e. >21 alcohol units/week for males and >14 units/week for females (europa.eu).
- Current smoking habit or history of smoking within the past 1 year.
- History of depression, schizophrenia, alcoholism, drug addiction, or mental illness.

- Current or previous intake of contraceptives, female hormones, obesity drugs, absorption inhibitors, antidepressants, or appetite suppressants.
- History of asthma or autoimmune disease Use of oral hormone therapies ie. cortisone or steroids in the 6 months prior to initiation of the study.
- Abnormal liver function or abnormal renal function.
- Blood pressure >140/90 mmHg or hypertension with intake of a diuretic.
- No participation to another clinical trial in the last 6 months.
- No blood donation within a month.
- Problems with overall findings in blood-test results as determined by a specialist.
- Any condition judged by the investigator to be unsuitable for participation in the study.

4.3. Criteria for Withdrawal of subjects

- If the subject/s shows any type of gastrointestinal or allergic reaction after intake of the product, the corresponding subject will be removed.
- If the subject is removed, the measurements after the removal will not be taken and its results will not be taken into consideration for the analysis. The adverse reaction will be registered in the final report.
- If the subject is removed from the study, follow-up on the same will be maintained to confirm the disappearance of the reaction, after the passage of days.
- If the subject is removed or withdraws from the study, it will be replaced with a new subject, who will begin the study from the beginning.

The subjects may withdraw its consent for the participation at any time without any prejudice. In case of withdrawal, the researcher will put all the means to determine if the abandonment is due to causes related to the research product. Additionally, the researcher may withdraw a subject if, according to the clinical opinion of the researcher,it is in the best interest of the subject or if the subject cannot comply with therequirements of the protocol.

The sponsor can interrupt the protocol at any time. The reasons for stopping the studymay include, but are not limited to, the following:

- Unacceptable incidence of adverse events.
- Inadequate inclusion of subjects.
- Inadequate or inaccurate data collection.

4.4. Study Obligations

- To follow the protocol of the study.
- To keep the personal eating habits.
- Do not take drugs or dietary supplements, aimed at reducing weight or regulating gastrointestinal metabolic parameters during the study period.

4.5. Approximate duration of subject's recruitment

The period of subject's recruitment is estimated in 2-4 weeks from the beginning of the

study. The follow-up period for the subjects is 1 day. The 25 volunteers will be recruited by Bionos Biotech S.L., from its own database of volunteers. Different round of recruitment will be carried out, by means of email communication, where basic information about the study will be provided, until de adequate number of volunteers are recruited. Once the volunteers are confirmed, they will be asked to meet in Bionos Biotech's headquarters to be informed about the studyand the procedure to be followed

5. TREATMENT OF THE VOLUNTEER

The product ingestion will be early in the morning after overnight fasting for 12 h. On the morning of the experiment, subjects will fast and each subject will take the products orally. The dose is calculated depending on the body weight; 400 mg/kg of body weight with 20 w/v % water. Collagen products (fine powder) will be diluted in water prior consumption. Control group ingest approximately same amount of plain water. Participants cannot drink water within 4 hours before and after the product consumption.

The manufactured products by PB Leiner tested in this project are produced in compliance with:

- Regulation (EC) No 178/2002 of the European Parliament and of the council laying down the general principles and requirements of food law, establishing the European Food Safety Authority and laying down procedures in matters of food safety.
- Regulation (EC) No 853/2004 of the European Parliament and of the Council laying down specific hygiene rules for food of animal origin and amendments to this regulation.
- Commission Regulation (EC) No 2073/2005 on microbiological criteria for foodstuffs.

6. PROCEDURES OF STUDY

6.1. Selection / Initial Level

Recruitment of the 25 volunteers based on the inclusion and exclusion criteria.

6.2. Period of the study

Once finished the recruitment, the selected volunteers will be informed about the protocol of the study (by email).

Once they accept to participate in the study, based on the sent protocol, subject will be asked to meet at Bionos Biotech's headquarters, to be informed about the study and the procedure to be followed. In that meeting, the informed consent will be signed and guidelines in paper format will be explained. At the end of the meeting, volunteers will be called for measurements in Bionos Biotech, S.L. laboratory.

At every time, the Responsible Researcher Dra. Adela Serrano will keep a personalized follow-up in each of the volunteers, being frequently in contact with volunteers.

In the last measurement, if study obligations were fulfilled, according to the principal Researcher, the financial compensation will be paid.

7. EVALUATIONS

The bioavailability of the different pure collagen peptide products after oral intake will be assessed by LC-MS in the laboratory of Bionos Biotech, S.L., detailed as follows:

Bionos Biotech S.L.
Biopolo La Fe - Hospital La Fe, Torre A, 1ª
PlantaAv. Fernando Abril Martorell, 106
46026 Valencia (España)

TIMETABLE: 08:30 – 17:30

Data will be processed and analyzed in the headquarters of Bionos Biotech, S.L.

The procedure will take place according to the following schedule:

Time 0 hours (before oral intake)	Oral intake of the product	Time 15 min., 30 min., 120 min. and 240 min. (after oral intake)
Digital puncture and blood sample collection.	-	Digital puncture and blood sample collection.
-	-	Obtain plasma from blood samples. Quantification of single aminoacids and di/tripeptides in plasma samples by LC-MS.

8. ADVERSE EVENTS NOTIFICATION

8.1.1. Adverse events notification

Researchers are reminded of their obligation to notify the adverse reactions of which they have knowledge to the Pharmacosurveillance Center of their Autonomous Community, through the yellow card system, according to the current regulation of Real Decreto 1344/2007.

The classification of the events will be done according to the Research Projects regulations.

The events will be communicated to the principal researcher (Dr. Adela Serrano) immediately after having knowledge of it. Contact information:

Department: R&D
Phone: +34 96 124 32 19
Mobile Phone: +34 616 09 41 44
Email: aserrano@bionos.es

In case of SUE (Serious Undesirable Events, as defined in the regulation), the notification to the sponsor must be made within 24 hours from the knowledge of the event.

8.1.2. Follow-up of adverse events

If some subject is removed from the study, a follow-up will be kept on him to confirm the disappearance of the mentioned effects, after the passage of days.

9. STATISTICAL METHODS AND DETERMINATION OF SAMPLE SIZE

From the obtained data before and after the development of the study, a comparison ofresults will be performed, and the effects provoked by the intake of the different products will be determined.

The obtained results will be statistically analyzed, through the application of the unpaired Student's t-test. To consider significance, a confidence interval of 95 % will be applied, with p value < 0.05. 25 volunteers will be included in the study. The number of volunteers necessary to obtain a 5% difference in the mean between the two groups, with an alpha of 0.05 and a power of 0.8. The extensionof the study with a larger number of volunteers in the future is not ruled out. All subjectswill be included in the statistical analysis.

It is considered that the sample size is enough based on the power analysis for the purpose of the study, according to the results obtained in previous studies.

10. ETHICAL INFORMATION

10.1. Ethical performance of the study

This study will be conducted in accordance with the Declaration of Helsinki on ethical principles for medical research in human subjects, adopted by the General Assembly of the World Medical Association (1996). In addition, the study will be conducted in accordance with the protocol, in accordance with the guidelines of the international conference on harmonization (ICH) and the regulatory requirements for participating institutions. The study will be carried out according to the protocol reviewed by the Ethics Committee in Clinical Research (CEIC) and the Autonomic Committee of the Ministry of Health of the Generalitat Valenciana; the study will be carried out by scientifically and medically qualified people; the benefits of the study are in proportion to the risks; the rights and welfare of the subjects will be respected; the doctors who perform the study do not find that the risks outweigh the possible benefits and each subject will provide their informed consent without compulsion.

10.2. Consent and information to the patient

The sponsor will submit in writing to the Ethical Committee of the Sponsor's company the model of informed consent, according to the guidelines of the ICH and the legal requirements, for its review and approval before the start of the study. Before includingsubjects into the study, a copy of the informed consent approved by the Ethical Committee of the Sponsor's company will be reviewed with the possible participant, and it will be signed and dated. The investigator will provide a copy of the signed informed consent form for each subject and keep a copy in the subject's study file.

11. DATA COLLECTION NOTEBOOK

The study data must be verifiable with the source data, which requires access to all original records, laboratory reports and subject records. Therefore, the researcher must agree to allow access to subject records and the source data must be available for all study data. The subjects or their legal representatives must also allow access to the medical records of the subjects and they will be informed of this need and will express their agreement when they provide the informed consent.

The person in charge of data registration in the Spanish Data Protection Agency will be Bionos Biotech S.L. The selection register of Bionos Biotech S.L. (name of file "Voluntarios Bionos") will collect pertinent information on all selected subjects, in compliance with the Organic Law of Protection of Personal Data (LOPD). The sponsor of the study will not have access to the personal data of the volunteers, only to the studydata that will be anonymized.

A data collection notebook will not be completed for subjects that are faults in the selection. A data collection notebook will be completed for subjects moving toward randomization until the end of the study or the end of the study.

12. CONSERVATION OF RECORDS

The investigator must keep records of the disposition of the tested product/s, subject exclusion records, signed informed consents, data collection networks, all correspondence and supporting documentation.

13. PUBLICATION POLICY

The ownership of the data corresponds exclusively to the sponsor, who reserves the right to use them and their publication.

The sponsor may intend to publish the results of this study in a medical journal. No researcher may publish on their own the results of this study or those of their own patients, without the prior authorization of the sponsor.

14. ECONOMIC REPORT

Each of the volunteers will be compensated with the amount of TWENTY (20) euros, which will be delivered in a single final payment, if the study obligations have been fulfilled.

The research team will not receive additional economic benefit, since the developmentof this type of study is an obligation included in their salary.

15. REFERENCES

1. Al-Atif, H. Collagen Supplements for Aging and Wrinkles: A Paradigm Shift in the Fields of Dermatology and Cosmetics. *Dermatol Pract Concept* **12**, e2022018 (2022).
2. Alcock, R. D., Shaw, G. C., Tee, N., Welvaert, M. & Burke, L. M. Urinary Hydroxyproline Is Only Suitable As a Biomarker for Acute Intake, Up to 6 hr Postingestion of Collagen Proteins in 'Free-Living,' Healthy, Active Males. *Int J Sport Nutr Exerc Metab* **29**, 461–465 (2019).
3. Shigemura, Y., Kubomura, D., Sato, Y. & Sato, K. Dose-dependent changes in the levels of free and peptide forms of hydroxyproline in human plasma after collagen hydrolysate ingestion. *Food Chem* **159**, 328–332 (2014).
4. Oikawa, S. Y. *et al.* A randomized controlled trial of the impact of protein supplementation on leg lean mass and integrated muscle protein synthesis during inactivity and energy restriction in older persons. *Am J Clin Nutr* **108**, 1060–1068 (2018).
5. Giglio, B. M. *et al.* Whey Protein Supplementation Compared to Collagen Increases Blood Nesfatin Concentrations and Decreases Android Fat in Overweight Women: A Randomized Double-Blind Study. *Nutrients* **11**, E2051 (2019).
6. Ichikawa, S. *et al.* Hydroxyproline-containing dipeptides and tripeptides quantified at high concentration in human blood after oral administration of gelatin hydrolysate. *Int J Food Sci Nutr* **61**, 52–60 (2010).
7. Ohara, H., Matsumoto, H., Ito, K., Iwai, K. & Sato, K. Comparison of quantity and structures of hydroxyproline-containing peptides in human blood after oral ingestion of gelatin hydrolysates from different sources. *J Agric Food Chem* **55**, 1532–1535 (2007).
8. Avila Rodríguez, M. I., Rodríguez Barroso, L. G. & Sánchez, M. L. Collagen: A review on its sources and potential cosmetic applications. *J Cosmet Dermatol* **17**, 20–26 (2018).
9. Liu, D., Nikoo, M., Boran, G., Zhou, P. & Regenstein, J. M. Collagen and gelatin. *Annu Rev Food Sci Technol* **6**, 527–557 (2015).
